# Stem cells are the most sensitive screening tool to identify toxicity of GATA4-targeted novel small-molecule compounds

**DOI:** 10.1007/s00204-018-2257-1

**Published:** 2018-07-09

**Authors:** S. Tuuli Karhu, Mika J. Välimäki, Mikael Jumppanen, Sini M. Kinnunen, Lotta Pohjolainen, Robert S. Leigh, Samuli Auno, Gábor Földes, Gustav Boije af Gennäs, Jari Yli-Kauhaluoma, Heikki Ruskoaho, Virpi Talman

**Affiliations:** 1 Drug Research Program and Division of Pharmacology and Pharmacotherapy, Faculty of Pharmacy, University of Helsinki, P.O. Box 56, 00014 Helsinki, Finland; 2 Department of Pharmacology and Toxicology, Institute of Biomedicine, University of Oulu, Oulu, Finland; 3 Drug Research Program and Division of Pharmaceutical Chemistry and Technology, Faculty of Pharmacy, University of Helsinki, P.O. Box 56, 00014 Helsinki, Finland; 4 National Heart and Lung Institute, Imperial College London, London, UK; 5 Heart and Vascular Center, Semmelweis University, Budapest, Hungary

**Keywords:** Toxicity screening, High-content analysis, Structure–toxicity relationship, Stem cells, Cardiomyocytes, Isoxazole derivatives

## Abstract

Safety assessment of drug candidates in numerous in vitro and experimental animal models is expensive, time consuming and animal intensive. More thorough toxicity profiling already in the early drug discovery projects using human cell models, which more closely resemble the physiological cell types, would help to decrease drug development costs. In this study we aimed to compare different cardiac and stem cell models for in vitro toxicity testing and to elucidate structure–toxicity relationships of novel compounds targeting the cardiac transcription factor GATA4. By screening the effects of eight compounds at concentrations ranging from 10 nM up to 30 µM on the viability of eight different cell types, we identified significant cell type- and structure-dependent toxicity profiles. We further characterized two compounds in more detail using high-content analysis. The results highlight the importance of cell type selection for toxicity screening and indicate that stem cells represent the most sensitive screening model, which can detect toxicity that may otherwise remain unnoticed. Furthermore, our structure–toxicity analysis reveals a characteristic dihedral angle in the GATA4-targeted compounds that causes stem cell toxicity and thus helps to direct further drug development efforts towards non-toxic derivatives.

**Electronic supplementary material:**

The online version of this article (10.1007/s00204-018-2257-1) contains supplementary material, which is available to authorized users.

## Introduction

Early drug discovery projects routinely use tumor-derived or genetically immortalized cell lines and primary cells from experimental animals to assess toxicity of novel compounds (Horvath et al. 2016). The cell type chosen for toxicity screening may influence the results and a wrong choice can lead to misjudgements in lead selection. Most often toxic compounds are identified during preclinical drug development, but sometimes, for example due to species differences, they may only come up in the clinical phase or during post-approval follow-up with detrimental results (Kerbrat et al. 2016; Suntharalingam et al. 2006; Wysowski et al. 2001). More precise in vitro toxicity assessment using human cell models that more closely resemble physiological cell types is, therefore, urgently needed to improve the validity of results and to reduce drug discovery costs and the use of experimental animals (Sison-Young et al. 2012).

The recent progress in human stem cell-based models, including the discovery of human induced pluripotent stem cell (hiPSC) technology (Takahashi et al. 2007; Yu et al. 2007), enables the production of almost any human cell type in the laboratory. This provides a unique possibility to carry out drug screening and toxicity studies using differentiated human cells that cannot be readily obtained from living persons, such as cardiomyocytes and neurons, and eliminates the influence of interspecies differences. Additionally, pluripotent stem cells have been accepted as a validated model for evaluating reproductive and developmental toxicity in vitro (Seiler and Spielmann 2011).

The cardiac transcription factor GATA4 is a master regulator of cardiogenesis (Pikkarainen et al. 2004). It regulates cardiac development, cardiomyocyte hypertrophy, cardiomyocyte survival and cardiac regeneration (Gupta et al. 2013; Kikuchi et al. 2010; Malek Mohammadi et al. 2017; Pikkarainen et al. 2003; Rysä et al. 2010). GATA4 interacts directly with other cardiac transcription factors, such as NKX2-5 and TBX5, to cooperatively regulate gene expression (Ang et al. 2016; Kinnunen et al. 2015; Luna-Zurita et al. 2016; Pikkarainen et al. 2004). GATA4 mutations that affect its transcriptional activity or interactions with its cofactors can lead to cardiomyopathies and congenital heart defects (Ang et al. 2016; Garg et al. 2003; Li et al. 2013). Cardiac transcription factors and their protein–protein interactions can thus be considered attractive, yet challenging, drug targets.

The activities of both GATA4 and NKX2-5 are enhanced by hypertrophic stimuli (Kohli et al. 2011). GATA4 and NKX2-5 act synergistically as crucial controllers of, for instance, atrial natriuretic peptide (ANP) transcription (Lee et al. 1998). Additionally, the GATA4 consensus sites in combination with an NKX2-5 binding element are essential for stretch-activated B-type natriuretic peptide (BNP) transcription and cardiomyocyte hypertrophy (Pikkarainen et al. 2003). We have recently reported novel compounds that target GATA4 and its interaction with NKX2-5 (Välimäki et al. 2017). The hit compound 3i-1000 [compound **3** in our previous report (Välimäki et al. 2017)] inhibits GATA4-induced synergistic transactivation of a promoter sequence containing NKX2-5 binding sites and attenuates stretch-induced cardiomyocyte hypertrophy. The compound also inhibits pro-hypertrophic extracellular signal-regulated kinase 1/2 signalling in the hypoxic endocardial layer of the left ventricle in vivo after systemic administration in heart-targeted nanoparticles (Ferreira et al. 2017) and improves cardiac function after myocardial infarction or angiotensin II-induced cardiac hypertrophy in vivo (Kinnunen et al. 2018).

**Fig. 1:**
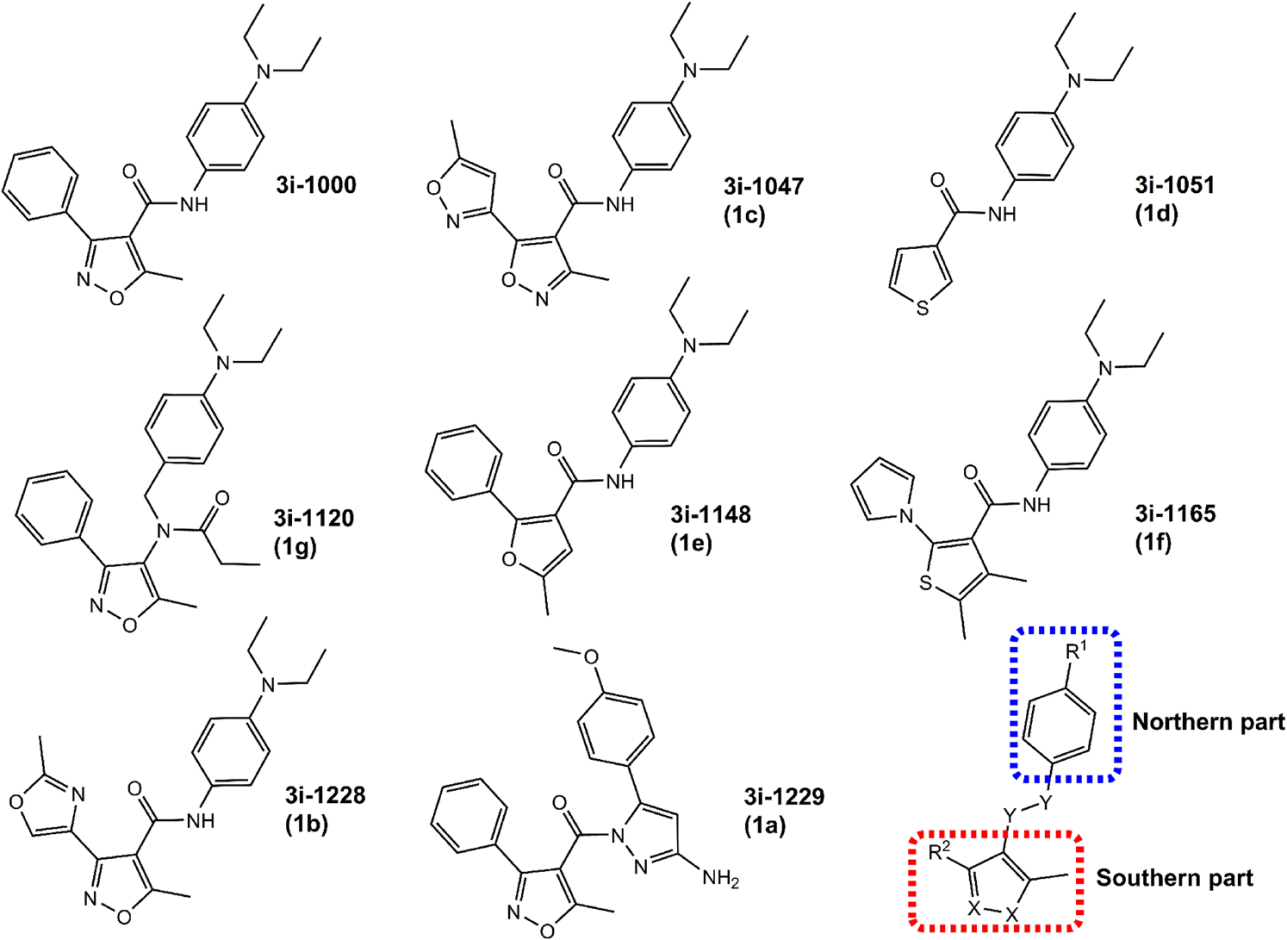
Compound structures. Chemical structures of the active compound 3i-1000 and its derivatives analysed herein. These molecules can formally be characterized by northern and southern parts that are connected via a linker unit as defined in the figure. The northern part is typically comprised of a *para*-phenylenediamine, whereas the southern part is typically comprised of an *ortho*-aryl-substituted heterocycle. The linker unit is a two-atom spacer consisting of a nitrogen atom and *sp*^2^ or *sp*^3^ carbon atom

The aims of this study were (1) to compare different cardiac and stem cell types in toxicity screening and (2) to investigate the in vitro toxicity and structure–toxicity relationships of a new family of compounds targeting GATA4 and its interaction with NKX2-5. Toxicity of a structurally conserved set of eight compounds (Fig. 1) was investigated using various cardiac and non-cardiac cell models: H9c2 myoblast cell line derived from rat myocardium, primary neonatal rat ventricular cardiomyocytes (NRVCs), primary neonatal rat cardiac fibroblasts (CFs), mouse embryonic fibroblasts (MEFs), mouse embryonic stem cells (mESCs), mESC derivatives from day 5 embryoid bodies (D5EBs), hiPSCs and hiPSC-derived cardiomyocytes (hiPSC-CMs). Cell viability and toxicity were assessed using the lactate dehydrogenase (LDH) assay and the 3-(4,5-dimethylthiazol-2-yl)-2,5-diphenyltetrazolium (MTT) bromide assay to investigate necrosis and mitochondrial redox metabolism, respectively. Two compounds were analysed in more detail in hiPSCs and hiPSC-CMs using high-content analysis (HCA). The results indicate that the cell types selected for toxicity screening have a major effect on the results. In addition, the data reveal structural features causing stem cell toxicity in the 3i-1000 family of compounds.

## Materials and methods

### Compounds and reagents

The lead compound 3i-1000 was purchased from Pharmatory Ltd (Oulu, Finland). All other compounds targeting GATA4 and its interaction with NKX2-5 were synthesized at the Division of Pharmaceutical Chemistry and Technology, Faculty of Pharmacy, University of Helsinki (Finland), as described below. Cell culture media, supplements and reagents were purchased from Gibco (Thermo Fisher Scientific, Paisley, UK) unless otherwise stated. Dulbecco’s modified Eagle medium (DMEM), bovine serum albumin, insulin–transferrin–sodium selenite media supplement, sodium pyruvate, pancreatin, 4′,6-diamidino-2-phenylindole (DAPI) and all reagents used in cytotoxicity assays were purchased from Sigma-Aldrich (Steinheim, Germany). Collagenase type II was purchased from Worthington Biochemical Corporation (Lakewood, NJ, USA). Growth Factor-Reduced Matrigel^®^ was acquired from Corning (Bedford, MA, USA), and small-molecule inhibitors Y-27632, CHIR99021 and Wnt-C59 were bought from Tocris Bioscience (Bristol, UK). Leukemia inhibitory factor (LIF), as well as gelatin and fetal bovine serum (FBS) used in mESC and MEF cultures, were from Merck Millipore. 5-Bromo-2′-deoxyuridine (BrdU) was purchased from Abcam (Cambridge, UK). The primary antibodies used in immunofluorescence stainings were: polyclonal rabbit anti-Ki67 (Abcam), monoclonal rat anti-BrdU (Abcam), and monoclonal mouse anti-α-actinin (Sigma-Aldrich). The secondary antibodies used were all purchased from Life Technologies (Eugene, OR, USA): Alexa Fluor 488 goat anti-mouse lgG, Alexa Fluor 546 donkey anti-rabbit lgG, and Alexa Fluor 647 goat anti-rat lgG. CellEvent™ Caspase-3/7 Green Detection Reagent, MitoTracker^®^ Orange CMTMRos probe, as well as Hoechst 33342 were from Invitrogen (Carlsbad, CA, USA).

### Synthesis of compounds

**Fig. 2:**
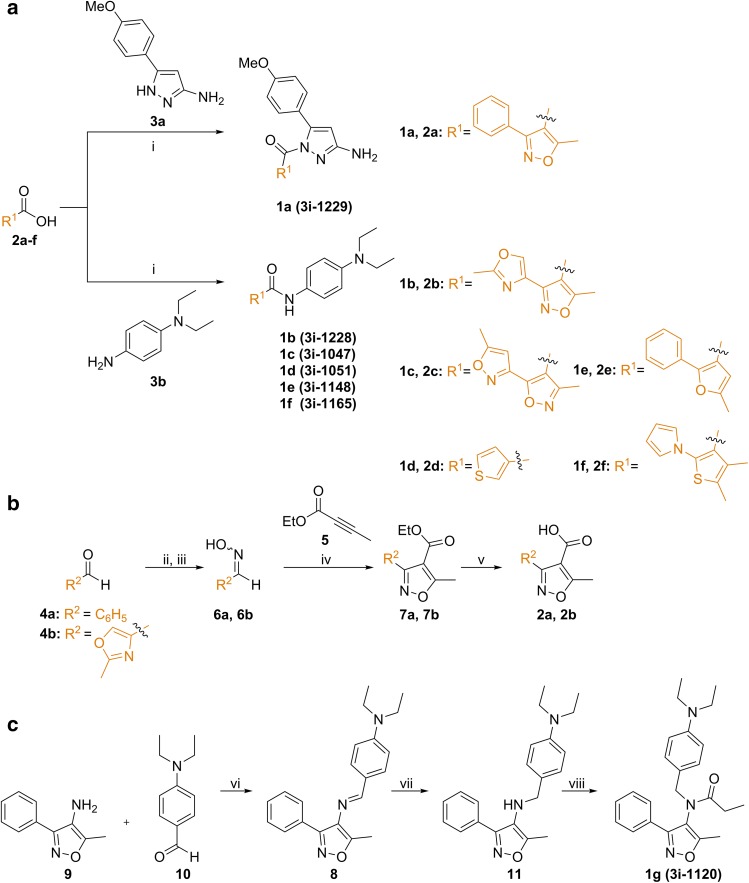
Synthesis of compounds. **a** Synthesis of amides. Reagents and conditions: (i) HBTU, DIPEA, DMF, rt, 1 d, 49–73%. **b** Synthesis of the carboxylic acid intermediates. Reagents and conditions: (ii) H_2_NOH·HCl, pyridine, EtOH; (iii) H_2_NOH·HCl, NaOAc; (iv) Oxone^®^, KCl, H_2_O; (v) HTIB, H_2_O. **c** Synthesis of the *N,N*-disubstituted amide via imine formation. Reagents and conditions: (vi) AcOH, Na_2_SO_4_ (anhydr.) rt, overnight, 74%; (vii) NaBH_4_, MeOH, THF, rt, 2 d, 37%; (viii) EtCOCl, DMAP, pyridine, rt, 3 d, 74%

The synthesis of compounds (**1a**–**g**) is described in Fig. 2 and details are provided in Supplementary Information. The compounds **1a**–**f** were synthesized via *O*-(benzotriazol-1-yl)-*N,N,N′,N′*-tetramethyluronium hexafluorophosphate (HBTU)-mediated amide coupling of various carboxylic acids (**2a**–**f**) and aminopyrazole **3a** or aniline **3b** (Fig. 2a). The yields were moderate to good (49–73%). There can be at least two reasons for slightly lower than expected yields of amides: first, the nucleophilicity of the aniline-type starting materials such as compound **3b** is reduced compared to aliphatic amines; and second, the heterocyclic carboxylic acid starting materials (**2a**–**f**) are sterically hindered.

The used carboxylic acids were either commercially available (**2c**–**f**) or synthesized via [3 + 2] dipolar cycloaddition reactions (**2a, 2b**) starting from affordable and commercially available aldehydes (**4a, 4b**) and alkyne **5** (Fig. 2b). The regiochemistry of the isoxazole-forming reaction is dominated by the electrophilicity of the alkyne (Houk et al. 1973). Synthesis of the phenyl-substituted isoxazolecarboxylic acid **2a** started from benzaldehyde **4a** by converting it to the oxime **6a** in the presence of hydroxylammonium chloride and pyridine. The resulting oxime **6a** was subjected to Oxone^®^-mediated cycloaddition reaction (Han et al. 2014) in the presence of ethyl 2-butynoate **5** and a catalytic amount of potassium chloride. Hydrolysis of the resulting ester **7a** with aqueous sodium hydroxide gave the phenyl-substituted isoxazolecarboxylic acid **2a**.

For the synthesis of the 2-methyloxazole-substituted isoxazolecarboxylic acid **2b** a slightly different approach was used. The corresponding oxime **6b** was prepared from 2-methyloxazole-4-carbaldehyde **4b** by treatment with hydroxylammonium chloride in the presence of sodium acetate. The carboxylic acid **2b** was prepared from the oxime **6b** in the presence of ethyl 2-butynoate **5** using [hydroxy(tosyloxy)iodo]benzene (HTIB)-mediated cycloaddition reaction in water followed by hydrolysis of the formed ester **7b** with aqueous sodium hydroxide (Raihan et al. 2010). Water has been reported to be a good solvent for dipolar cycloaddition reactions (Butler and Coyne 2010; Kesornpun et al. 2016). Both of the prepared carboxylic acids (**2a, 2b**) were used in the final amide coupling step (Fig. 2a) without further purification.

Preparation of the *N,N*-disubstituted amine **1g** (Fig. 2c) is based on the initial imine (**8**) formation between isoxazolyl amine **9** and 4-(diethylamino)benzaldehyde **10** in the presence of acetic acid and anhydrous Na_2_SO_4_ followed by reduction of the carbon–nitrogen double bond with NaBH_4_. The resulting amine **11** was coupled with propionyl chloride in the presence of 4-(dimethylamino)pyridine (DMAP) to give the desired amine **1g** in good yield.

### Cell cultures

COS-1 cells were used to investigate the compound effects on GATA4–NKX2-5 synergistic gene activation using a luciferase reporter assay (Kinnunen et al. 2015). Toxicity profiling was carried out in eight cell types: the H9c2 myoblast cell line, NRVCs, CFs, mESCs, MEFs, mESC derivatives from day 5 embryoid bodies (D5EBs), hiPSCs and hiPSC-CMs. All cell cultures were maintained at 37 °C in a humidified atmosphere of 5% carbon dioxide (CO_2_) and the regular growth medium was used for compound exposures. Cell culture methods and cardiomyocyte differentiation from human pluripotent stem cells are described in Supplementary Information.

### Plasmids

The empty expression plasmid MT2 (pMT2) and mouse Gata4 expressing pMT2-Gata4 plasmids were gifts from D. B. Wilson (Department of Pediatrics, St. Louis Children’s Hospital) (Arceci et al. 1993). The luciferase reporter plasmid pGL3-3xHA containing three Nkx2-5 high-affinity binding sites has been described previously (Kinnunen et al. 2015). To create mouse Nkx2-5 expressing pMT2-Nkx2-5, the cDNA was cloned from pFLCI-Nkx2-5 (clone D330001C20, Imagenes), which was a kind gift from R. Kerkelä (University of Oulu, Finland), with primers containing the EcoRI restriction site (forward: 5′-ATATATGAATTCGTCGCCACCATGTTCCCCAG-3′ and reverse 5′-ATATATGAATTCCTACCAGGCTCGGATGCCGTGC-3′) and ligated to pMT2 EcoRI site. The correct clone was identified by digestion with specific restriction enzymes and the sequence was verified by DNA sequencing (DNA Sequencing and Genomics Laboratory, Institute of Biotechnology, University of Helsinki, Finland).

### Luciferase assays

COS-1 cells grown on 96-well plates were transfected with the luciferase reporter plasmid pGL3-3xHA (100 ng/well) and equal amounts of expression vectors pMT2-Gata4 and pMT2-Nkx2-5 (total of 50 ng/well) using Fugene 6 (Promega) at a reagent:DNA ratio of 3:1. Cells transfected with either pMT2, pMT2-Gata4 or pMT2-Nkx2-5 were used to confirm synergistic transactivation of the reporter gene in all experiments. After 6 h of transfection, the cells were treated with compounds or vehicle (0.1% dimethyl sulfoxide, DMSO) and the luminescence was measured 24 h later using Neolite Reporter Gene Assay System (PerkinElmer, Turku, Finland) according to manufacturer’s instructions and Victor2 plate reader (PerkinElmer, Turku, Finland). Each compound was tested in three technical replicates in at least two independent experiments.

### Cytotoxicity assays

The cells were exposed to the test compounds for 24 h and cytotoxicity was quantified with MTT and LDH assays as described previously (Talman et al. 2011). For the determination of cell membrane integrity, 50 µl of medium from each well was transferred onto a new 96-well plate followed by addition of 50 µl of substrate solution containing 1.3 mM β-nicotinamide adenine dinucleotide, 660 µM iodonitrotetrazolium, 54 mM l(+)-lactic acid, 280 µM phenazine methosulphate and 0.2 M Tris–HCl (pH 8.0). After a 30-min incubation, the reaction was stopped by adding 50 µl of 1 M acetic acid to each well and absorbance was measured at 490 nm using Victor2 plate reader (PerkinElmer, Turku, Finland). Spontaneous LDH release was measured from untreated cells, maximal LDH release from cells lysed with 0.9% Triton X-100 and background absorbance was measured from wells without cells. After background extraction, cytotoxicity was calculated as follows: cytotoxicity (%) = [(sample − spontaneous LDH release)/(maximal LDH release − spontaneous LDH release)] × 100. For quantification of metabolic activity, 3-(4,5-dimethylthiazol-2-yl)-2,5-diphenyltetrazolium bromide (MTT) was added to the cells at a final concentration of 0.5 mg/ml followed by 2 h incubation in cell culture conditions. The medium was then aspirated and formazan crystals were solubilized in DMSO. Absorbance was measured at 550 nm and absorbance at 650 nm was subtracted as background.

### Computational chemistry

Commercial modeling package MOE 2014.09 (Chemical Computing Group Inc., Montreal, Canada; http://www.chemcomp.com) with LowModeMD module was utilized to generate small-molecule conformation databases (Labute 2010). Force fields suitable for small molecules (MMFF94x and OPLS-AA) were applied for the molecule parameterizations and energy minimizations. Moreover, the default settings were employed to score and rank the conformational databases. The lowest energy conformation was selected to define the torsion angle between the ring planes in the southern part of the compound. Mogul v.1.7.2 software utilizing knowledge-based library of molecular geometries from the Cambridge Structural Database (CSD) was applied to provide precise experimentally derived information about preferred ring system geometries of 3i-1000 and 3i-1047 (Bruno et al. 2004).

### Automated fluorescence microscopy and high-content analysis

For proliferation studies, the cells were exposed to the test compounds for 24 h and 10 µM BrdU was added to the culture medium for the last 1 h (hiPSCs) or 24 h (hiPSC-CMs) before fixation with 4% paraformaldehyde (PFA) for 15 min at room temperature (rt). The cells were then permeabilized with 0.1% Triton X-100 for 10 min and DNA was hydrolysed with 2 M hydrochloric acid for 30 min at rt followed by neutralization with 0.1 M sodium borate (pH 8.5) for 30 min. Non-specific binding sites were blocked with 4% FBS in phosphate-buffered saline (PBS) for 45 min at rt, whereafter the cells were incubated with anti-Ki67 (1:250) and anti-BrdU (1:250) antibodies for 60 min at rt. Cells were then washed 3 × 5 min with PBS and incubated with Alexa Fluor-conjugated secondary antibodies (1:200) and DAPI (5 µg/ml) for 45 min at rt. In hiPSC-CM cultures, staining with primary antibody against α-actinin (at 1:600) was additionally used to identify myocytes. The plates were imaged and analysed with CellInsight CX5 High Content Screening Platform (Thermo Scientific) using a 10× objective (Olympus UPlanFL N 10×/0.3). For quantification, the cells were first identified based on DAPI fluorescence, which defined the nuclear area for quantifications. In hiPSC-CM cultures, non-myocytes were excluded based on absence of α-actinin staining and nuclear area. The thresholds for α-actinin fluorescence intensity and nuclear area were set manually in each experiment to allow optimal exclusion of non-myocytes. In hiPSC-CMs cultures, the data were collected only from α-actinin positive cells. The thresholds for Ki67+ and BrdU+ cells were also set manually in each experiment as described previously (Mioulane et al. 2012) to adjust for slight variation in staining intensity.

To study mitochondrial function using high-content analysis, the cells were plated and treated as in proliferation studies, except that MitoTracker Orange CMTMRos solution (400 nM) was added to the cells for the last 30 min at 37 °C. The accumulation of the MitoTracker probe used is dependent upon mitochondrial membrane potential allowing selective staining of active mitochondria. Following fixation and permeabilization, hiPSC-CMs were in addition stained with anti-α-actinin (Alexa Fluor 488) and DAPI. Cells were identified and hiPSC-CMs discriminated from non-myocytes as described above. MitoTracker intensity corresponding to active mitochondria was analysed in the perinuclear area defined by a 3-pixel ring or a 5-pixel ring around the nucleus in hiPSCs and hiPSC-CMs, respectively. The fluorescence intensity threshold was set manually in each experiment based on the assumption that 2% of control hiPSC-CMs and 5% of control hiPSCs were MitoTracker negative.

To study caspase activation, the cells were exposed to the test compounds for 4 h and then incubated with 3–5 µM CellEvent™ Caspase-3/7 Green Detection Reagent solution in PBS with 5% FBS for 50 min and stained with Hoechst for 10 min at 37 °C. Cells were fixed with 4% PFA and scanned with CellInsight using the 10× objective, or alternatively, the staining solution was replaced with fresh 5% FBS in PBS followed by live cell imaging with CellInsight. Hoechst fluorescence was used to identify the cells and define the nuclear area. The intensity of green fluorescence within the nucleus was quantified as a measure of caspase 3/7 activity. The fluorescence intensity threshold for caspase positive and caspase negative cells was set manually in each experiment based on the assumption that 5% of control hiPSCs exhibited caspase 3/7 activity.

### Statistics

Results are expressed as mean + SEM from at least three independent experiments. Data were analysed using IBM SPSS Statistics 24 software. Homogeneity of variances was tested using Levene’s test. If the data met the assumptions of the test, one-way ANOVA and a Tukey HSD post hoc test were carried out; otherwise Welch ANOVA and a Games–Howell post hoc test were performed. Additionally, factorial ANOVA was carried out for hiPSC viability data. Differences at the level of *P* < 0.05 were considered statistically significant.

## Results

### Effects on GATA4–NKX2-5 interaction

**Fig. 3:**
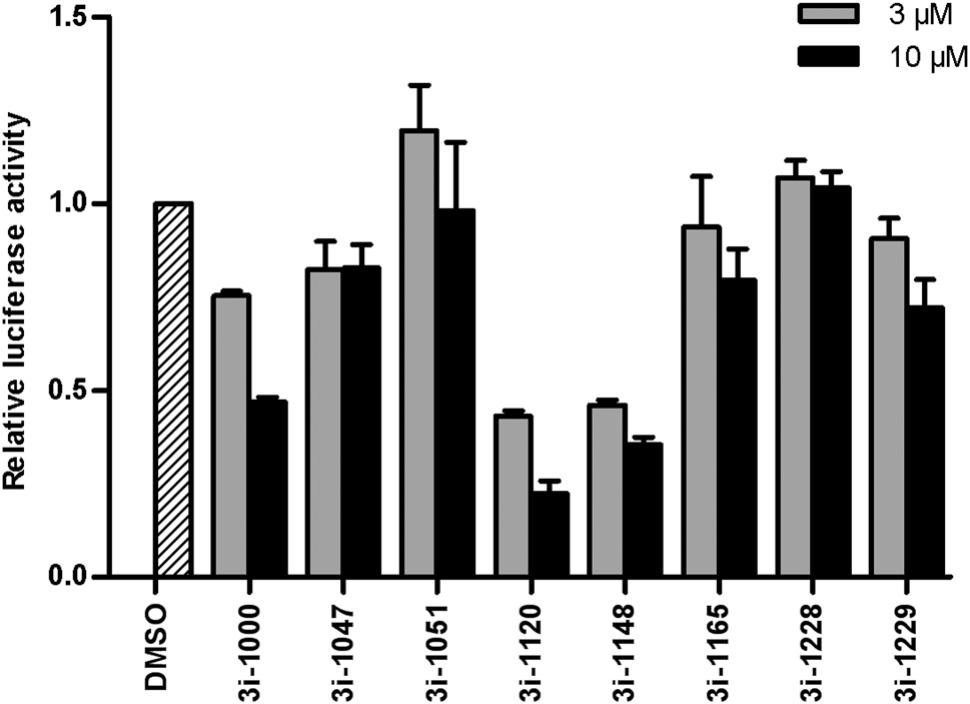
Effects of the small-molecule compounds on GATA4- and NKX2-5-induced synergistic gene activation. Luciferase reporter activity was normalized to vehicle (DMSO) control and is presented as mean + SEM (*n* = 2–3, apart from 3i-1000; *n* = 13)

A luciferase reporter assay with an artificial promoter containing three high-affinity binding sites for NKX2-5 was used to evaluate the effects of the compounds on GATA4–NKX2-5 transcriptional synergy (Kinnunen et al. 2015). We have previously shown that while GATA4 alone induces only minimal activation of this promoter, it strongly potentiates NKX2-5-mediated transactivation (Kinnunen et al. 2015; Välimäki et al. 2017). Compounds 3i-1000, 3i-1120 and 3i-1148 exhibited an inhibitory effect on GATA4- and NKX2-5-induced gene activation, whereas compounds 3i-1047, 3i-1165 and 3i-1229 had only a weak effect and compounds 3i-1051 and 3i-1228 were ineffective (Fig. 3).

### The GATA4-targeted compounds do not induce cell necrosis

When exposed to the test compounds for 24 h, none of the cell types investigated exhibited a significant necrotic response as measured by LDH release into the culture medium (Supplementary Fig. S1). More specifically, compound-induced cytotoxicity was always less than 15% of the maximal LDH release regardless of the compound and the cell type.

### Toxicity of GATA4-targeted compounds is cell type-specific

**Fig. 4:**
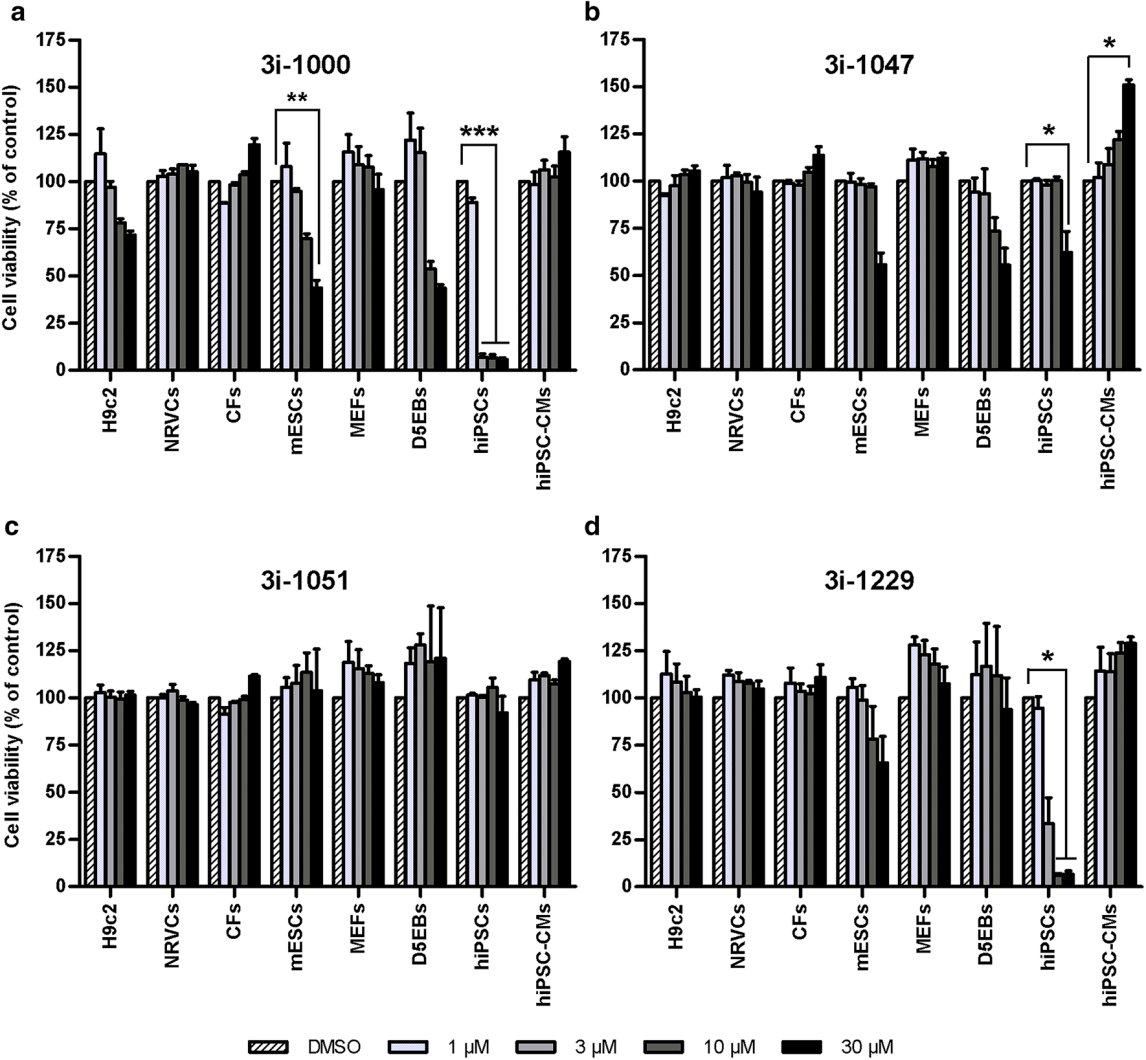
Cell type-dependent toxicity of GATA4-targeted compounds. The results show the effects of **a** 3i-1000, **b** 3i-1047, **c** 3i-1051, and **d** 3i-1229 on the viability of all eight cell types tested. Cell viability was determined with the MTT assay after a 24-h compound exposure. Results are expressed as mean + SEM (*n* = 3–4). ***P < 0.001 vs. DMSO; **P < 0.01 vs. DMSO; *P < 0.05 vs. DMSO (one-way ANOVA followed by Tukey’s HSD or Welch ANOVA followed by Games–Howell)

**Fig. 5:**
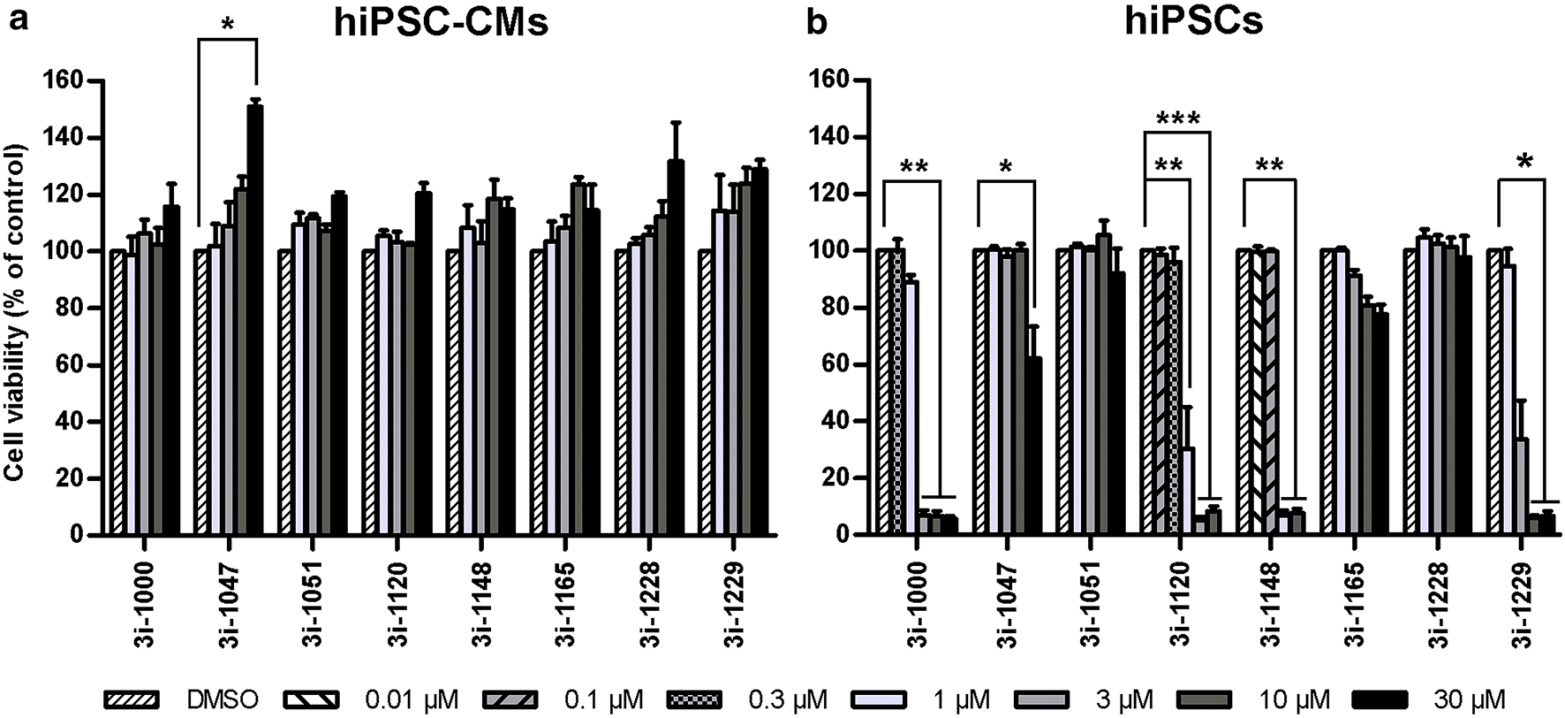
Effects of the test compounds on **a** hiPSC-CM and **b** hiPSC viability. Cell viability was determined with the MTT assay after a 24-h compound exposure. Results are expressed as mean + SEM (*n* = 3–4). ***P < 0.001 vs. DMSO; **P < 0.01 vs. DMSO; *P < 0.05 vs. DMSO (one-way ANOVA followed by Tukey’s HSD or Welch ANOVA followed by Games–Howell)

In the MTT assay, the effects of individual compounds on cell viability differed substantially between the cell types studied as exemplified by compounds 3i-1000, 3i-1047, 3i-1051 and 3i-1229 (Fig. 4). For all compounds tested, a comparison between hiPSCs and hiPSC-CMs is presented in Fig. 5 and for other cell types in Supplementary Figure S2. Overall, cardiomyocytes, fibroblasts, and H9c2 cells were the most resistant cell types, as none of the compounds induced significant toxicity in these cells. The two fibroblast types (CFs and MEFs) exhibited almost identical responses to the small molecules: none of the compounds had a considerable effect on CF viability, while only 3i-1120 at 30 µM induced modest toxicity in MEFs (Supplementary Fig. S2; not statistically significant). Also the two cardiomyocyte types, hiPSC-CMs and NRVCs, acted comparably: none of the compounds caused toxicity in either of the cardiomyocyte types. However, 3i-1047 induced a 51% increase in hiPSC-CM metabolic activity (*P* = 0.033), which was not observed in NRVCs (Fig. 4b). The mechanism(s) of this increase remain to be established, but it might be due to increased cell proliferation, higher mitochondrial content or increased metabolic activity.

Unlike in cardiomyocytes, fibroblasts and H9c2 cells, significant toxicity was observed with several of the test compounds in mESCs, hiPSCs and D5EBs (Fig. 4, Supplementary Fig. S2). As an example, 3i-1000 induced profound reductions in cell viability in mESCs (56% at 30 µM, *P* = 0.006) and hiPSCs (93–94% at ≥ 3 µM, *P* < 0.001), as well as a moderate, but not statistically significant (*P* = 0.088) reduction in metabolic activity of D5EBs at ≥ 10 µM (Fig. 4a). As for 3i-1047, smaller 38–45% reductions in the viability of mESCs (*P* = 0.100), D5EBs (*P* = 0.612) and hiPSCs (*P* = 0.021) were observed, and this was apparent only at the highest concentration of 30 µM (Fig. 4b). These cell types were also sensitive to compounds 3i-1120, 3i-1148 and 3i-1229 (Fig. 5b; Supplementary Fig. S2). In general, the hiPSCs were the most sensitive cell type based on the IC_50_ values (3i-1000, 1.46 µM; 3i-1120, 0.60 µM; 3i-1148, 0.30 µM, and 3i-1229, 2.07 µM) and on the maximal toxicity induced at the highest concentration tested. The effects of both treatment and dose, as well as the treatment–dose interaction were all statistically significant (*P* < 0.001, factorial ANOVA).

### Structure–toxicity relationships

When comparing the effects of different compounds within each individual cell type, the compounds can be grouped into two main classes: toxic (3i-1000, 3i-1120, 3i-1148 and 3i-1229) and non-toxic (3i-1047, 3i-1051, 3i-1165, and 3i-1228) compounds. Based on the hiPSC toxicity data, 3i-1148 can be considered as the most toxic compound with an IC_50_ of 0.30 µM (Fig. 5b). Structurally, all of the derivatives of 3i-1000, despite the fact that they all possess unique structural modification in the southern, middle or northern part of the compound, were classified as toxic compounds based on their effects on stem cell and progenitor cell viability. Surprisingly, toxicity was preserved despite the extensive structural modifications in the middle and northern parts (3i-1120 and 3i-1229, respectively). These findings suggest that the southern part of the compound is predominantly responsible for the toxic outcome in stem cells.

**Fig. 6:**
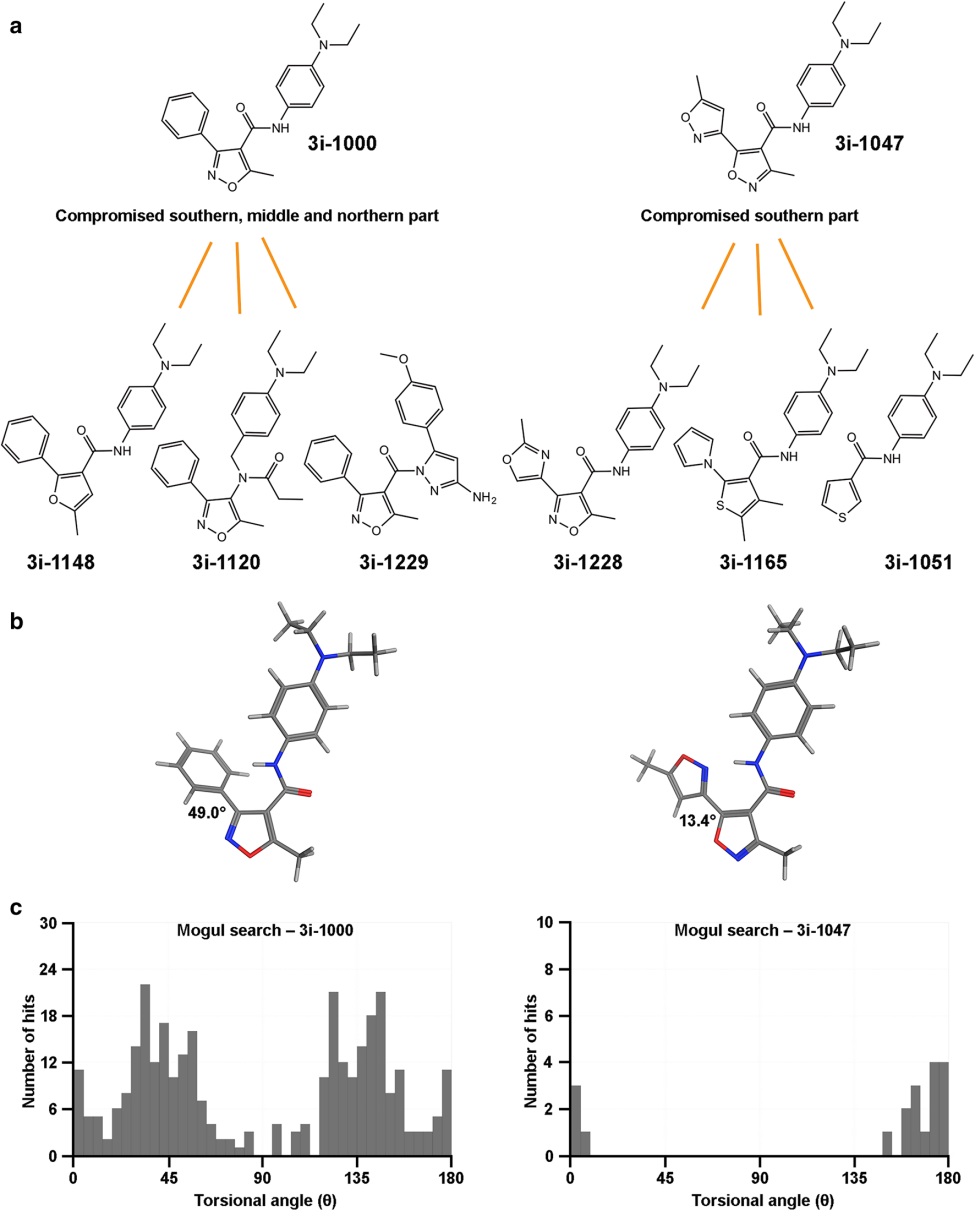
Structure-based toxicity relay on the consistent conformational geometry identified in the southern part of the 3i-1000 and related compounds. **a** Test compounds were classified into two structural categories omitting a five-membered or a six-membered ring bound to the isoxazole (3i-1000 or 3i-1047 families). **b** Force field-based calculations (MMFF94x) revealed family-correlated conformations for the representative compounds 3i-1000 and 3i-1047. **c** Knowledge-based conformational analysis with Mogul (Cambridge Structural Database) suggests unique set of torsion angles for both compound families

To characterize the structural determinants of toxicity, a force field-based conformational search was carried out (Labute 2010). The analysis suggests that the torsional angles for the ring system geometry in the southern part are distinct for the 3i-1000 and 3i-1047 compound families (Fig. 6). Two different force fields (MMFF94x and OPLS-AA) suitable for small organic compounds were applied for the parametrization of the molecule structures. The six-membered ring bound to isoxazoles of the 3i-1000 family showed consistent torsional angles from 28° to 51° for the ring geometry with both tested force field settings, whereas, the five-membered ring bound to isoxazoles of the 3i-1047-family preferred a flatter orientation of the ring system in the southern part with values ranging from 0° to 19°. In this context, the ability of the compounds to reach both the coplanar orientation and the global low energy conformation is defined by steric factors linked to the internal energy of the compounds. For instance, steric hindrance in the 3i-1000 family of compounds, with two C–H bonds (*ortho* position) in the six-membered phenyl ring, enforces larger dihedral angles into the ring system due to overlapping electron clouds and associated increase in internal energy. However, in case of compounds with a five-membered ring (3i-1047 family), the critical molecular area is less crowded and allows the compounds to adopt a periplanar, and in the case of 3i-1228, almost a coplanar orientation without extra cost in energy. Moreover, because of the presence of a heteroatom in the five-membered ring of the 3i-1047 family compounds, an additional intramolecular hydrogen bond is formed and associated favorably to the lowest energy conformations.

Parallel conformational evaluation of ring orientations in the southern part was carried out with knowledge-based approach relying on data derived from small-molecule crystal structures. Conformational analysis with Mogul (Cambridge Crystallographic Data Center) provides experimentally validated approximation of the specific torsion angle for the ring systems in the southern part (Bruno et al. 2004). The data suggest that the 3i-1047 compound family with a five-membered ring in the southern part adopts a significantly flatter ring geometry in comparison to the six-membered ring systems in the 3i-1000 compound family. This is in agreement with the conformation analysis measured by force field methods and correlates directly with stem cell toxicity observed with the 3i-1000 family of GATA4-targeted compounds.

### High-content analysis of cell viability

Based on the LDH and MTT results, two compounds were chosen for HCA of cell viability and proliferation: 3i-1000 to represent the more toxic compounds and 3i-1047 as a non-toxic representative. HiPSCs and hiPSC-CMs were selected for HCA assays as representatives of sensitive and resistant cell types, respectively. To compare HCA-based cell viability analysis with the more conventional MTT assay, cell viability was assessed using a mitochondrion stain (MitoTracker), whose accumulation in mitochondria is dependent on mitochondrial membrane potential. Correspondingly to the MTT results with hiPSCs, while 3i-1047 did not affect MitoTracker staining, 3i-1000 impaired mitochondrial function in hiPSCs at 10 µM concentration, as reflected by a 5.7-fold (*P* = 0.001) increase in the percentage of MitoTracker negative cells as compared to DMSO-treated cells (Supplementary Fig. S3). Accordingly, the average MitoTracker intensity in the perinuclear area reduced substantially in cells exposed to 10 µM 3i-1000 (Supplementary Fig. S3). Of note, the cell death induced by 3i-1000 at 10 µM also reduced hiPSC cell numbers by 98%. Normalized cell densities are shown in Supplementary Figure S3. The compounds had no effect on the proportion of MitoTracker negative hiPSC-CMs, and induced a slight increase in the average MitoTracker intensity in hiPSC-CMs (Supplementary Fig. S3). This increased MitoTracker staining may be due to increased numbers of mitochondria in hiPSC-CMs, which would be in line with the results for 3i-1047 in the MTT assay.

### High-content analysis of hiPSC-CM proliferation

**Fig. 7:**
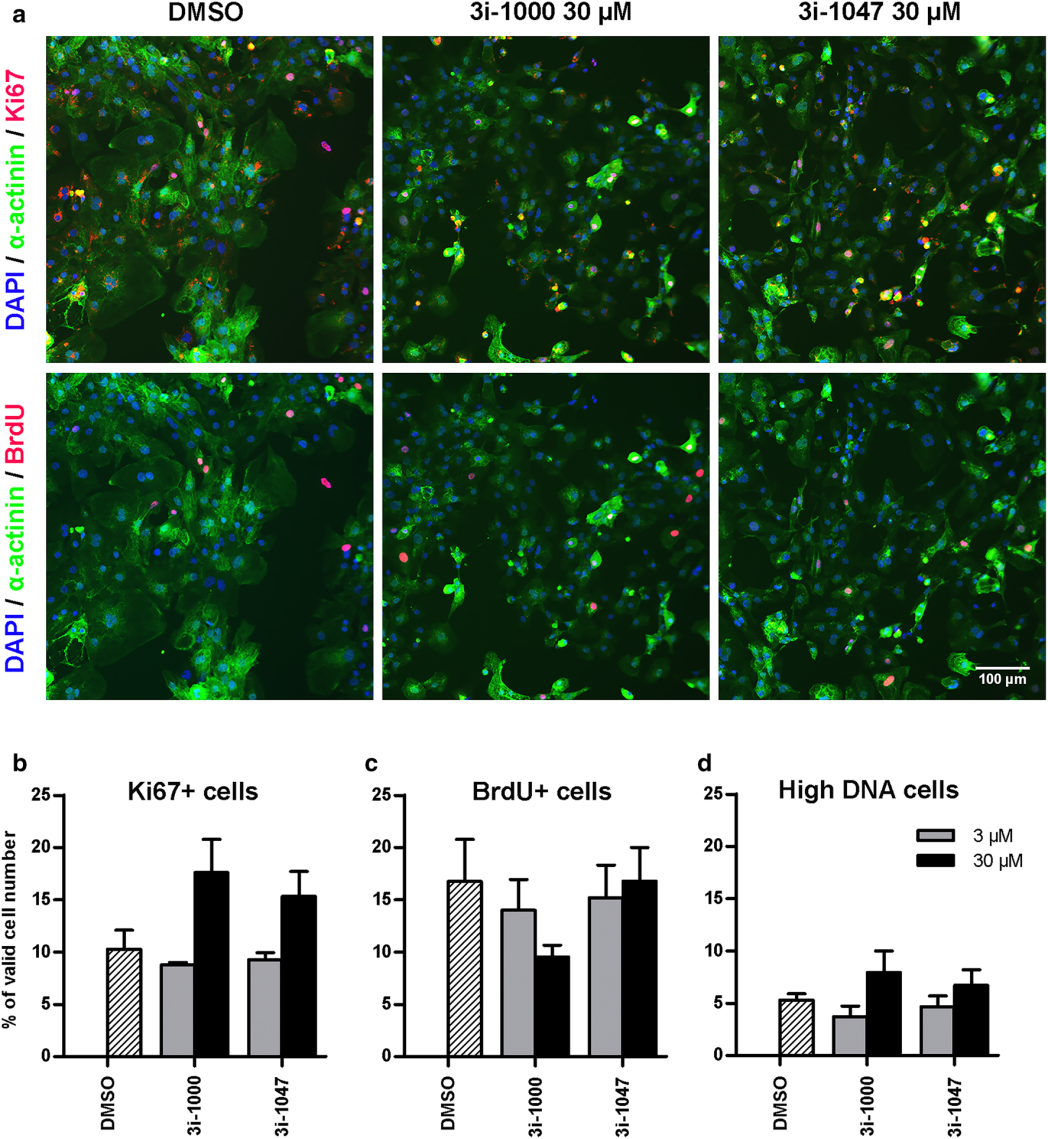
Effects of compounds 3i-1000 and 3i-1047 on cell proliferation in hiPSC-CMs. After a 24-h compound exposure and a concomitant BrdU-loading (24 h), the cells were fixed and stained for α-actinin, DNA (DAPI), Ki67 and BrdU. Imaging and analysis was carried out using CellInsight High Content Analysis Platform. **a** Representative images from four independent experiments are shown. Adjustments of individual color channels to enhance brightness and contrast were made identically to all representative images. The quantifications for the proportion of **b** Ki67-positive cells, **c** BrdU-positive cells, and **d** cells with high DNA staining are expressed as mean + SEM (*n* = 4). No statistically significant differences were observed compared to DMSO (one-way ANOVA followed by Tukey’s HSD)

The effects of 3i-1000 and 3i-1047 on the proliferation of hiPSC-CMs were analysed using Ki67 and BrdU immunostainings and HCA. At the concentration of 30 µM both 3i-1000 and 3i-1047 induced 1.7- and 1.5-fold increases, respectively, in the percentage of Ki67+ cells compared to DMSO-treated cells (Fig. 7), paralleling with substantial increases in the average intensity of nuclear Ki67 staining (Supplementary Fig. S4), indicating that the compounds may promote cell cycle activation. This was, however, not linked to a similar increase in the proportion of BrdU+ hiPSC-CMs. The compound 3i-1047 had no effect on the percentage of BrdU+ cells (Fig. 7) or on the average intensity of nuclear BrdU staining (Supplementary Fig. S4). Interestingly, there was a tendency for 3i-1000 to induce a decrease (43%, *P* = 0.233) in BrdU+ hiPSC-CMs as compared to DMSO (Fig. 7) and in the average intensity of nuclear BrdU staining (Supplementary Fig. S4). Furthermore, neither 3i-1000 nor 3i-1047 caused significant changes in the percentage of cells with high total intensity of DNA staining compared to DMSO (Fig. 7) or in the average total intensity of DNA staining (Supplementary Fig. S4), indicating that the proportion of cells in the G2 phase of the cell cycle was not affected. Taken together, the compounds did not have a consistent inhibitory or promoting effect on hiPSC-CM proliferation.

### High-content analysis of stem cell proliferation

**Fig. 8:**
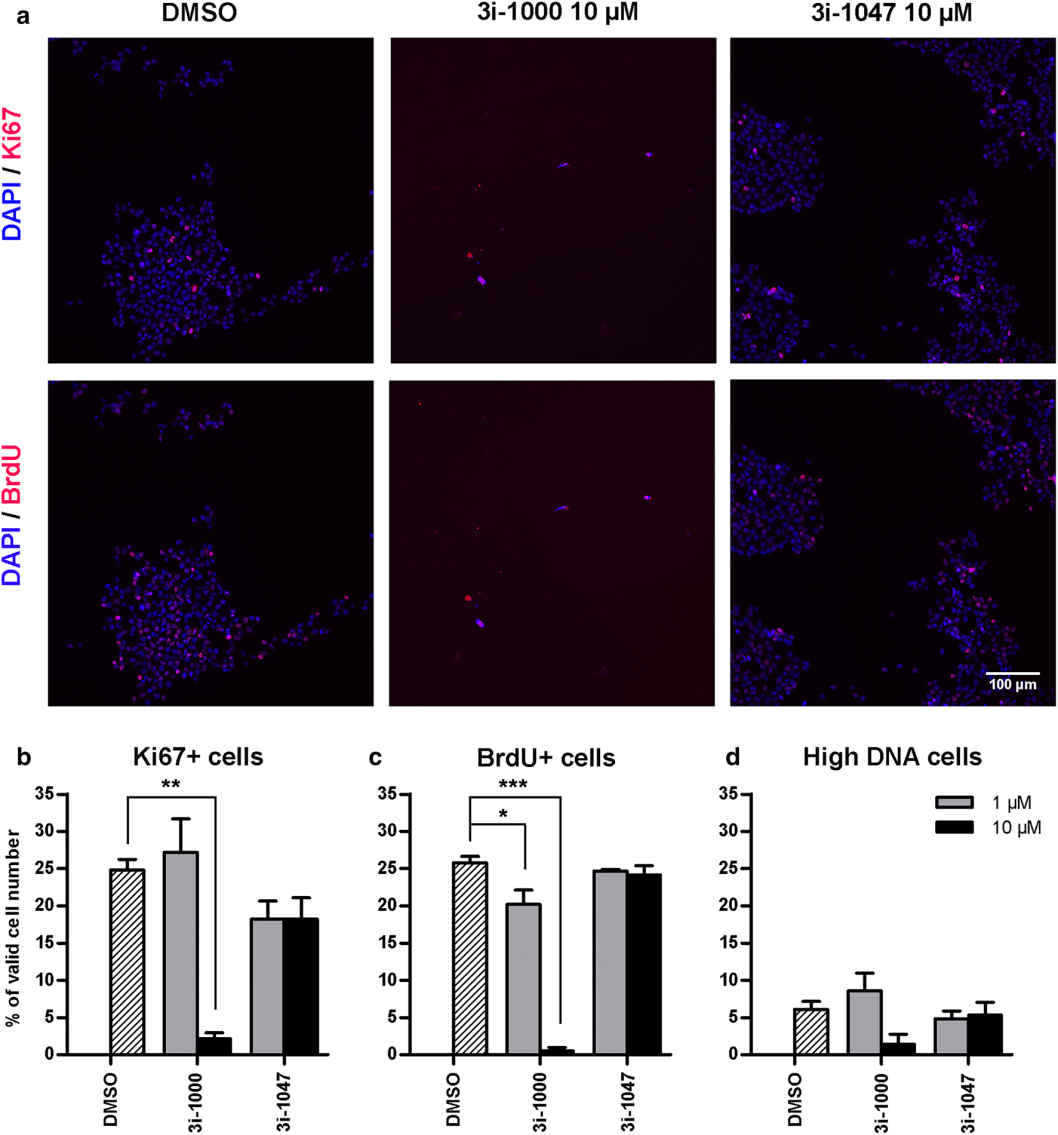
Effects of compounds 3i-1000 and 3i-1047 on cell proliferation in hiPSCs. After a 24-h compound exposure, the cells were loaded with BrdU (1 h) and subsequently fixed and stained for DNA (DAPI), Ki67 and BrdU. Imaging and analysis was carried out using CellInsight High Content Analysis Platform. **a** Representative images from three independent experiments are shown. Adjustments of individual color channels to enhance brightness and contrast were made identically to all representative images. The analysis results for the percentage of **b** Ki67-positive cells, **c** BrdU-positive cells, and **d** cells with high DNA staining are expressed as mean + SEM (*n* = 3). ***P < 0.001 vs. DMSO; **P < 0.01 vs. DMSO; *P < 0.05 vs. DMSO (one-way ANOVA followed by Tukey’s HSD or Welch ANOVA followed by Games–Howell)

In hiPSCs, the compound 3i-1000 decreased the proportion of Ki67 positive (*P* = 0.001) and BrdU-positive (*P* < 0.001) cells significantly at 10 µM, but not at 1 µM (Fig. 8), and this was paralleled by decreased average intensity of nuclear Ki67 and BrdU stainings (Supplementary Fig. S4). The compound also induced a considerable 77% decrease (*P* = 0.062) in cells with high total DNA staining intensity representing cells in G2 phase of the cell cycle (Fig. 8) and a 66% decrease in the average total intensity of DNA staining (Supplementary Fig. S4). In contrast, 3i-1047 (at 1 or 10 µM) had no effect on the percentage of Ki67+, BrdU+ or high DNA cells (Fig. 8) or on the corresponding average staining intensities (Supplementary Fig. S4). Cell numbers were significantly reduced in response to 3i-1000 treatment as shown in Fig. 8a and Supplementary Figure S3.

### The toxic GATA4-targeted compounds induce apoptosis in stem cells

**Fig. 9:**
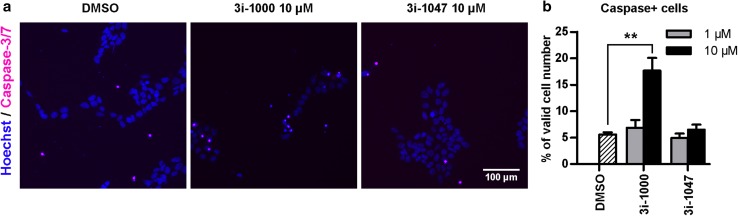
Effect of compounds 3i-1000 and 3i-1047 on caspase activation in hiPSCs. After a 4-h compound exposure, the cells were incubated with a detection reagent for activated Caspase-3/7 for 50 min and the nuclei were stained with Hoechst for 10 min. Fixed or live cells were imaged with CellInsight High Content Analysis Platform. **a** Representative images from four independent experiments are shown. Adjustments of individual color channels to enhance brightness and contrast were made identically to all representative images. **b** Quantification of the proportion of cells positive for fluorescent caspase 3/7 activity reporter is expressed as mean + SEM (*n* = 6). **P < 0.01 vs. DMSO (Welch ANOVA followed by Games–Howell)

Finally, to elucidate the mechanism of cell death caused by the toxic compounds in hiPSCs, caspase-3/7 activation was analysed using HCA. To minimize the cell loss caused by cell death and detachment, a shorter 4-h exposure time was used. The compound 3i-1000 induced a 3.2-fold (± 0.5; SEM) increase in cells positive for the fluorescent caspase reporter (*P* = 0.009), whereas 3i-1047 had no effect on caspase-3/7 activity (Fig. 9). The findings are in agreement with the MTT and LDH test results, which showed no signs of necrosis, yet significant reductions in cell viability after 24 h exposure to 3i-1000, and thus suggest the caspase-dependent apoptosis as a mechanism contributing to stem cell death caused by the toxic GATA4-targeted compounds.

## Discussion

Toxicity is a major cause of failure in drug development projects (Kola and Landis 2004). In general, toxic compounds are identified already during lead compound screening; however, sometimes they only come up in clinical trials. As the monetary cost of failure increases steeply with progression towards clinical trials (Paul et al. 2010), more efficient toxicity screening in early phases could improve research and development productivity noticeably. Considering the preclinical phase of drug development, the use of more precise cell models for cytotoxicity screening prior to in vivo testing would not only improve the validity of the in vivo efficacy data, but also would reduce the use of experimental animals. Therefore, in this study we sought to compare different cardiac and stem cell types of rodent and human origin for cytotoxicity screening. Additionally, our aim was to characterize the in vitro cytotoxicity of a selected set of novel cardiac transcription factor-targeted compounds to identify toxicity-related molecular structures.

According to our previous study, the lead compound 3i-1000 exhibited no toxicity in NRVCs at concentrations up to 50 µM (Välimäki et al. 2017). Here we investigated in detail the safety of the compound and its derivatives by carrying out cytotoxicity screening in eight different cell types. The northern part of the 3i-1000 family of compounds exhibits a major contribution to inhibition of GATA4–NKX2-5 synergy (Välimäki et al. 2017). Furthermore, our preliminary data indicated that modifications in the southern part may affect the toxicity of the compounds in some cell types. Compounds for the study were, therefore, preselected to uncover and identify toxicity-related molecular properties especially concerning the modifications in the southern part whereas diversity in the northern part was not preferred. Using suitably functionalized northern and southern fragments, structural variations were easily introduced in different regions of molecules, fitting to the general structure of GATA4-targeted compounds.

In line with our previous findings, 3i-1000 and its derivatives were non-toxic to cardiomyocytes, fibroblasts and H9c2 cardiac myoblasts. The comparison of NRVCs and hiPSC-CMs was of particular interest, as the use of stem cell-derived cardiomyocytes has grown exponentially over the past couple of years upon improved differentiation and purification techniques. There is intensive discussion on the applicability of hiPSC-CMs for drug development research and cardiotoxicity testing due to their immature phenotype, which is linked to differences in metabolism, sarcomere organization, gene expression and G protein-coupled receptor (GPCR) signalling (Földes et al. 2014; Magdy et al. 2018). In the present study, the GATA4-targeted compounds were non-toxic to both cardiomyocyte types, suggesting that they do not exhibit species-specific cardiotoxicity and support the use of hiPSC-CMs instead of primary cells in early toxicity screening to reduce the use of experimental animals.

Conversely, the screening in stem cells and stem cell-derived progenitor cells revealed that some of the compounds exhibit potent and perhaps unexpected toxicity resulting in apoptosis. Stem cells are known to be highly sensitive to rapid apoptosis in response to DNA damage (Dumitru et al. 2012; Liu et al. 2013, 2014). Multiple mechanisms have been proposed to contribute to the hypersensitivity, including tumor suppressor protein p53 signalling, short duration of cell cycle, as well as mitochondrial priming (e.g., disparities in the expression of pro-apoptotic and anti-apoptotic proteins, and differences in mitochondrial structure and activity), but the exact mechanisms are still unknown. The compound-induced toxicity was selective to stem cells and progenitor cells, as it was not observed in other proliferating cells such as H9c2 cells, MEFs or CFs.

The structure–toxicity analysis reveals a structurally coherent set of GATA4-targeted compounds with a common nominator, a phenyl ring in the southern part, as a major cause for the observed progenitor- and stem cell-specific toxicity. Detailed structural analysis of toxic compounds reveals the uniform appearance of low energy conformations and consistent stereochemistry of the ring system in southern part defined by force field- and knowledge-based methods. Based on the present results, the characteristic conformational feature that distinguishes the toxic and non-toxic compound classes is the dihedral angle θ between the two aromatic planes in the southern part. Moreover, the effect of the small molecules on GATA4–NKX2-5 transcriptional synergy in luciferase reporter assay did not predict the cellular toxicity; for example, compound 3i-1229 is an example of a toxic compound with no inhibitory effect on GATA4–NKX2-5 interaction. Based on our previously reported protein kinase and GPCR profiling results (Välimäki et al. 2017) we also find it unlikely that a direct effect on protein kinases or GPCRs would contribute to the observed stem cell toxicity. Nevertheless, based on the current results, further lead optimization can be directed towards the non-toxic derivatives, i.e., compounds with a five-membered ring in the southern part of the molecule.

In summary, by screening novel GATA4-targeted small-molecular compounds for cytotoxicity in several cell types, we revealed profound differences in the sensitivity of different cell types, implicating that the model for toxicity screening should be chosen carefully. Furthermore, the discovery that small changes in the southern part of the molecular structure of GATA4-targeting compounds contribute to the toxicity in stem cells and progenitor cells allows further drug development using the non-toxic derivatives as a starting point. We propose that toxicity screening using stem cells in early phases of drug discovery projects can be useful in reducing the risk of toxicity-dependent failure at later stages of drug development, particularly when targeting transcription factors.

## Electronic supplementary material

Below is the link to the electronic supplementary material.

Supplementary material 1 (PDF 3951 KB)

## Data Availability

The datasets generated during and analysed during the current study are available from the corresponding author on reasonable request.
