# Supplementary material for: Stem cells are the most sensitive screening tool to identify toxicity of GATA4-targeted novel small-molecule compounds
Source: Arch Toxicol. 2018 Jul 9;92(9):2897–911. doi: 10.1007/s00204-018-2257-1 (PMC6132687; doi:10.1007/s00204-018-2257-1)
Supplement: Supplementary file 1 — Supplementary material 1 (PDF 3951 KB) [file 204_2018_2257_MOESM1_ESM.pdf]

Supplementary Information

Stem cells are the most sensitive screening tool to identify toxicity of GATA4-targeted novel small-molecule compounds

Archives of Toxicology

S. Tuuli Karhu, Mika J. Välimäki, Mikael Jumppanen, Sini M. Kinnunen, Lotta Pohjolainen, Robert S. Leigh, Samuli Auno, Gábor Földes, Gustav Boije af Gennäs, Jari Yli-Kauhaluoma, Heikki Ruskoaho, Virpi Talman*

*Corresponding author: Division of Pharmacology and Pharmacotherapy, Faculty of Pharmacy, University of Helsinki, P.O. Box 56, FI-00014 Helsinki, FINLAND. E-mail: virpi.talman@helsinki.fi, tel. +358 50 448 0768

Contents:

Supplementary Figures S1 – S4, pages 2-5
Supplementary Table S1, page 6
Supplementary Methods, pages 6-28

- Cell culture and differentiation, pages 6-8
- Syntheses and characterization of compounds, pages 7-13
- ^{1}H NMR and ^{13}C spectra for the compounds, pages 14-28
- References, page 29

Supplementary Figures

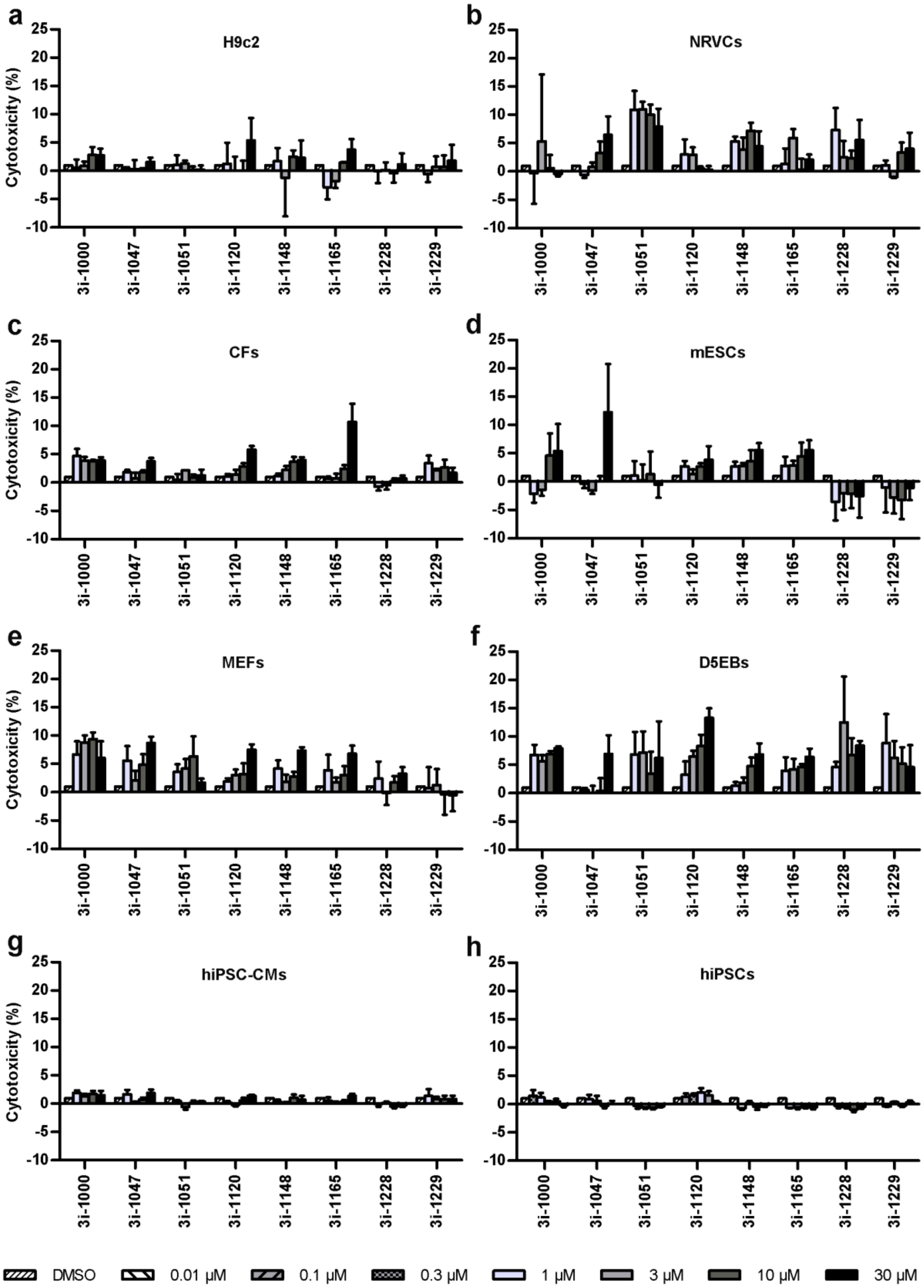

Supplementary Fig. S1 Necrotic cell death in different cell types after 24 h exposure to the test compounds, measured by LDH assay. Results are expressed as mean + SEM (n = 3–4). NRVCs, primary neonatal rat ventricular cardiomyocytes; CFs, primary neonatal rat cardiac fibroblasts; mESCs, mouse embryonic stem cells; MEFs, mouse embryonic fibroblasts; D5EBs, mESC-derivates from day 5 embryoid bodies; hiPSCs, human induced pluripotent stem cells; hiPSC-CMs, hiPSC-derived cardiomyocytes

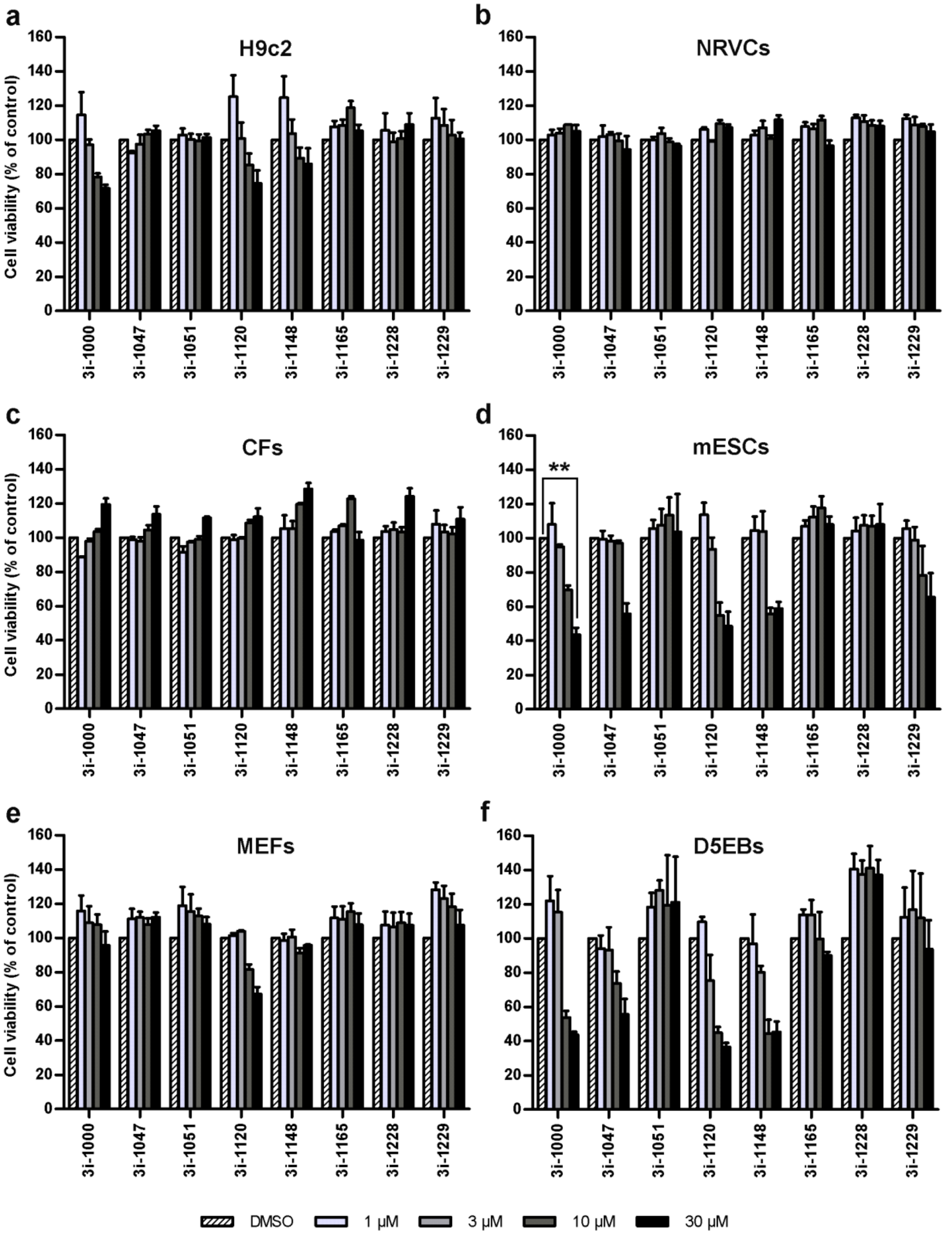

Supplementary Fig. S2 Cell viability in different cell types after 24 h exposure to the test compounds, measured by MTT assay. Results are expressed as mean + SEM (n = 3–4). **, P < 0.01 vs. DMSO (One-way ANOVA and Tukey's HSD). NRVCs, primary neonatal rat ventricular cardiomyocytes; CFs, primary neonatal rat cardiac fibroblasts; mESCs, mouse embryonic stem cells; MEFs, mouse embryonic fibroblasts; D5EB, mESC-derivates from day 5 embryoid bodies

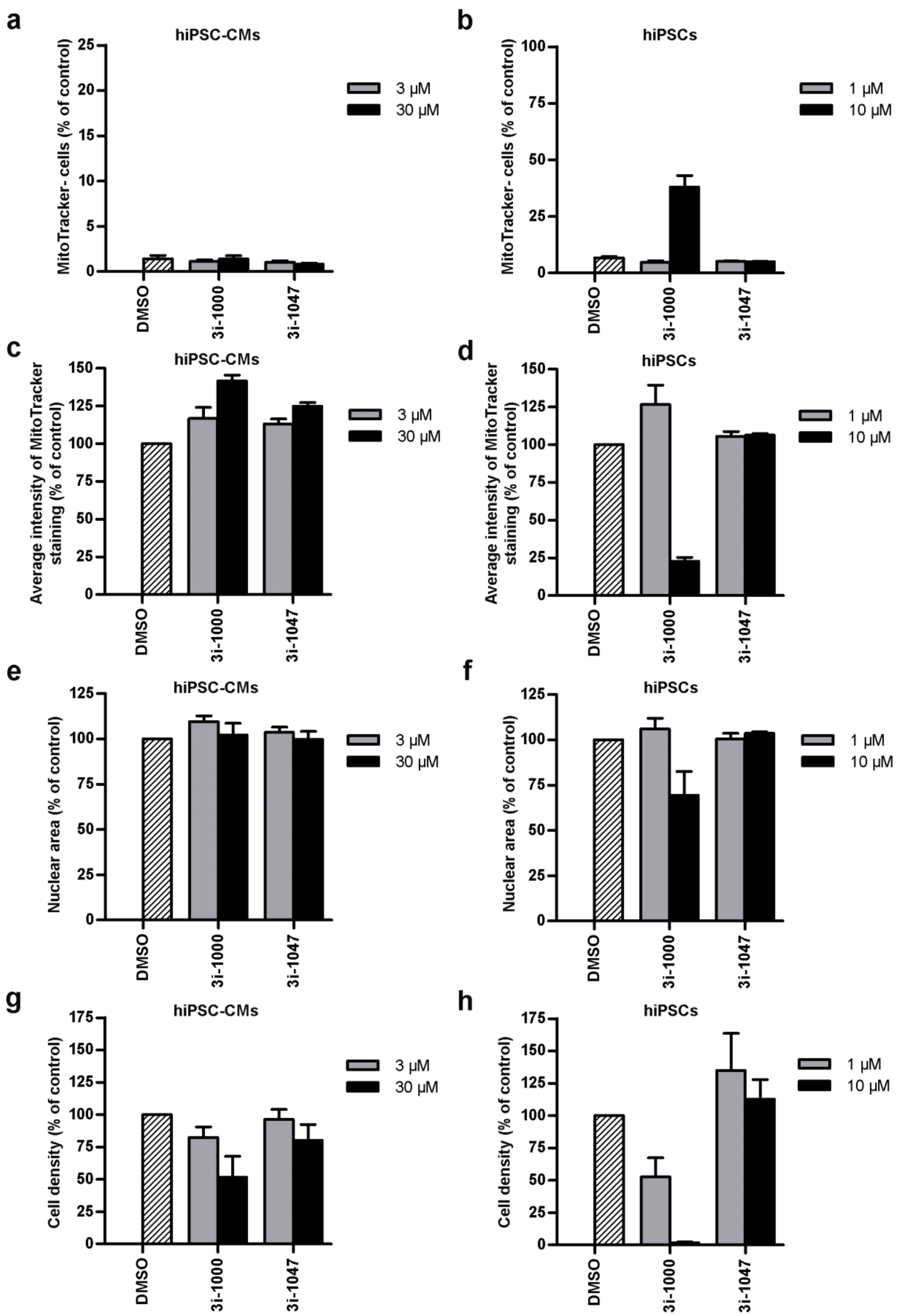

Supplementary Fig. S3 Effects of the compounds 3i-1000 and 3i-1047 on cell viability in hiPSC-CMs (a, c, e, g) and hiPSCs (b, d, f, h) after a 24-h exposure. The high content analysis results are expressed as mean + SEM (n = 3–4). hiPSCs, human induced pluripotent stem cells; hiPSC-CMs, hiPSC-derived cardiomyocytes

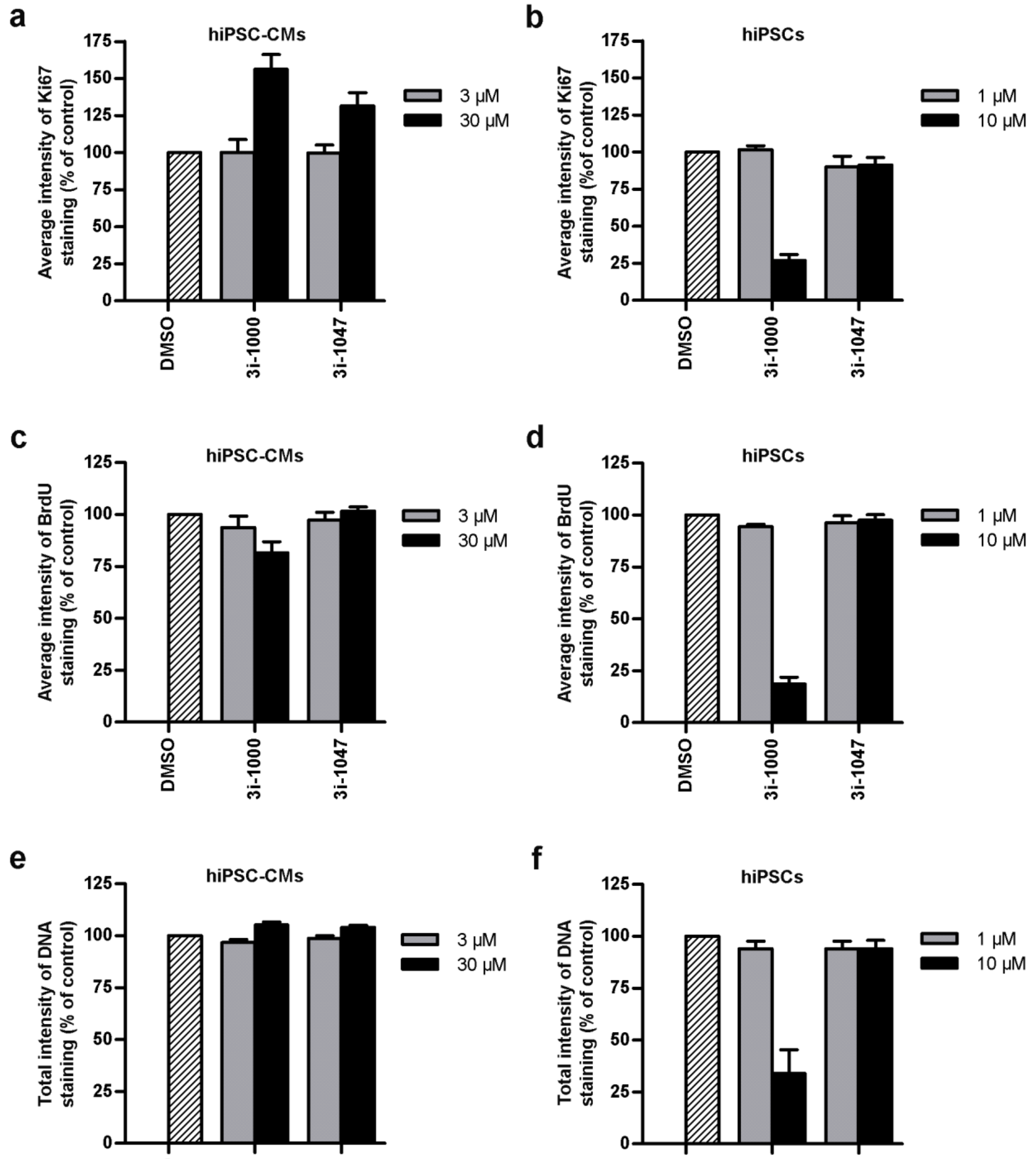

Supplementary Fig. S4 Effects of the compounds 3i-1000 and 3i-1047 on proliferation in hiPSC-CMs (a, c, e) and hiPSCs (b, d, f) after a 24-h exposure. The high-content analysis results are expressed as mean + SEM (n = 3–4). hiPSCs, human induced pluripotent stem cells; hiPSC-CMs, hiPSC-derived cardiomyocytes

Supplementary Tables

Supplementary Table S1 Summary of the compound conformation calculations showing the torsion angles for ring system of low energy conformations and the number of compound conformations generated during the search. ND = Not determined

Compounds	MMFF94X		OPLS-AA	
	**Torsional angle (degrees)**	**No. of conformations**	**Torsional angle (degrees)**	**No. of conformations**
**3i-1000**	49.0	46	42.1	149
**3i-1047**	13.4	56	19.4	308
**3i-1051**	ND	ND	ND	ND
**3i-1120**	41.9	309	42.6	488
**3i-1148**	43.1	39	50.5	91
**3i-1165**	11.3	22	14.6	66
**3i-1228**	0.1	13	7.4	263
**3i-1229**	50.1	32	27.9	26

Supplementary Methods

Cell culture and differentiation

COS-1 and H9c2 cells were cultured in Dulbecco's modified Eagle medium (DMEM) supplemented with 10% fetal bovine serum (FBS), 100 U/ml of penicillin, and 100 µg/ml of streptomycin. H9c2 cells were seeded at 5,000 cells/well in 96-well plates and COS-1 cells at 14,000 cells/well in Isoplate-96 microplates (PerkinElmer, Turku, Finland). Both cell types were grown overnight in standard conditions prior to experiments.

Primary cultures of rat neonatal ventricular cardiomyocytes and fibroblasts were prepared from 1–3 day old Wister rats as described earlier (Tölli et al. 2014). Animals were sacrificed by decapitation and ventricles were separated and cut into small pieces. Tissue pieces were enzymatically digested by incubating them in a solution containing 100 mM NaCl, 10 mM KCl, 1.2 mM KH_2PO_4, 4.0 mM $MgSO_4$, 50 mM taurine, 20 mM glucose, 10 mM 4-(2-hydroxyethyl)-1-piperazineethanesulfonic acid (HEPES), 2 mg/ml collagenase type 2, 2 mg/ml pancreatin, 100 U/ml of penicillin, and 100 µg/ml of streptomycin, for 1–2 h at 37 °C under 600 rpm shaking conditions. The cells in suspension were collected and centrifuged for 5 min at 160 g. The supernatant and the top layer of the pellet were discarded and the isolated cardiac cells were resuspended in DMEM/F12 supplemented with 10% FBS, 100 U/ml of penicillin, and 100 µg/ml of streptomycin. Cardiomyocytes and non-myocytes (fibroblasts) were separated by plating them onto cell culture flasks and incubating 45–60 min at 37 °C in standard conditions. Unattached cells (enriched cardiomyocytes) were collected with the medium and seeded at 40,000 cells/well in 96-well plates. After 24 h the FBS-supplemented medium was changed to complete serum free medium (CSFM; DMEM/F-12 supplemented with 2.5 mg/ml bovine serum albumin, 5 µg/ml insulin, 5 µg/ml transferrin, 5 ng/ml selenium, 2.8 mM sodium pyruvate, 0.1 nM triiodo-L-thyronine (T_3), 100 U/ml of penicillin, and 100 µg/ml of streptomycin) for 24 h prior to drug treatments, which were carried out in CSFM. The attached cell fraction from pre-plating was considered fibroblasts (Polinger 1970), which were grown in DMEM/F-12 medium supplemented with FBS and antibiotics as described above for 3 days and then dissociated with trypsin-EDTA and seeded at 8,000 cells/well in 96-well plates after which they were allowed to grow for another 3 days prior to drug treatments.

Mouse embryonic stem cells (mESCs), embryoid bodies (EBs), and primary mouse embryonic fibroblasts (MEFs) were used for toxicity experiments. E14 mESCs were cultured in feeder-free conditions on plates coated with 0.1% gelatin in DMEM with 15% FBS, GlutaMax, MEM non-essential amino acids, 2-mercaptoethanol, and leukemia inhibitory factor (LIF). For toxicity assays, the cells were dissociated with TrypLe and plated at 5,000 cells/well in gelatin-coated 96-well plates 24 hours prior to compound exposures. To induce differentiation of mESCs, cells were plated on day 0 in 20 µl hanging drops at 25,000 cells/ml in inverted V-bottom plates in DMEM with 20% FBS and with all above supplements except LIF. On day 2 medium was added to EBs, and the EBs were dissociated with TrypLe and plated at 5,000 cells/well in gelatin-coated 96-well plates on day 5 and exposed to test compounds 24 later. Primary MEFs were cultured on 0.1% gelatin-coated plates in DMEM with 10% FBS and the above supplements except LIF, dissociated with TrypLe and plated at 5,000 cells/well in gelatin-coated 96-well plates 24 h prior to compound exposures.

Human pluripotent stem cells (hiPSCs): The iPS(IMR90)-4 line (Yu et al. 2007) was purchased from WiCell (Madison, Wisconsin, USA). The cells were cultured in Essential 8 ™ medium (E8) on 6-well plates coated with Matrigel® (1:50) and passaged 1:15 approximately every four days. The cells were dissociated using Versene®, resuspended in E8 containing 10 µM ROCK inhibitor (Y-27632) and seeded at 10,000 cells/well (cytotoxicity assays) or 5,000 cells/well (high-content imaging) in Matrigel®-coated 96-well plates. The cells were incubated overnight in standard conditions prior to compound addition.

Cardiomyocyte differentiation from hiPSCs: Human iPSC-derived cardiomyocytes (hiPSC-CMs) were produced from the IMR90 hiPSC line using the well-established small molecule induction (Burridge et al. 2014). The hiPSCs were grown on 6-well plates in E8 medium until they were 80–95% confluent. Differentiation towards CM lineage was initiated by adding 6 µM CHIR99021 (day 0) in RPMI 1640 medium supplemented with B-27 without insulin (RB-ins) to the cells. CHIR9921 was removed and replaced with fresh RB-ins after 24 h. At day 3 the medium was changed to RB-ins containing 2.5 µM C59 for 48 h. On days 5, 7 and 9 the cells were fed with RB-ins and beating cardiomyocytes were generally observed from days 7–8 onwards. From day 11 to day 15 the cells were maintained in RPMI 1640 without glucose with B-27 supplement in order to purify cardiomyocytes. From day 15 onwards the cells were maintained in RPMI 1640 supplemented with B-27 (RB+ins). Beating hiPSC-CMs were dissociated between days 15 and 22 by incubating them in cell dissociation solution containing 40% enzyme-free cell dissociation buffer, 40% RPMI 1640 and 20% trypsin-EDTA (final trypsin concentration 0.01%) for 7–8 min, whereafter the cells were collected and trypsin was inactivated in RB+ins supplemented with 10% FBS. After centrifugation the cells were suspended in RB+ins with 10% FBS containing 10 µM ROCK inhibitor and seeded at 15,000 cells/well (cytotoxicity assays) or 20,000 cells/well (high content analysis) in gelatin-coated 96-well plates. The cells were let to attach for 2 days at 37 °C in standard conditions, whereafter the compounds were added in RB+ins. In general, the differentiation yielded an almost pure (>95%) cardiomyocyte culture.

Syntheses and characterization of compounds

Materials and general procedures. All reagents were acquired from Sigma-Aldrich (Schnelldorf, Germany) and Enamine (Kiev, Ukraine), and were used without further purification. All reactions in anhydrous solvents were conducted in oven-dried glassware under anhydrous argon. Thin-layer chromatography (TLC) was performed using Silica Gel 60 F254 (Merck) and Silica Gel 60 NH_2 F254s aluminium sheets (Merck), visualized by UV illumination and stained with ninhydrin in EtOH (1.5% w/v). Microwave reactions were performed with a Biotage Initiator$^+$ Robot Eight microwave (Uppsala, Sweden). Column chromatography was performed with an automated high performance flash chromatography Biotage Sp4-system or with a Isolera Spektra One-system (Uppsala, Sweden) using a 0.1-mm path length flow cell UV-detector/recorder module (fixed wavelength 254 nm) for the Sp1-system or a variable UV-VIS (200-800 nm) photodiode array detector for the Isolera Spektra One-system, and the indicated mobile phase. The ^{1}H and ^{13}C NMR spectra were recorded on a Varian

Mercury-VX 300 (Agilent Technologies, Santa Clara, California, USA) or a Bruker Ascend 400 – Avance III HD NMR spectrometer (Bruker Corporation, Billerica, MA, USA) spectrometer as solutions in $CDCl_3$ and DMSO-d_6. Chemical shifts (δ) are reported as parts per million (ppm) relative to the solvent peak ($CDCl_3$ 7.26 and 77.16 ppm, DMSO-d_6 2.50 and 39.52 ppm). Multiplicities of peaks are represented by s (singlet), d (doublet), t (triplet), q (quartet), quintet (qn), and m (multiplet). Exact mass and purity (>95%) of all tested compounds was confirmed by LC-MS analyses with a Waters Acquity® UPLC system (Waters, Milford, MA, USA) equipped with an Acquity UPLC® BEH C18 column (1.7 µm, 50 x 2.1 mm, Waters, Ireland), an Acquity PDA detector and a Waters Synapt G2 HDMS mass spectrometer (Waters, Milford, MA, USA) via an ESI ion source in positive mode. High resolution mass (HRMS-ESI) data was reported for the molecular ions [M+H]+.

[3-Amino-5-(4-methoxyphenyl)-1*H*-pyrazol-1-yl](5-methyl-3-phenylisoxazol-4-yl)methanone (1a, 3i-1229)

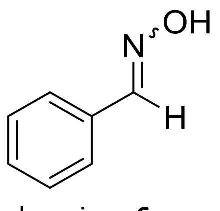

i. (E/Z)-Benzaldehyde oxime (**6a**): To a solution of benzaldehyde **4a** (1.92 ml, 18.8 mmol) in EtOH (40 ml) was added hydroxylammonium chloride (1.44 g, 20.7 mmol, 1.1 equiv) and pyridine (1.64 g, 20.7 mmol, 1.1 equiv). The reaction mixture was stirred at rt for 5 h. The reaction was quenched by addition of a saturated solution of NH_4Cl in H_2O (25 ml), the resulting mixture was extracted with DCM (3 × 25 ml) and the combined organic phases were dried with anhydrous Na_2SO_4. The solvent was removed *in vacuo* to give (*E/Z*)-benzaldehyde oxime **6a** as a colorless oil, which was used in the next reaction without further purification (1.68 g). ^{1}H NMR (300 MHz, $CDCl_3$) δ_{ppm} 8.36 (br s, 1H), 8.16 (s, 1H), 8.03–7.89 (m, 3H), 7.68–7.54 (m, 2H), 7.50–7.38 (m, 3H). ^{13}C NMR (75 MHz, $CDCl_3$) δ_{ppm} 150.5, 132.1, 130.2, 128.9, 127.2.

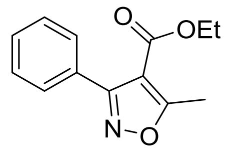

ii. Ethyl 5-methyl-3-phenylisoxazole-4-carboxylate (**7a**): To a solution of (*E/Z*)-benzaldehyde oxime **6a** (546 µl, 5.00 mmol), ethyl 2-butynoate **5a** (1.46 ml, 12.5 mmol, 2.5 equiv) and KCl (373 mg, 5.00 mmol) in H_2O (30 ml) was added Oxone® (2.30 g, 7.50 mmol, 1.5 equiv). The reaction mixture was stirred at rt for 4 h. The resulting mixture was extracted with DCM (3 × 20 ml), the combined organic phases were dried with anhydrous Na_2SO_4. The solvent was removed *in vacuo* and the crude product mixture was subjected to a purification by an automated high performance flash chromatography system (*n*-hexane/EtOAc 0→20%) to yield ethyl 5-methyl-3-phenylisoxazole-4-carboxylate **7a** as a colorless oil. (554 mg, 48 %). ^{1}H NMR (300 MHz, $CDCl_3$) δ_{ppm} 7.70–7.57 (m, 2H), 7.51–7.38 (m, 3H), 4.24 (q, J = 7.1 Hz, 2H), 2.73 (s, 3H), 1.22 (t, J = 7.1 Hz, 3H). ^{13}C NMR (75 MHz, $CDCl_3$) δ_{ppm} 175.9, 162.7, 162.1, 129.8, 129.5, 128.7, 128.1, 108.6, 60.8, 14.1, 13.7.

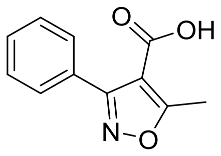

iii. 5-Methyl-3-phenylisoxazole-4-carboxylic acid (**2a**): To a solution of ethyl 5-methyl-3-phenylisoxazole-4-carboxylate **7a** (5.2 mg, 0.020 mmol) in an equimixture of MeOH and H_2O (60 ml) was added a 50 wt % solution of NaOH in H_2O (250 µl, 4.8 mmol, 2 equiv). The reaction mixture was stirred at 60 °C for 24 h and methanol was evaporated *in vacuo*. The remaining aqueous phase was acidified to pH 1 with a 1 M solution of HCl in H_2O. The precipitated product was extracted from the water phase with DCM (3 × 25 ml). The combined organic phases were washed with brine (25 ml), dried with anhydrous Na_2SO_4, filtered, and evaporated *in vacuo* to yield 5-methyl-3-phenylisoxazole-4-carboxylic acid **2a** as a white solid (470 mg, 96%). ^{1}H NMR (300 MHz, DMSO-d_6) δ_{ppm} 13.04 (br s, 1H), 7.66–7.55 (m, 2H), 7.52–7.43 (m, 3H), 2.69 (s, 3H). ^{13}C NMR (75 MHz, DMSO-d_6) δ_{ppm} 175.5, 162.6, 162.1, 129.6, 129.1, 128.4, 128.1, 108.6, 13.1.

iv. [3-Amino-5-(4-methoxyphenyl)-1H-pyrazol-1-yl](5-methyl-3-phenylisoxazol-4-yl)methanone (**1a, 3i-1229**): To a solution of 5-methyl-3-phenylisoxazole-4-carboxylic acid **2a** (0.050 g, 0.25 mmol) in dry DMF (5 ml) was added 3-amino-5-(4-methoxyphenyl)pyrazole (0.047 g, 0.25 mmol), HBTU (0.187 g, 0.49 mmol, 2 equiv), and DIPEA (56 µl, 0.32 mmol, 1.3 equiv). The reaction mixture was stirred at rt for 18 h. Diethyl ether (20 ml) was added, and the organic phase was washed with water (2 × 15 ml) and brine (20 ml). The organic phase was dried with anhydrous Na_2SO_4, filtered and evaporated *in vacuo*. The crude product mixture was subjected to a purification by an automated high performance flash chromatography system (*n*-hexane/EtOAc 0→50%) to yield [3-amino-5-(4-methoxyphenyl)-1*H*-pyrazol-1-yl](5-methyl-3-phenylisoxazol-4-yl)methanone **1a** (45 mg, 49%). ^{1}H NMR (400 MHz, DMSO-*d*$_6$) δ_{ppm} 7.54–7.47 (m, 2H), 7.42 (d, *J* = 8.9 Hz, 2H), 7.41–7.33 (m, 3H), 6.88 (d, *J* = 8.8 Hz, 1H), 6.75 (br s, 2H), 5.70 (s, 1H), 3.75 (s, 3H), 2.59 (s, 3H). ^{13}C NMR (101 MHz, DMSO-*d*$_6$) δ_{ppm} 172.9, 163.3, 161.5, 159.9, 154.1, 152.1, 129.8, 128.7, 127.4, 127.3, 124.2, 113.8, 111.1, 84.8, 55.1, 12.6. HRMS calcd. for $C_{21}H_{19}N_4O_3$ [M+H]$^+$: 375.1457, found 375.147.

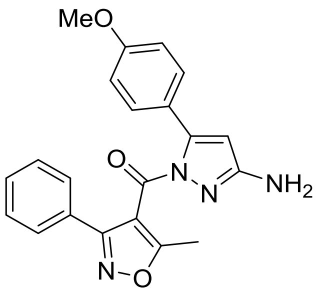

***N*-[4-(Diethylamino)phenyl]-5-methyl-3-(2-methyloxazol-4-yl)isoxazole-4-carboxamide (1b, 3i-1228)**

i. (E/Z)-2-Methyloxazole-4-carbaldehyde oxime (**6b**)*:* To a solution of 2-methyloxazole-4-carbaldehyde **4b** (0.10 g, 0.90 mmol) in MeOH (5 ml) was added hydroxylamine hydrochloride (69 mg, 0.99 mmol, 1.1 equiv) and sodium acetate (0.10 g, 1.3 mmol, 1.4 equiv). After stirring the reaction mixture at rt for 2 h it was quenched with a saturated aqueous solution of NH_4Cl (10 ml). The aqueous phase was extracted with EtOAc (3 × 20 ml). The combined organic phases were dried with anhydrous Na_2SO_4, filtered and evaporated *in vacuo* to yield (*E/Z*)-2-methyloxazole-4-carbaldehyde oxime **6b** (103 mg, 90%). ^{1}H NMR (300 MHz, acetone-*d*$_6$) δ_{ppm} 11.14 (br s, 1H, minor), 10.34 (br s, 1H, major), 8.44 (d, *J* = 0.5 Hz, 1H, minor), 8.00 (d, *J* = 0.5 Hz, 1H, major), 7.97 (d, *J* = 0.6 Hz, 1H, major), 7.38 (d, *J* = 0.5 Hz, 1H, minor), 2.43 (s, 3H, minor), 2.41 (s, 3H, major). ^{13}C NMR (75 MHz, acetone-*d*$_6$) δ_{ppm} 162.8, 161.5, 143.4, 141.5, 140.0, 138.5, 138.3, 136.2, 13.6, 13.4.

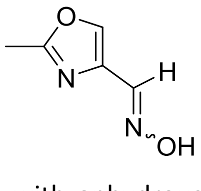

ii. Ethyl 5-methyl-3-(2-methyloxazol-4-yl)isoxazole-4-carboxylate (**7b**)*:* [Hydroxy(tosyloxy)iodo]benzene (240 mg, 0.61 mmol, 1.1 equiv) was added in small portions to a stirred solution of (*E/Z*)-2-methyloxazole-4-carbaldehyde oxime **6b** (71 mg, 0.56 mmol) and ethyl 2-butynoate **5** (78 µl, 0.67 mmol, 1.2 equiv) in H_2O (1 ml). The resulting mixture was stirred at rt for 2 h. The reaction mixture was quenched with a saturated aqueous solution of $NaHCO_3$ (5 ml), and the aqueous phase was extracted with EtOAc (3 × 20 ml). The combined organic phases were dried with anhydrous Na_2SO_4, filtered, and evaporated *in vacuo*. The crude product mixture was subjected to a purification by an automated high performance flash chromatography system (*n*-hexane/EtOAc 0→100%) to yield ethyl 5-methyl-3-(2-methyloxazol-4-yl)isoxazole-4-carboxylate **7b** (26 mg). ^{1}H NMR (300 MHz, $CDCl_3$) δ_{ppm} 9.15, 8.52 (s, 1H), 4.35 (q, *J* = 7.1 Hz, 2H), 2.71 (d, *J* = 0.5 Hz, 3H), 2.62–2.56 (m, 2H), 2.52 (s, 4H), 1.38 (td, *J* = 7.1, 0.4 Hz, 3H). This not completely pure product was used in the next step without further purification.

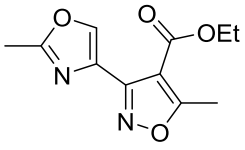

iii. 5-Methyl-3-(2-methyloxazol-4-yl)isoxazole-4-carboxylic acid (**2b**)*:* Sodium hydroxide (8 mg, 0.2 mmol, 2 equiv) was added to a solution of ethyl 5-methyl-3-(2-methyloxazol-4-yl)isoxazole-4-carboxylate **7b** (24 mg, 0.10 mmol) in an equimixture of MeOH and H_2O (3.5 ml). The resulting mixture was stirred at 60 °C for 20 h, and then most of MeOH was removed *in vacuo*. The aqueous layer was acidified with a 1 M solution of HCl in H_2O to pH 1, and subsequently extracted with EtOAc (3 × 20 ml). The combined organic phases were washed with brine (20 ml), dried with anhydrous Na_2SO_4,

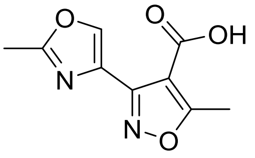

filtered and evaporated *in vacuo* to give 5-methyl-3-(2-methyloxazol-4-yl)isoxazole-4-carboxylic acid **2b** (21 mg). This not completely pure product was used in the next step without further purification.

iv. N-[4-(Diethylamino)phenyl]-5-methyl-3-(2-methyloxazol-4-yl)isoxazole-4-carboxamide (**1b**)*: N,N*-Diethyl-*p*-phenylenediamine **3b** (17 µl, 0.10 mmol), HBTU (76 mg, 0.20 mmol, 2.0 equiv), and DIPEA (23 µl, 0.13 mmol, 1.3 equiv) were added to a solution of 5-methyl-3-(2-methyloxazol-4-yl)isoxazole-4-carboxylic acid **2b** (21 mg, 0.10 mmol) in dry DMF (2 ml). The reaction mixture was stirred at rt for 16 h. Diethyl ether (20 ml) was added, and the organic phase was washed with water (2 × 10 ml). The organic phase was dried with anhydrous Na_2SO_4, filtered and evaporated *in vacuo*. The crude product mixture was subjected to a purification by an automated high performance flash chromatography system (*n*-hexane/EtOAc 0→100%) to yield *N*-[4-(diethylamino)phenyl]-5-methyl-3-(2-methyloxazol-4-yl)isoxazole-4-carboxamide **1b** (0.010 g, 48%). M.p. 133-135 °C. ^1H NMR (300 MHz, $CDCl_3$) δ_{ppm} 11.58 (s, 1H), 8.15 (s, 1H), 7.55–7.49 (m, 2H), 6.74–6.64 (m, 2H), 3.34 (q, *J* = 7.1 Hz, 4H), 2.84 (s, 3H), 2.63 (s, 6H), 1.15 (t, *J* = 7.1 Hz, 6H). ^{13}C NMR (75 MHz, $CDCl_3$) δ_{ppm} 177.0, 162.0, 159.0, 151.0, 145.2, 138.6, 130.6, 127.8, 122.0, 112.7, 111.7, 44.8, 14.2, 14.0, 12.7. HRMS calcd. for $C_{19}H_{22}N_4O_3$ $[M+H]^+$: 355.1770, found 355.1770.

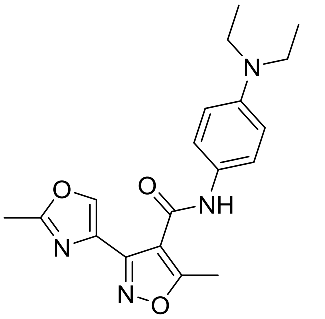

_N_-[4-(Diethylamino)phenyl]-3',5-dimethyl-[3,5'-biisoxazole]-4'-carboxamide (1c, 3i-1047)

3-Methyl-5-(5-methylisoxazol-3-yl)isoxazole-4-carboxylic acid **2c** (48.2 mg, 0.232 mmol), *N,N*-diethyl-*p*-phenylenediamine **3b** (38.5 µl, 0.232 mmol), HBTU (114 mg, 0.302 mmol, 1.3 equiv) and DIPEA (80.8 µl, 0.604 mmol, 2 equiv) were dissolved in dry DMF (2 ml). The reaction mixture was stirred at rt overnight. Diethyl ether was added, and the organic phase washed three times with water. The solvent was removed at the rotary evaporator. Recrystallization ($MeOH/H_2O$ 10+1) without chromatographic purification gave *N*-[4-(diethylamino)phenyl]-3',5-dimethyl-[3,5'-biisoxazole]-4'-carboxamide **1c** (63 mg, 0.18 mmol, 77%) as fine dark yellow needles. M.p. 123.0–124.1 °C; ^1H NMR (300 MHz, DMSO-*d₆*) δ_{ppm} 10.21 (s, 1H), 7.43 (m, 2H), 6.72 (d, 1H, *J* = 0.8 Hz), 6.66 (m, 2H), 3.31 (q, 4H, *J* = 7.0 Hz), 2.51 (s, 3H), 2.40 (s, 3H), 1.07 (t, 6H, *J* = 7.0 Hz). ^{13}C NMR (75 MHz, DMSO-d_6) δ_{ppm} 171.5, 159.4, 157.3, 156.7, 151.8, 144.6, 127.0, 121.5, 116.5, 111.8, 101.0, 43.7, 12.3, 11.7, 10.1. HRMS calcd. for $C_{19}H_{23}N_4O_3$ $[M+H]^+$: 355.1770, found 355.1773.

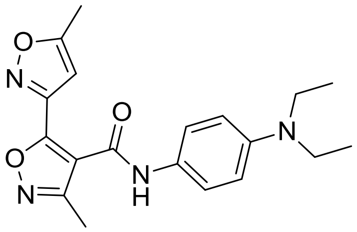

_N_-[4-(Diethylamino)phenyl]thiophene-3-carboxamide (1d, 3i-1051)

3-Thiophenecarboxylic acid **2d** (128 mg, 1.00 mmol), *N,N*-diethyl-*p*-phenylenediamine **3b** (166 µl, 1.00 mmol), HBTU (493 mg, 1.30 mmol, 1.3 equiv) and DIPEA (348.4 µl, 2.00 mmol, 2 equiv) were dissolved in dry DMF (4 ml) and reaction mixture was stirred at rt overnight. Diethyl ether was added, and the organic phase washed three times with water. The solvent was removed at the rotary evaporator. Recrystallization ($MeOH/H_2O$ 10+1) without chromatographic purification gave *N*-[4-(diethylamino)phenyl]thiophene-3-carboxamide **1d** (199 mg, 0.725 mmol, 73%) as brownish crystals. M.p. 145.7–150.6 °C; ^1H NMR (300 MHz, DMSO-d_6) δ_{ppm} 9.71 (s, 1H), 8.25 (dd≈t, 1H, ^4J = 2.1 Hz, ^4J = 2.1 Hz), 7.63–7.59 (m, 2H), 7.48 (m, 2H), 6.65 (m, 2H), 3.31 (q, 4H, ^3J = 7.0 Hz), 1.08 (t, 6H); ^{13}C NMR (75 MHz, DMSO-d_6) δ_{ppm} 160.1, 144.3, 138.2, 128.7, 127.5, 127.0, 126.5, 122.2, 111.7, 43.7, 12.4. HRMS calcd. for $C_{15}H_{19}N_2OS$ $[M+H]^+$: 275.1218, found 275.1228.

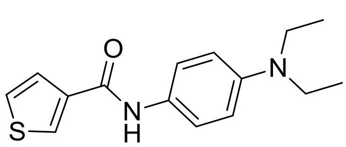

N-[4-(Diethylamino)phenyl]-5-methyl-2-phenylfuran-3-carboxamide (1e, 3i-1148)

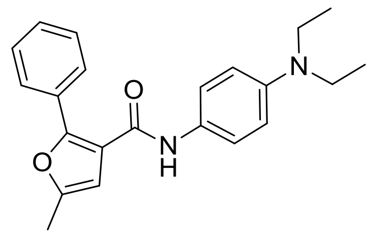

5-Methyl-2-phenylfuran-3-carboxylic acid **2e** (49.8 mg, 0.248 mmol), *N*,*N*-diethyl-*p*-phenylenediamine **3b** (41.1 μl, 0.248 mmol), HBTU (122 mg, 0.322 mmol, 1.3 equiv) and DIPEA (86.4 μl, 0.496 mmol, 2 equiv) were dissolved in dry DMF (1 ml) and reaction mixture was stirred at rt overnight. Diethyl ether was added, and the organic phase washed three times with water. The solvent was removed at the rotary evaporator, and the crude product mixture was subjected to a purification by an automated high performance flash chromatography system (*n*-hexane/EtOAc 0→100%) to yield *N*-[4-(diethylamino)phenyl]-5-methyl-2-phenylfuran-3-carboxamide **1e** (75.9 mg, 0.218 mmol, 88%) as grey crystals. M.p. 141.5–143.1 °C (decomp.); ^{1}H NMR (300 MHz, DMSO-d_6) δ_{ppm} 9.70 (s, 1H), 7.83–7.77 (m, 2H), 7.47–7.27 (m, 5H), 6.62 (m, 2H), 6.56 (d, 1H, ^4J = 0.9 Hz,), 3.28 (q, 4H, ^3J = 7.0 Hz), 2.36 (d, 3H), 1.06 (t, 6H); ^{13}C NMR (75 MHz, DMSO-d_6) δ_{ppm} 161.4, 150.6, 150.3, 144.2, 130.0, 128.2, 127.9, 127.6, 126.1, 121.9, 119.4, 111.8, 108.4, 43.7, 13.0, 12.3. HRMS calcd. for $C_{22}H_{25}N_2O_2$ [M+H]$^+$: 349.1916, found 349.1916.

N-[4-(Diethylamino)phenyl]-4,5-dimethyl-2-(1H-pyrrol-1-yl)thiophene-3-carboxamide (1f, 3i-1165)

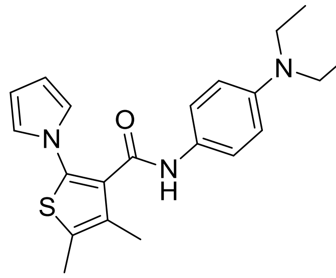

A mixture of 4,5-dimethyl-2-(1*H*-pyrrol-1-yl)thiophene-3-carboxylic acid **2f** (25.0 mg, 0.113 mmol), *N*,*N*-diethyl-*p*-phenylenediamine **3b** (18.8 μl, 0.113 mmol), HBTU (55.7 mg, 147 μmol, 1.3 equiv) and DIPEA (39.4 μl, 0.226 mmol, 2 equiv) in dry DMF (1 ml) was stirred at rt overnight. An equimixture of diethyl ether and ethyl acetate was added, and the organic phase washed three times with an equimixture of water and brine. The solvent was removed at the rotary evaporator, and the crude product mixture was subjected to a purification by an automated high performance flash chromatography system (*n*-hexane/EtOAc 0→100%) to yield *N*-[4-(diethylamino)phenyl]-4,5-dimethyl-2-(1*H*-pyrrol-1-yl)thiophene-3-carboxamide **1f** (30.5 mg, 83.0 μmol, 73%) as a white solid. M.p. 145.1–146.6 °C (decomp.); ^{1}H NMR (300 MHz, DMSO-d_6):δ_{ppm} 7.10 (m, 2H), 6.90 (t, 2H, J = 2.1 Hz), 6.74 (br s, 1H), 6.58 (m, 2H), 3.30 (q, 4H, ^3J = 7.1 Hz), 2.35 (s, 3H), 2.30 (s, 3H), 1.12 (t, 6H); ^{13}C NMR (75 MHz, DMSO-d_6) δ_{ppm} 161.7, 145.6, 137.6, 132.7, 131.3, 129.7, 126.3, 123.5, 122.7, 112.3, 111.2, 44.7, 13.2, 13.0, 12.7. HRMS calcd. for $C_{21}H_{26}N_3OS$ [M+H]$^+$: 368.1797, found 368.1797.

5-Methyl-3-phenylisoxazol-4-amine (10)

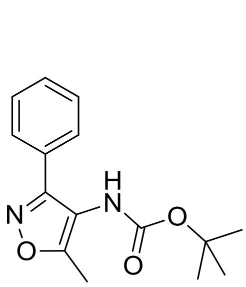

i. tert-Butyl (5-methyl-3-phenylisoxazol-4-yl)carbamate: 5-Methyl-3-phenylisoxazole-4-carboxylic acid (8.000 g, 39.37 mmol) was dissolved under argon in *tert*-butanol (70 ml). Triethylamine (5.488 ml, 39.37 mmol) and diphenyl phosphoryl azide (8.512 ml, 39.37 mmol) were added and the reaction mixture was heated at reflux temperature for two hours. After cooling to rt, EtOAc was added and the organic phase was washed three times with water. The combined aqueous phases were extracted once with EtOAc and the combined EtOAc phases were washed twice with a 1 M solution of NaOH in H_2O and with small portions of water until the water remained neutral. The solvent was removed at the rotary evaporator and recrystallized twice (MeOH/H_2O, 10+1). The solvent of the mother liquor was removed at the rotary evaporator and the residue was purified by automated chromatography on silica gel with an increasing gradient of EtOAc in hexane, starting with 0% of EtOAc. The combined material from crystallizations and chromatography was recrystallized (MeOH/H_2O, 10+1) to yield *tert*-butyl (5-methyl-3-phenylisoxazol-4-yl) carbamate as white needles (6.768 g, 63%). M.p. 116.8–119.9 °C; ^{1}H NMR (300 MHz, $CDCl_3$) δ_{ppm} 7.83–7.66 (m, 2H), 7.52–7.40 (m, 3H), 5.65 (s, 1H), 2.41 (s, 3H). ^{13}C NMR (75 MHz, $cdcl_3$) δ_{ppm} 159.43, 154.14, 129.92, 128.95, 128.66, 127.74, 81.28, 28.27, 11.28. HRMS calc. for $C_{15}H_{19}N_2O_3$ [M+H]$^+$: 275.1396, found 275.1396.

ii. 5-Methyl-3-phenylisoxazol-4-amine (**10**): *tert*-Butyl (5-methyl-3- phenylisoxazol-4-yl)carbamate (5.259 g, 19.17 mmol) was dissolved in trifluoroacetic acid (20 ml) and stirred at rt overnight. Diethylether and water were added and solution was made basic with a 10 M solution of NaOH in H_2O. Phases were separated and aqueous phase was extracted with diethyl ether. Organic phase was washed three times with water and The solvent was removed at the rotary evaporator, and the crude product mixture was subjected to a purification by an automated high performance flash chromatography system (*n*-hexane/EtOAc 0→100%) to yield 5-methyl-3-phenylisoxazol-4-amine **10** (1.73 g, 52%). ^{1}H NMR (300 MHz, DMSO-d_6) δ_{ppm} 7.99–7.75 (m, 2H), 7.54 – 7.40 (m, 3H), 3.96 (s, 1H), 2.34 (s, 3H). ^{13}C NMR (75 MHz, DMSO-d_6) δ_{ppm} 155.69, 153.39, 129.44, 129.08, 128.65, 128.65, 127.15, 121.96, 9.88. HRMS calc. for $C_{10}H_{11}N_2O$ [M+H]$^+$: 175.0871, found 175.0891.

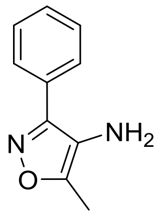

(*E*)-*N*,*N*-Diethyl-4-[[(5-methyl-3-phenylisoxazol-4-yl)imino]methyl]aniline (8)

4-(Diethylamino)benzaldehyde **10** (366 mg, 2.07 mmol) and 5-methyl-3-phenylisoxazol-4-amine **9** (360 mg, 2.07 mmol) were dissolved in dry toluene (4 ml). Na_2SO_4 (587 mg, 4.13 mmol, 2.0 equiv) and AcOH (29.6 µL, 0.517 mmol, 0.25 equiv) were added and reaction mixture was stirred at rt overnight. The solvent was removed at the rotary evaporator, and the crude product mixture was subjected to a purification by an automated high performance flash chromatography system (*n*-hexane/EtOAc 0→100%) to yield (*E*)-*N*,*N*-diethyl-4-[[(5-methyl-3-phenylisoxazol-4-yl)imino]methyl]aniline **8** (514 mg, 1.54 mmol, 74%) as yellowish crystals. M.p. 119.7–120.6 °C; ^{1}H NMR (300 MHz, DMSO-d_6) δ_{ppm} 8.36 (s, 1H), 7.95–7.87 (m, 2H), 7.96 (m, 2H), 7.53–7.43 (m, 3H), 6.75 (m, 2H), 3.42 (q, 4H, ^3J = 7.0 Hz), 2.47 (s, 3H), 1.13 (t, 6H); ^{13}C NMR (75 MHz, DMSO-d_6) δ_{ppm} 163.2, 156.6, 156.3, 150.1, 130.3, 129.5, 128.9, 128.6, 126.6, 127.0, 126.1, 122.7, 110.9, 43.8, 12.4, 11.0. HRMS calcd. for $C_{21}H_{24}N_3O$ [M+H]$^+$: 334.1919, found 334.1918.

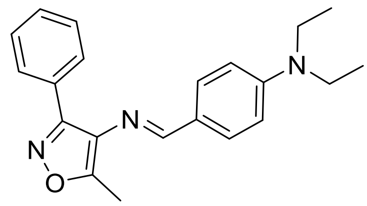

***N*-[4-(Diethylamino)benzyl]-5-methyl-3-phenylisoxazol-4-amine (11)**

(*E*)-*N*,*N*-Diethyl-4-[[(5-methyl-3-phenylisoxazol-4-yl)imino]methyl]aniline **8** (450 mg, 1.35 mmol) was dissolved in an equimixture of absolute methanol and absolute THF (8 ml). Sodium borohydride (76.6 mg, 2.03 mmol, 1.5 equiv) was added to the solution, and the reaction mixture was stirred at rt overnight. Another batch of sodium borohydride (76.6 mg, 2.03 mmol, 1.5 equiv) was added, and the reaction mixture was stirred at rt overnight. The reaction mixture was diluted with ethyl acetate, and the organic phase was extracted with an equimixture of water and brine. The solvent was removed *in vacuo*, and the crude product mixture was subjected to a purification by an automated high performance flash chromatography system (*n*-hexane/EtOAc 0→100%) to yield *N*-[4-(diethylamino)benzyl]-5-methyl-3-phenylisoxazol-4-amine **11** (166 mg, 0.495 mmol, 37%) as yellowish oil. ^{1}H NMR (300 MHz, DMSO-d_6) δ_{ppm} 7.93–7.84 (m, 2H), 7.55–7.44 (m, 3H), 6.94 (m, 2H), 6.55 (m, 2H), 4.25 (t, 1H, ^3J = 6.8 Hz), 3.70 (d, 2H), 3.28 (q, 4H, ^3J = 7.0 Hz), 2.18 (s, 3H), 1.04 (t, 6H); ^{13}C NMR (75 MHz, DMSO-d_6) δ_{ppm} 159.2, 157.4, 146.5, 129.4, 129.3, 129.0, 128.6, 127.0, 126.1, 123.2, 111.4, 51.5, 43.6, 12.3, 10.1. HRMS calcd. for $C_{21}H_{26}N_3O$ [M+H]$^+$: 336.2076, found 336.2076.

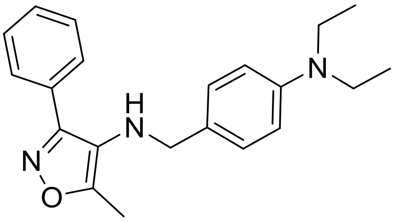

N-[4-(Diethylamino)benzyl]-N-(5-methyl-3-phenylisoxazol-4-yl)propionamide (1g, 3i-1120)

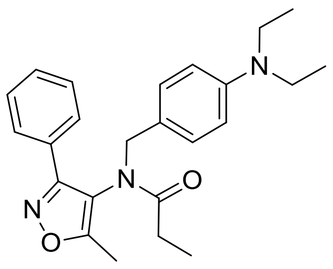

N-[4-(Diethylamino)benzyl]-5-methyl-3-phenylisoxazol-4-amine **11** (27.6 mg, 82.3 μmol) was dissolved under argon in absolute pyridine (300 μl). Propionyl chloride (14.4 μl, 0.165 mmol, 2 equiv) and DMAP (10.1 mg, 82.3 μmol) were added to the solution, and the resulting mixture was stirred for at rt for 3 d. The reaction mixture was diluted with EtOAc and washed with a saturated solution of $NaHCO_3$ in H_2O and water. The solvent was removed *in vacuo*, and the crude product mixture was subjected to a purification by an automated high performance flash chromatography system (*n*-hexane/EtOAc 0→100%) to yield *N*-[4-(diethylamino)benzyl]-*N*-(5-methyl-3-phenylisoxazol-4-yl)propionamide **1h** (23.8 mg, 60.8 μmol, 74%) as a colorless oil. ^{1}H NMR (300 MHz, $CDCl_3$) δ_{ppm} 7.74–7.66 (m, 2H), 7.49–7.40 (m, 3H), 6.96 (m, 2H), 6.52 (m, 2H), 5.48 (d, 1H, ^2J = 13.7 Hz), 3.66 (d,1H), 3.31 (q, 4H, ^3J = 7.1 Hz), 2.10 (q, 2H, ^3J = 7.4 Hz), 1.75 (s, 3H), 1.12 (t, 6H), 1.08 (t, 3H); ^{13}C NMR (75 MHz, $CDCl_3$) δ_{ppm}170.3, 167.8, 158.3, 147.7, 131.1, 130.4, 129.3, 128.1, 126.8, 123.4, 117.3, 112.0, 51.1, 44.5, 27.5, 12.6, 10.3, 9.4. HRMS calcd. for $C_{24}H_{30}N_3O_2$ $[M+H]^+$: 392.2338, found 392.2338.

^{1}H NMR and ^{13}C spectra for the compounds

(E/Z)-Benzaldehyde oxime (**4a**)

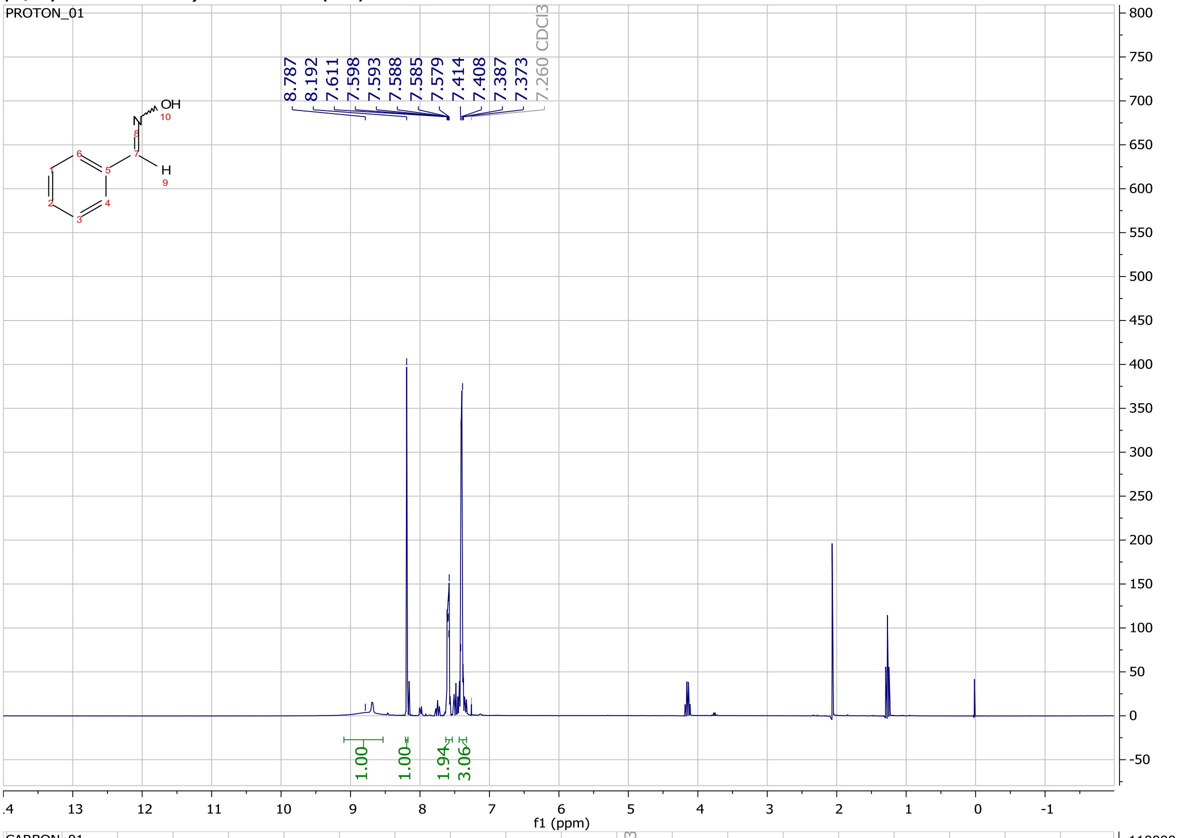

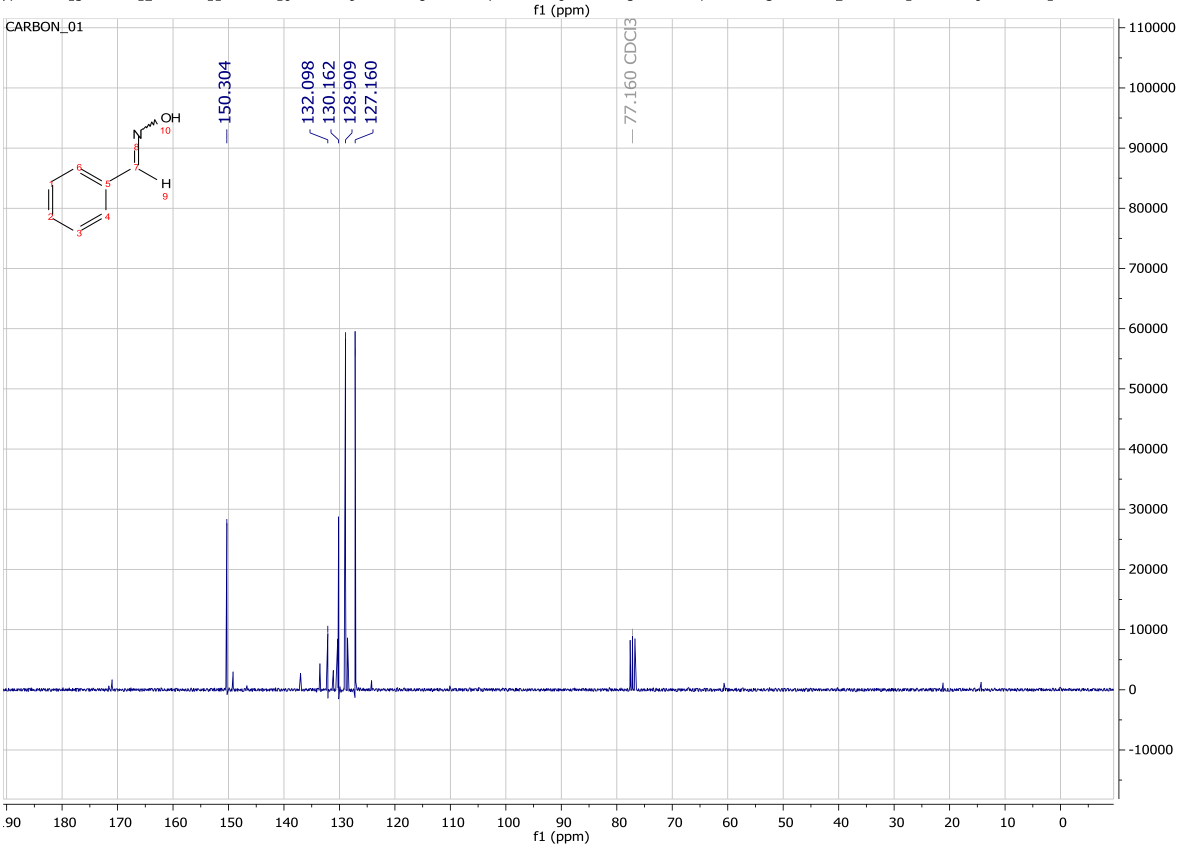

Ethyl 5-methyl-3-phenylisoxazole-4-carboxylate (**6a**)

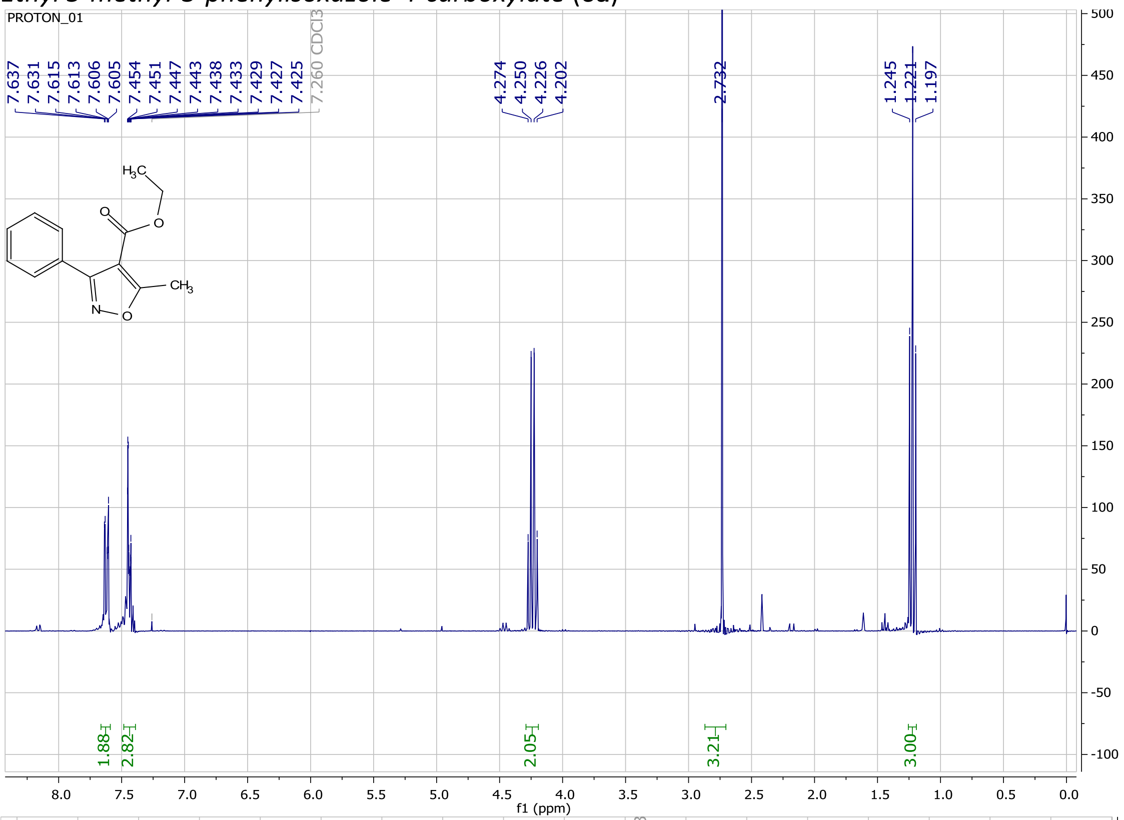

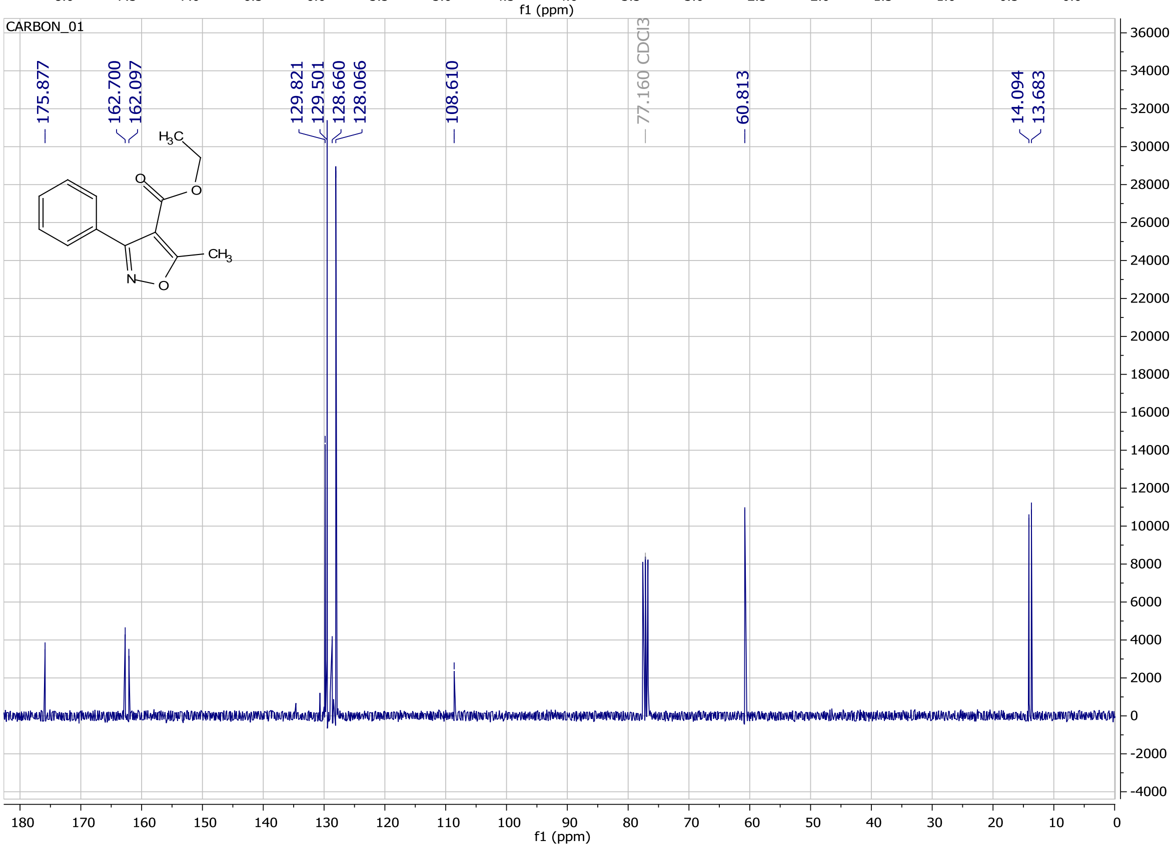

5-Methyl-3-phenylisoxazole-4-carboxylic acid (**2a**)

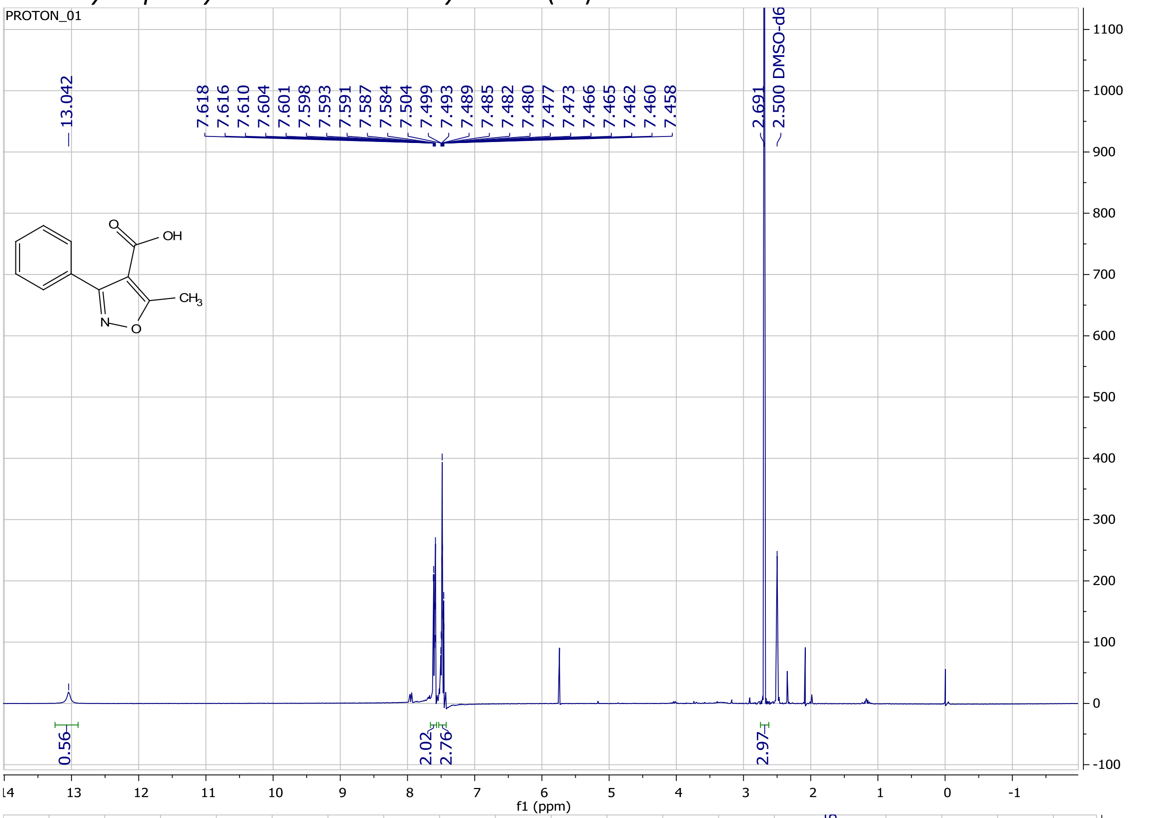

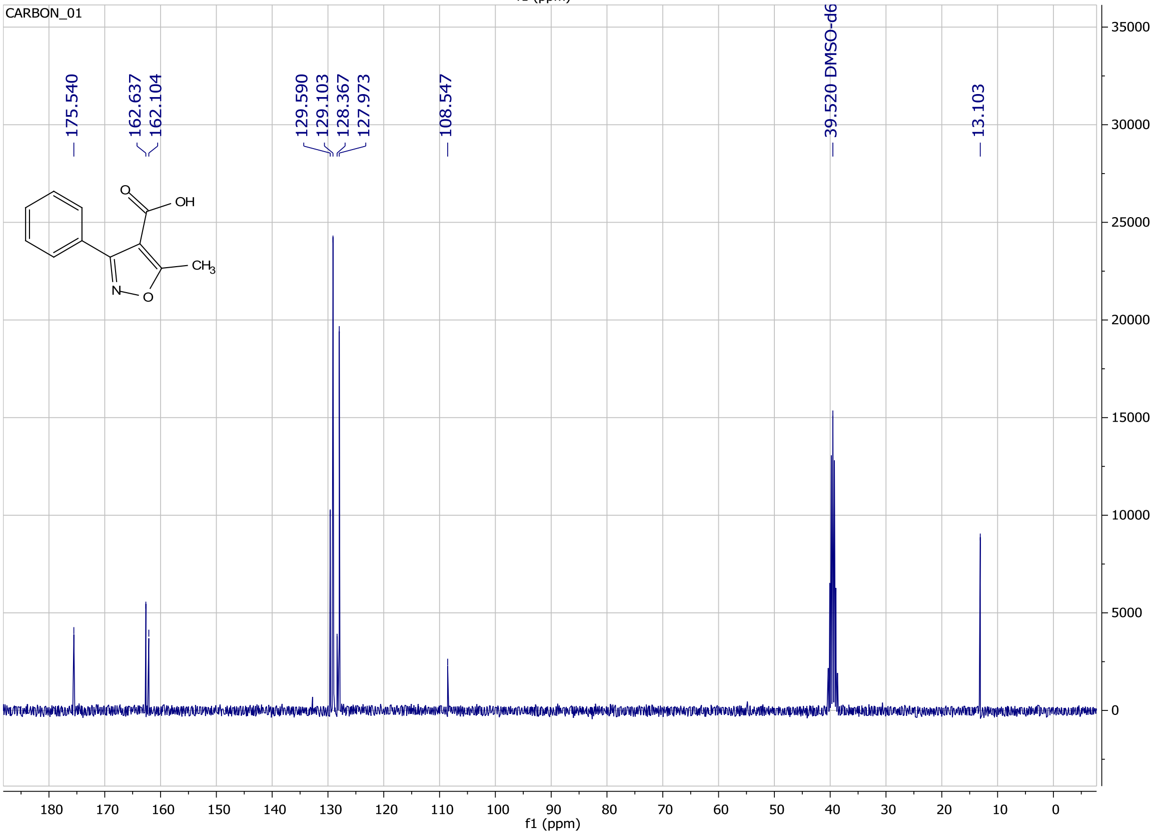

[3-Amino-5-(4-methoxyphenyl)-1H-pyrazol-1-yl](5-methyl-3-phenylisoxazol-4-yl)methanone (**1a**, **3i-1229**)

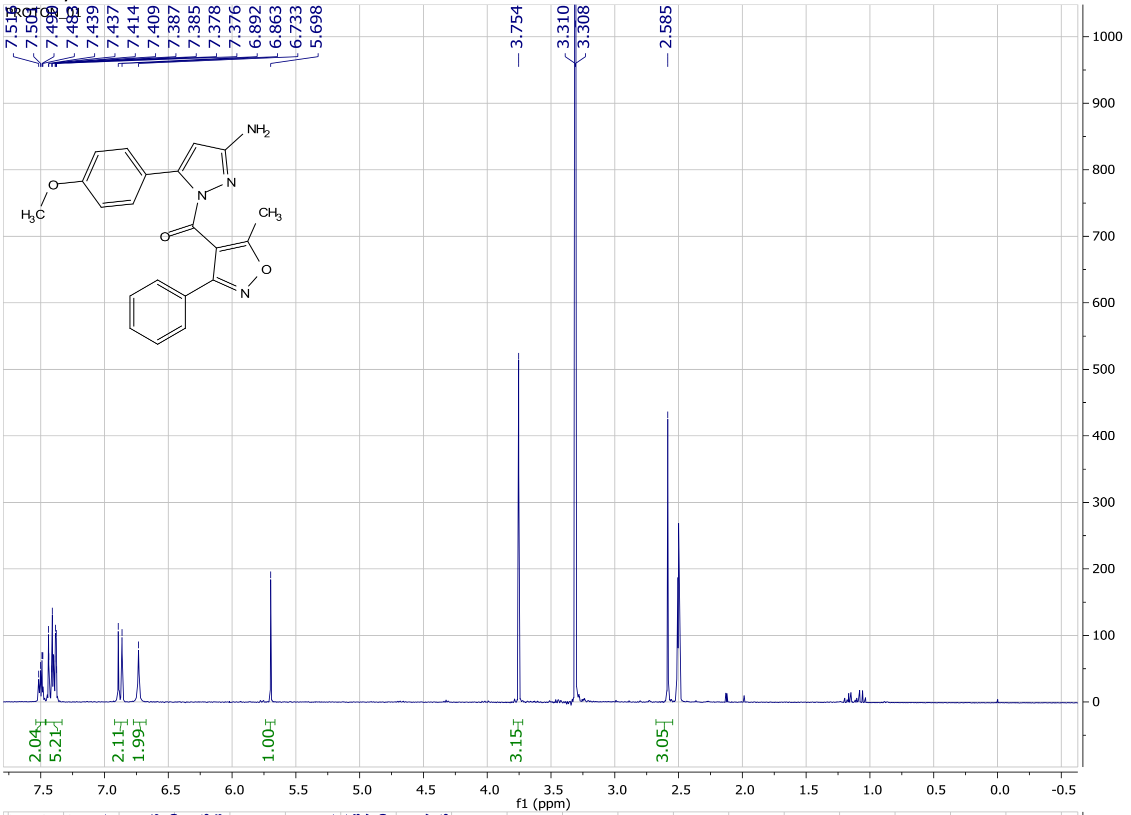

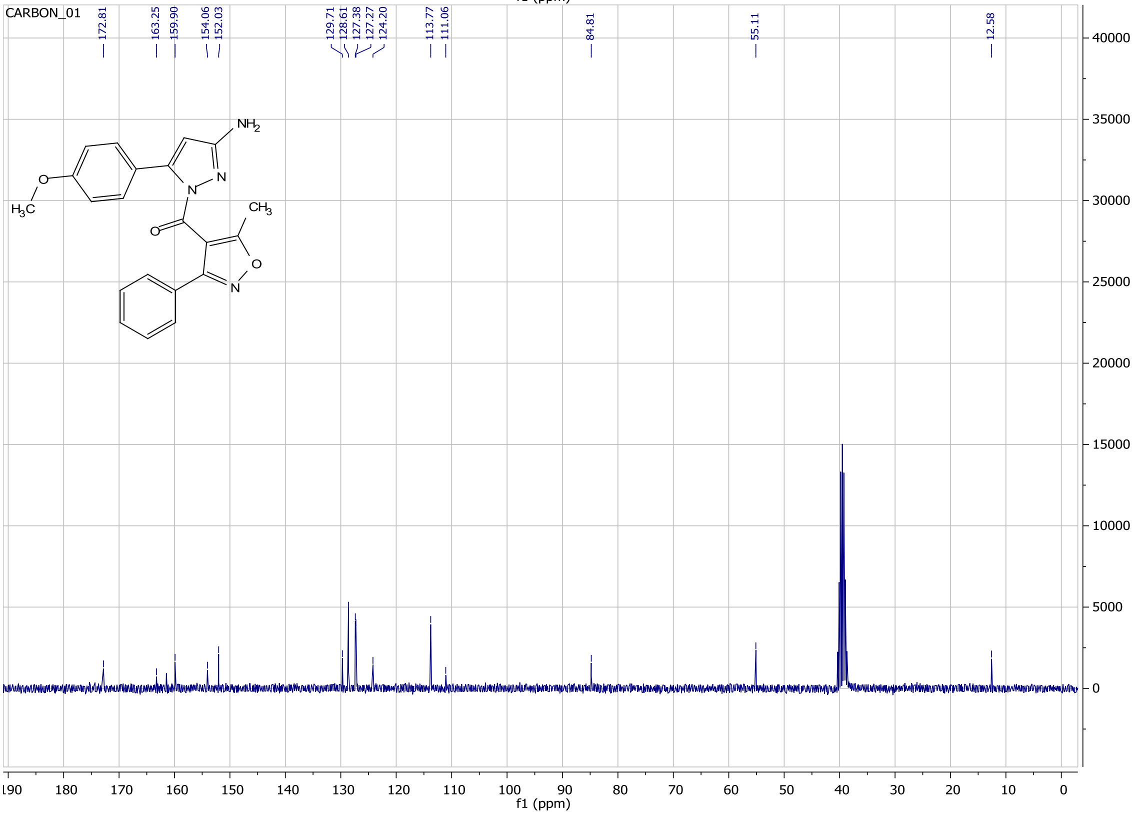

(E/Z)-2-Methyloxazole-4-carbaldehyde oxime (**6b**)

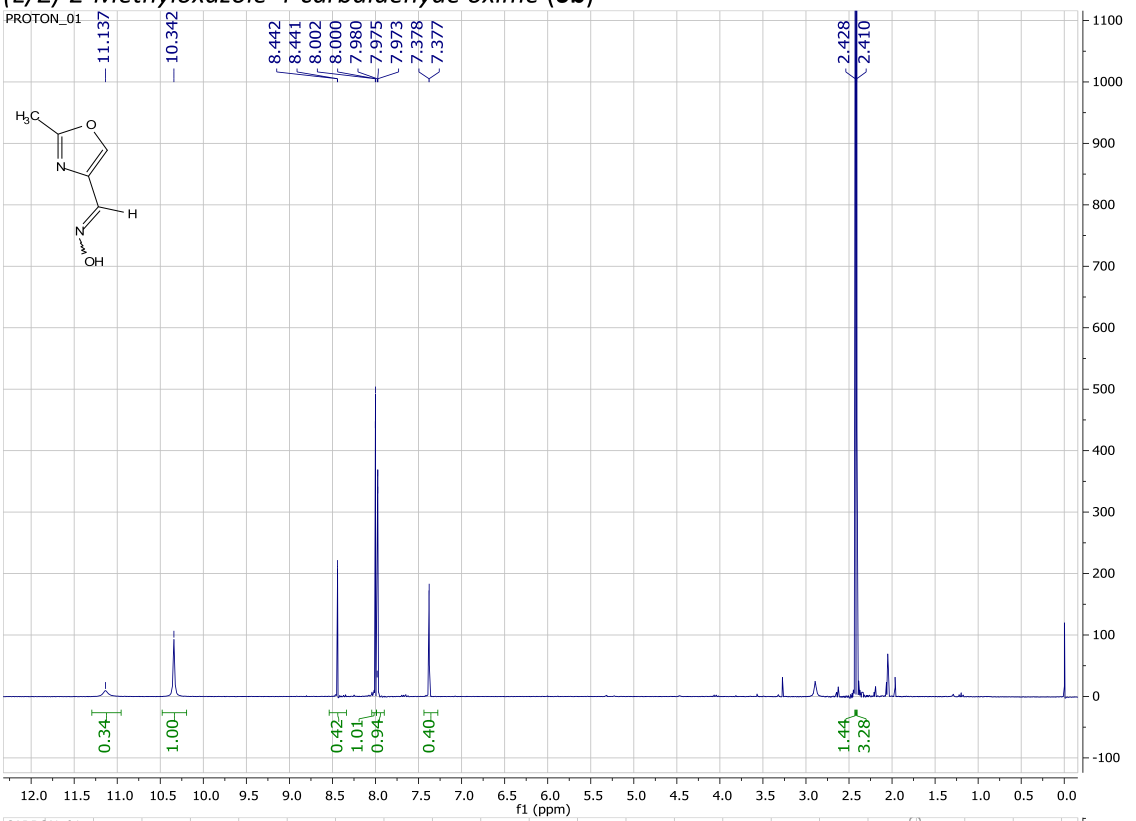

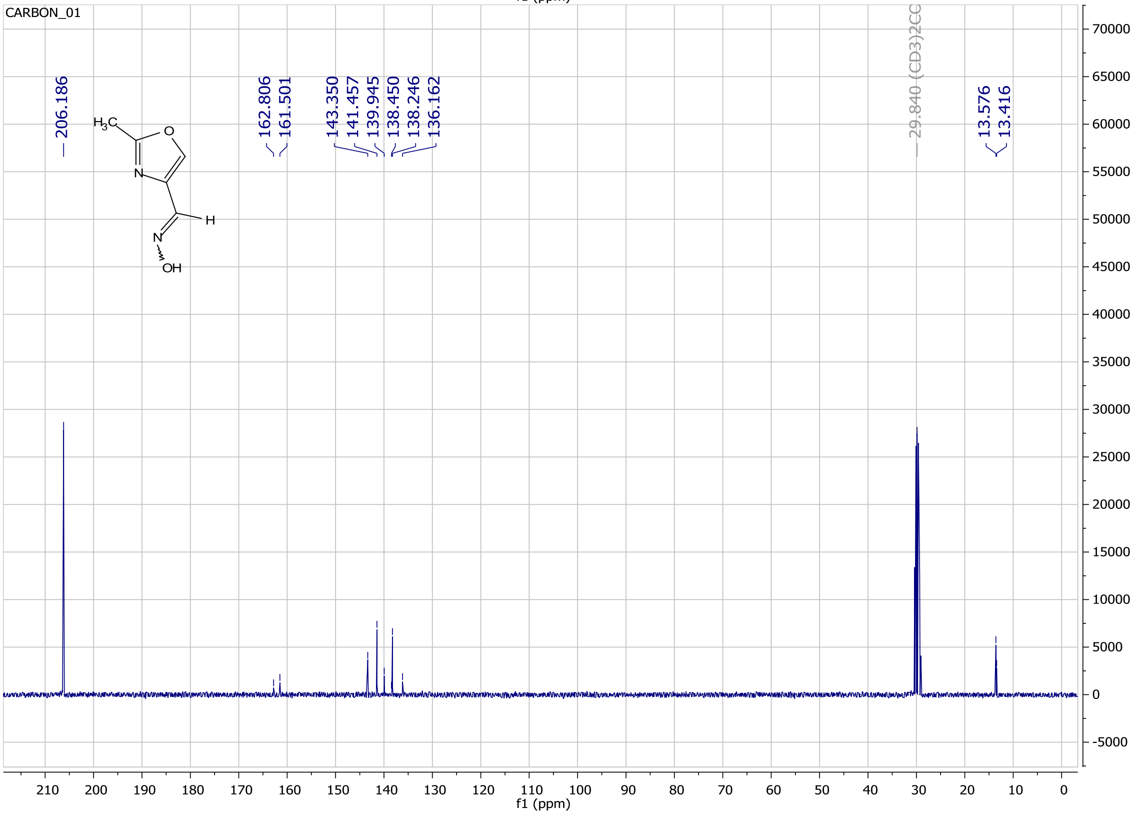

N-[4-(Diethylamino)phenyl]-5-methyl-3-(2-methyloxazol-4-yl)isoxazole-4-carboxamide (**1b**)

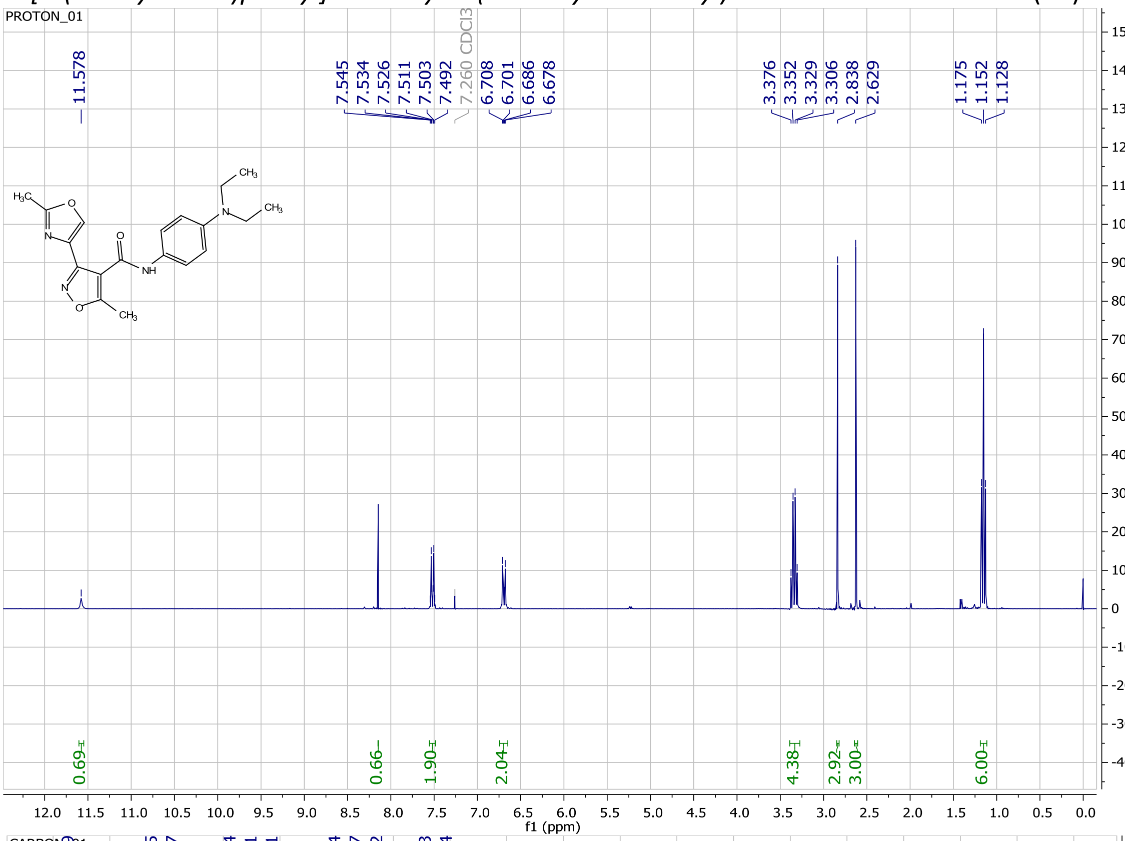

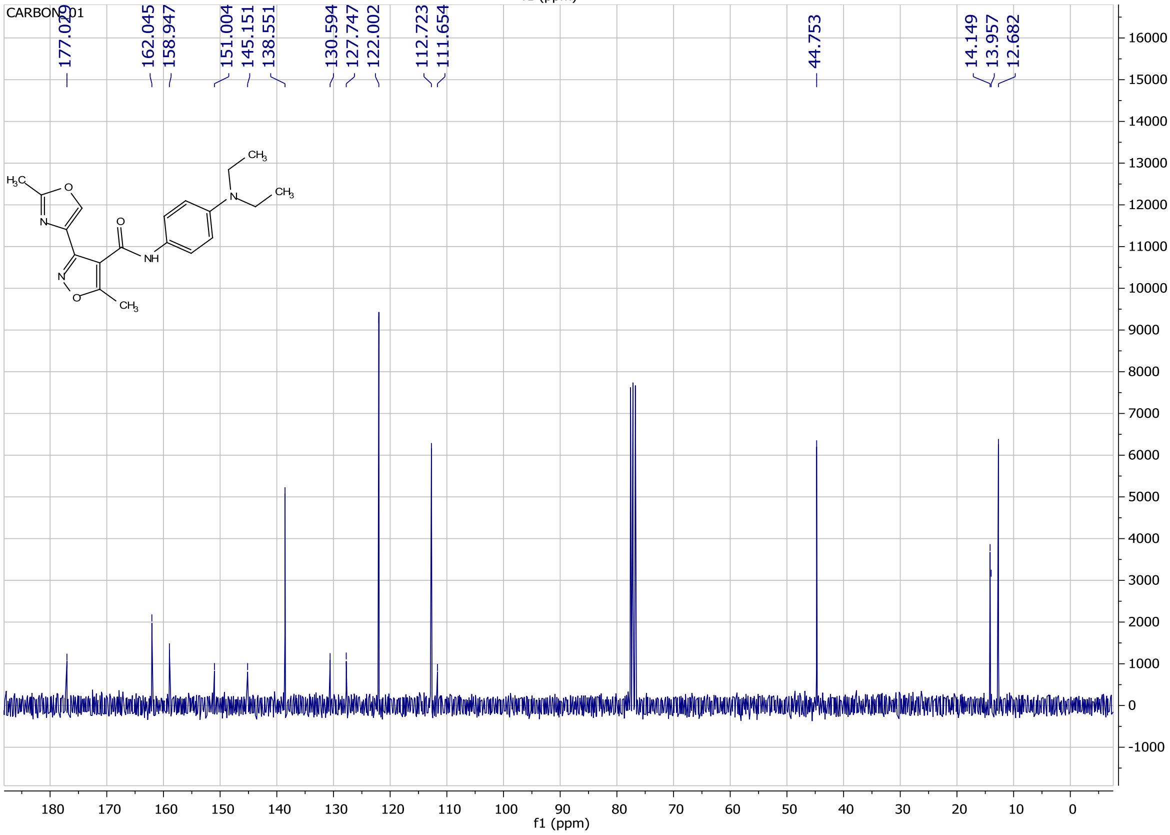

N-[4-(Diethylamino)phenyl]-3',5-dimethyl-[3,5'-biisoxazole]-4'-carboxamide (**1c, 3i-1047**)

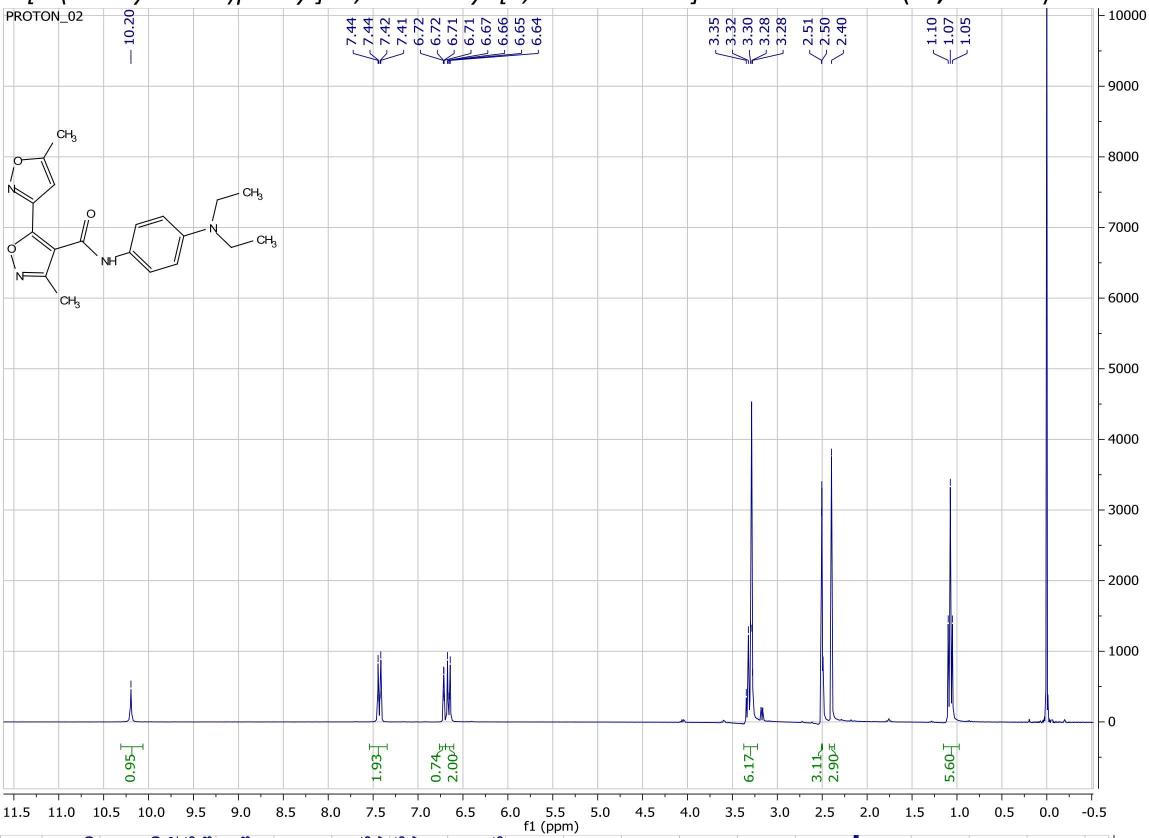

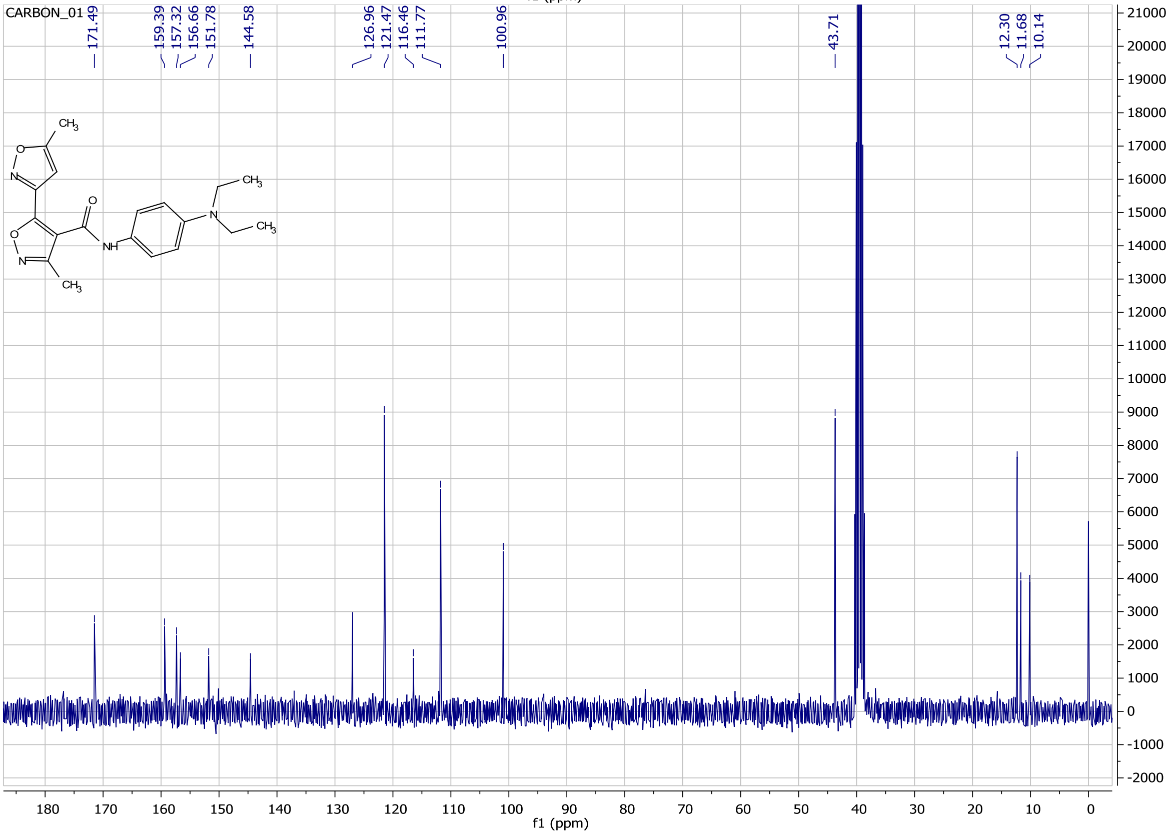

N-[4-(Diethylamino)phenyl]thiophene-3-carboxamide (**1d, 3i-1051**)

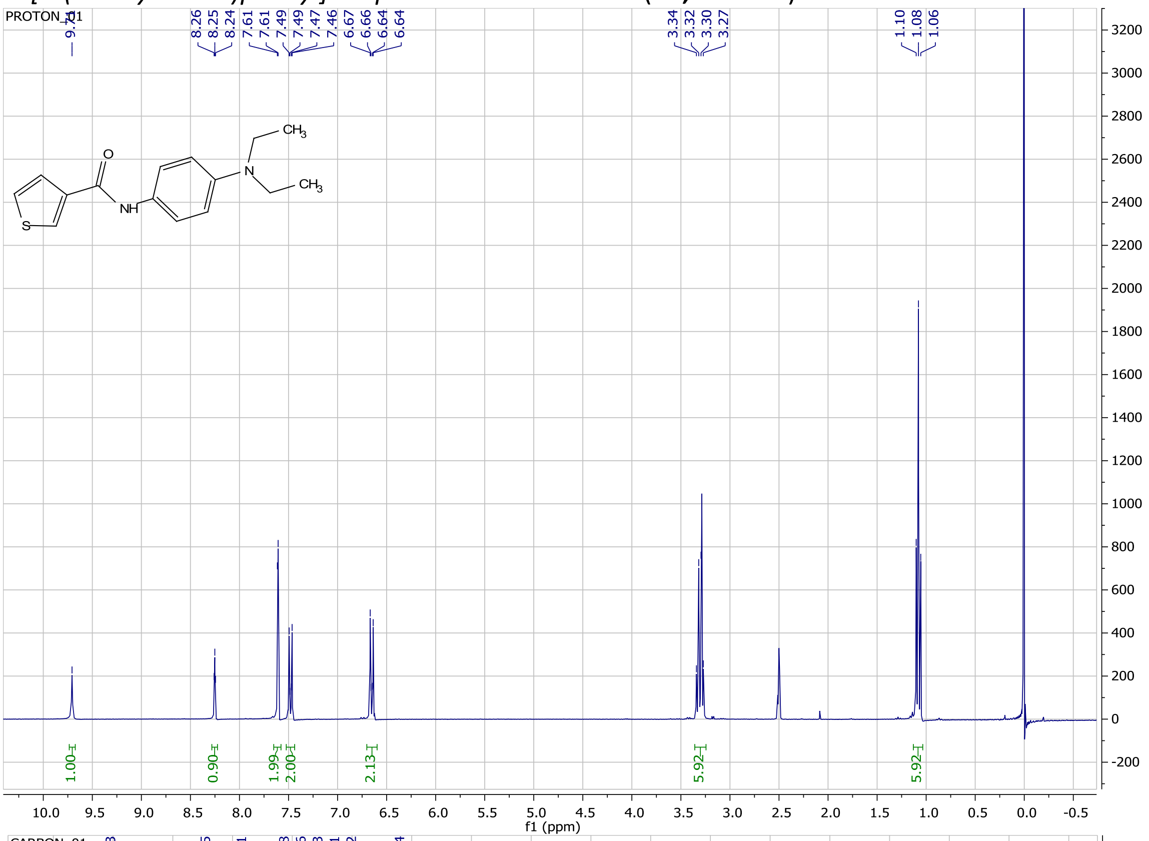

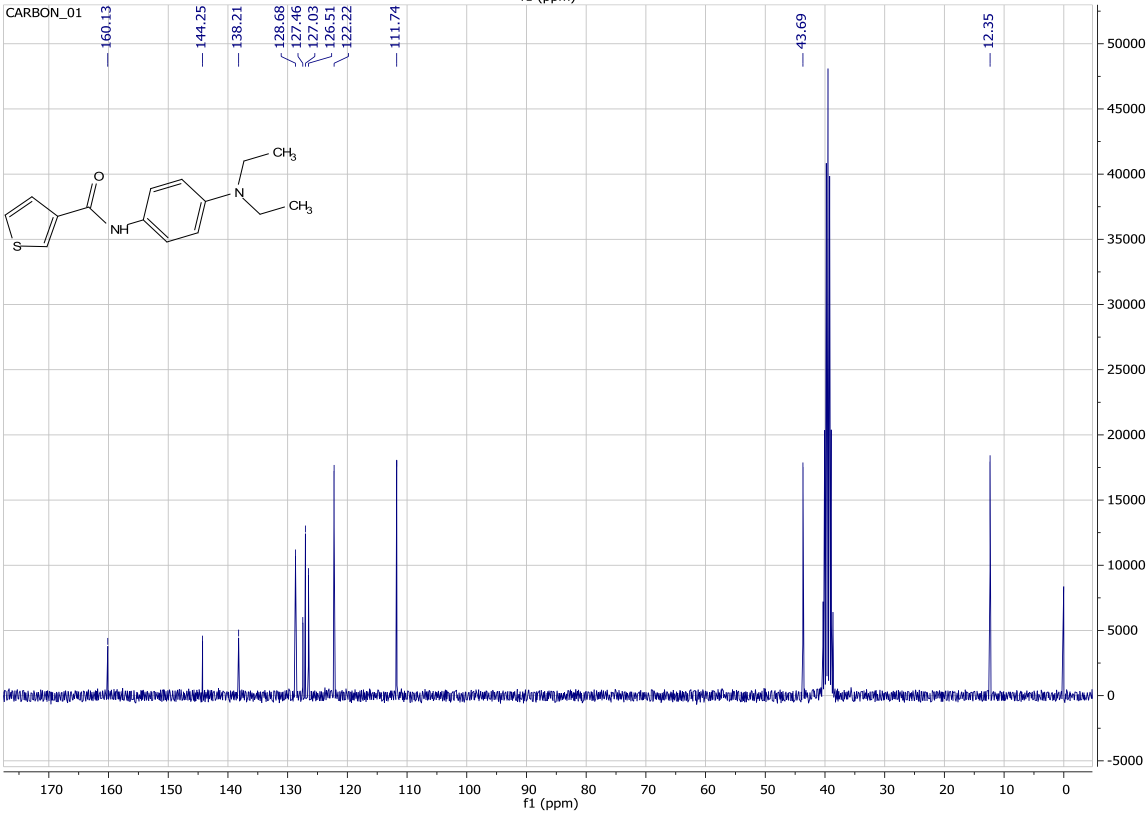

N-[4-(Diethylamino)phenyl]-5-methyl-2-phenylfuran-3-carboxamide (**1e, 3i-1148**)

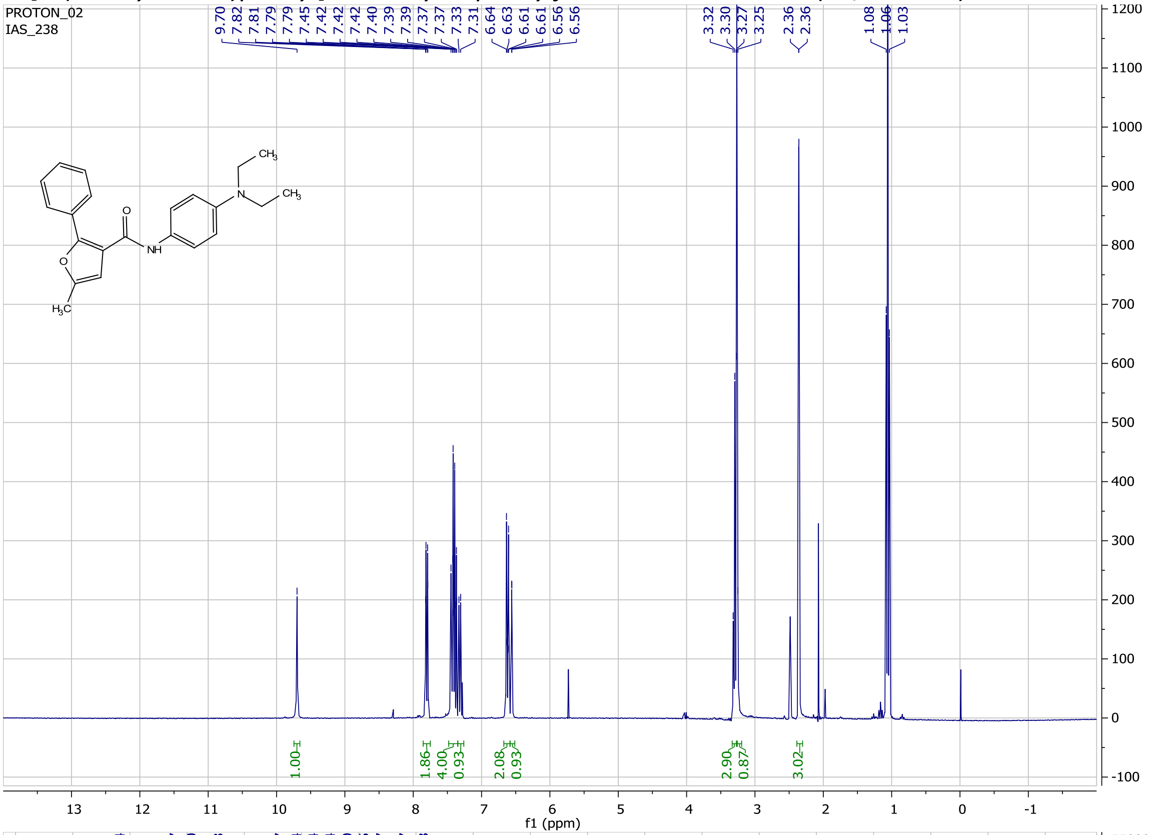

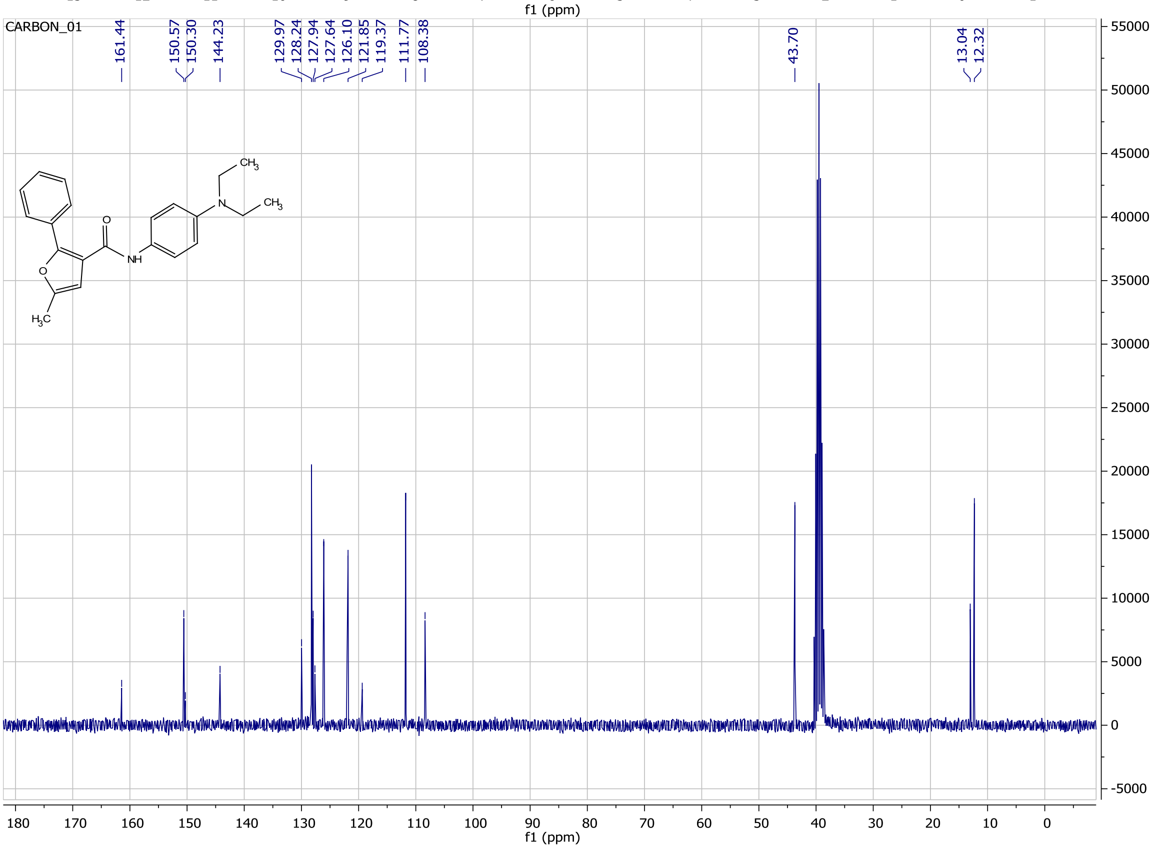

N-[4-(Diethylamino)phenyl]-4,5-dimethyl-2-(1H-pyrrol-1-yl)thiophene-3-carboxamide (**1f, 3i-1165**)

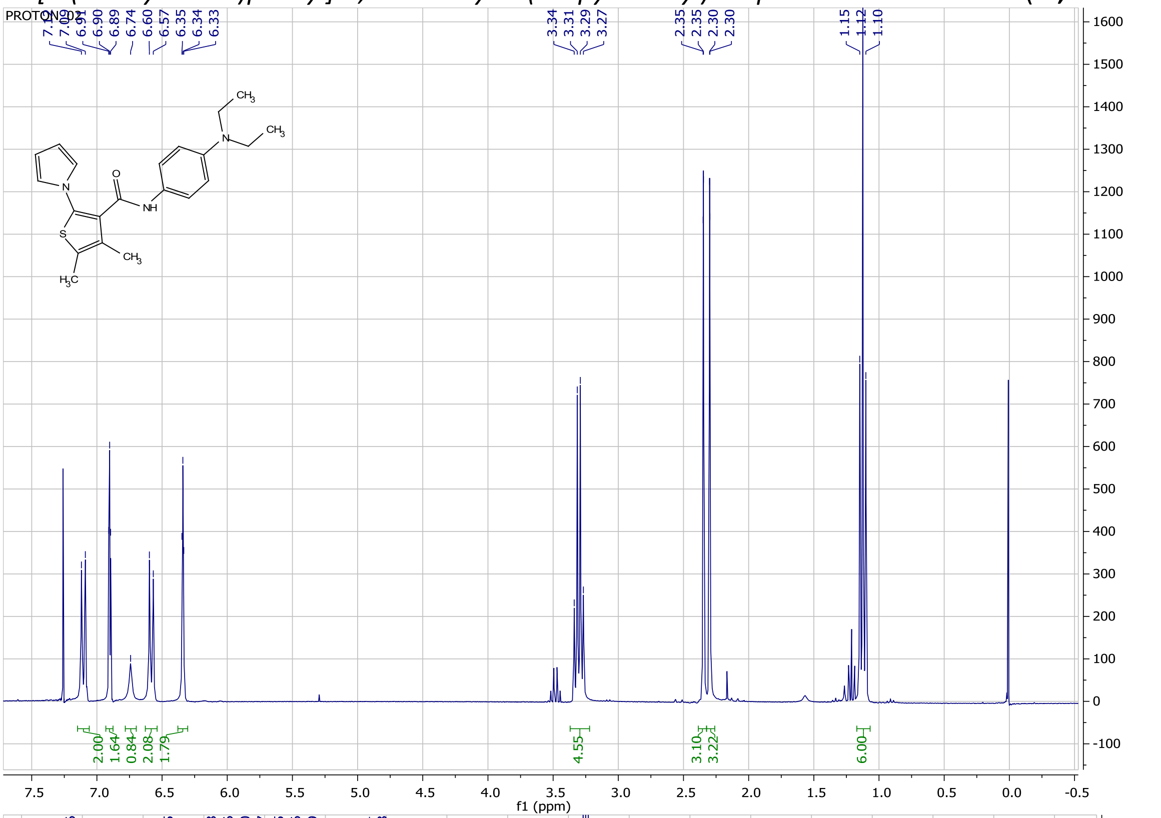

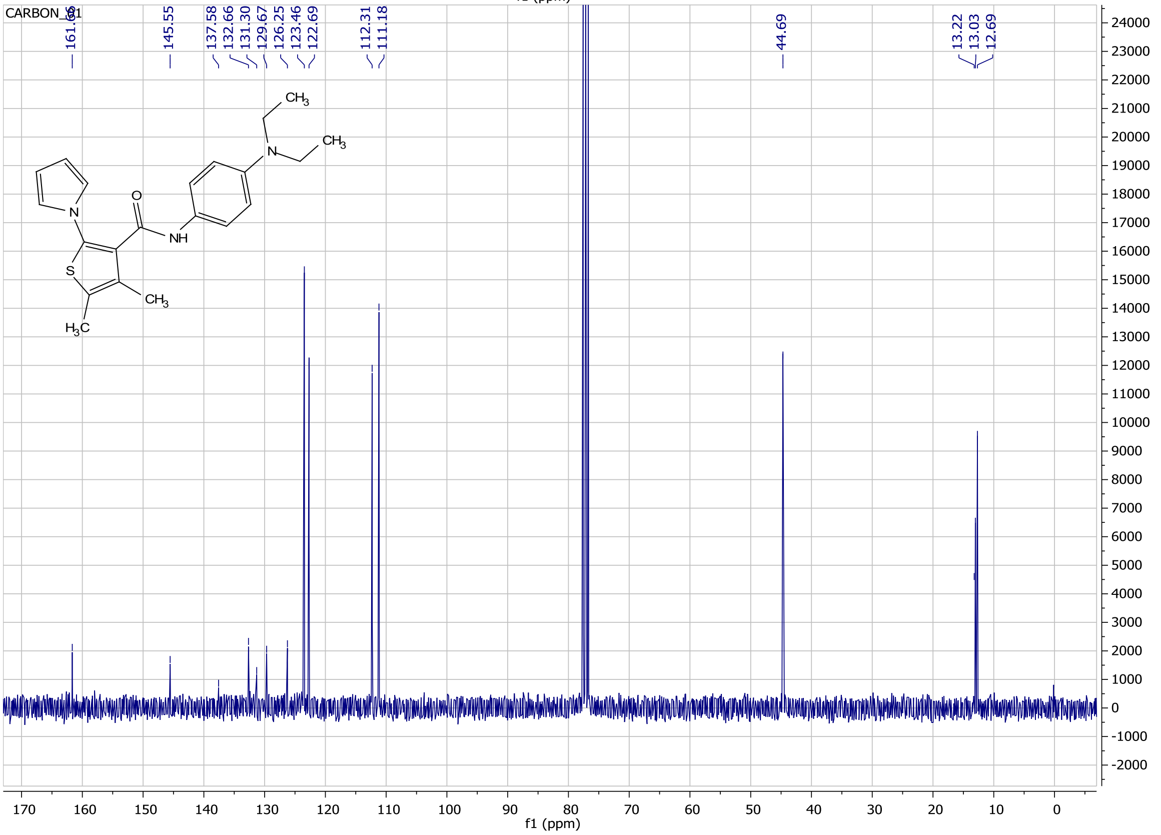

tert-Butyl (5-methyl-3-phenylisoxazol-4-yl)carbamate

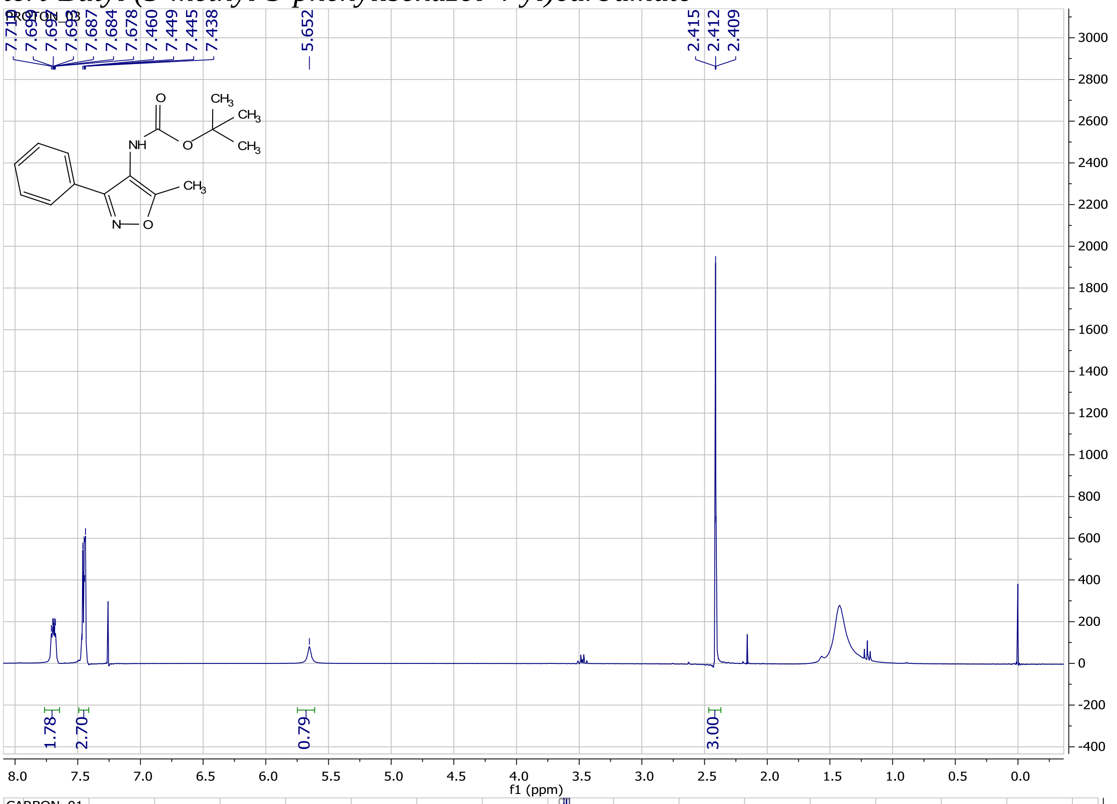

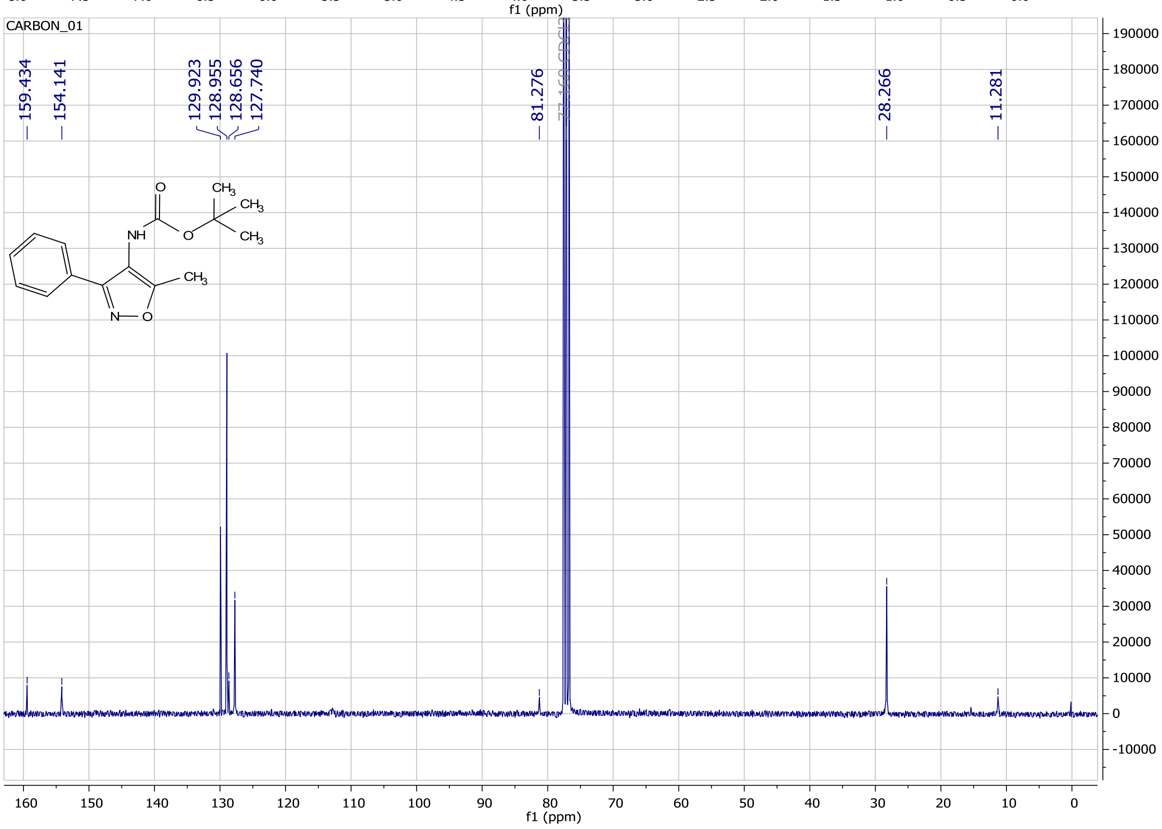

5-Methyl-3-phenylisoxazol-4-amine (**10**)

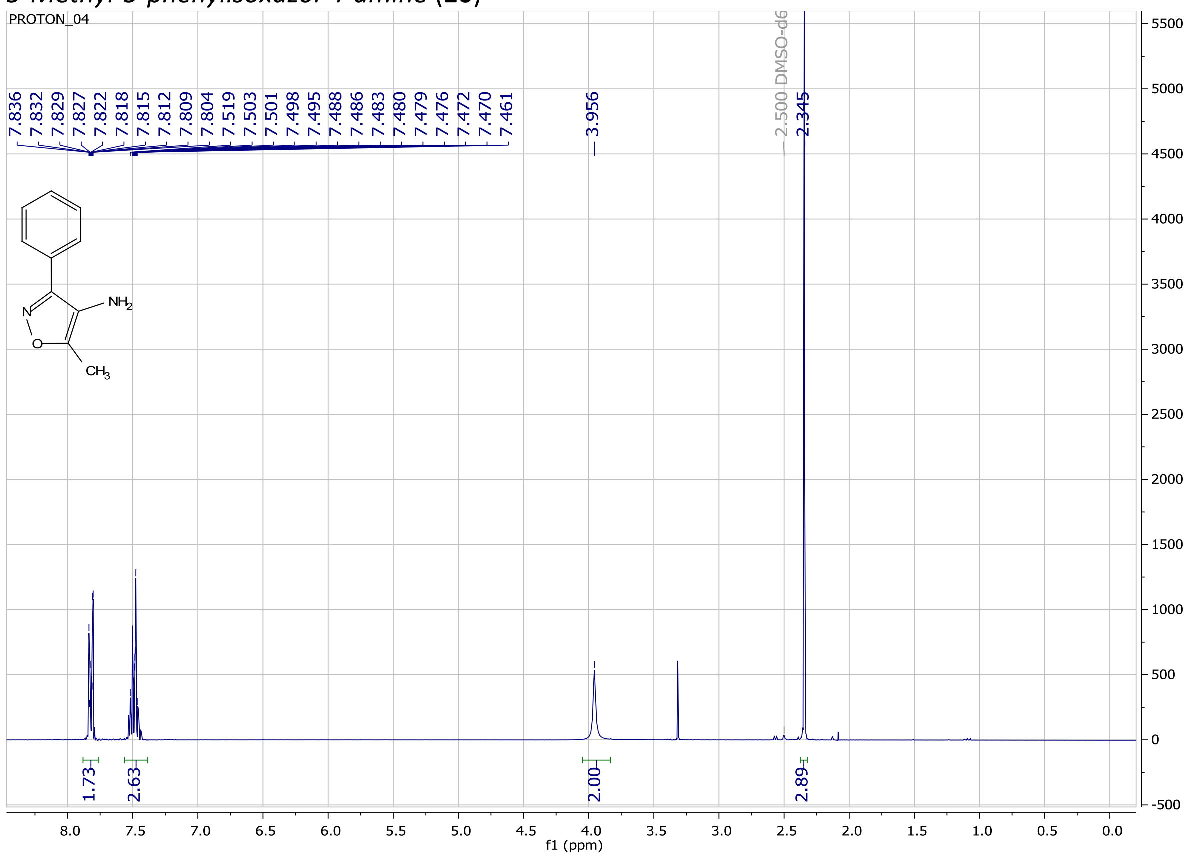

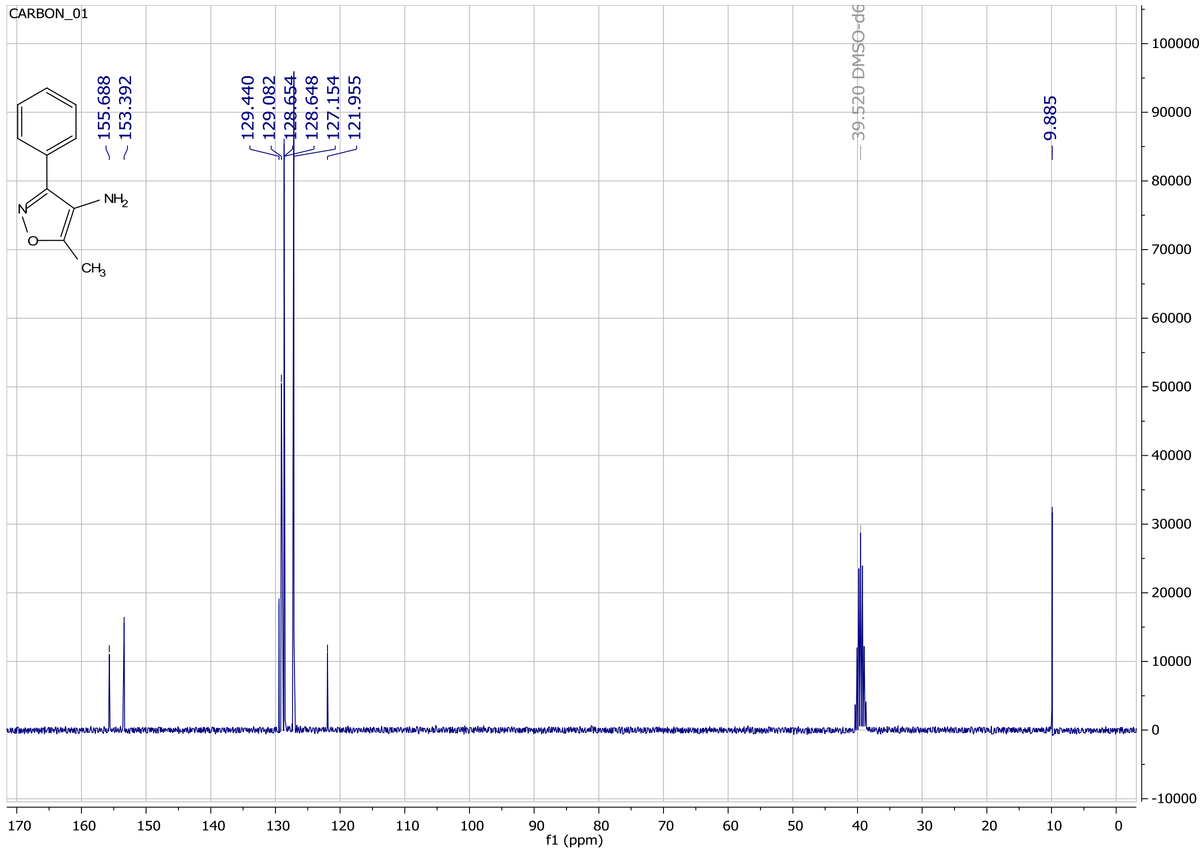

(E)-N,N-Diethyl-4-[[(5-methyl-3-phenylisoxazol-4-yl)imino]methyl]aniline (**8**)

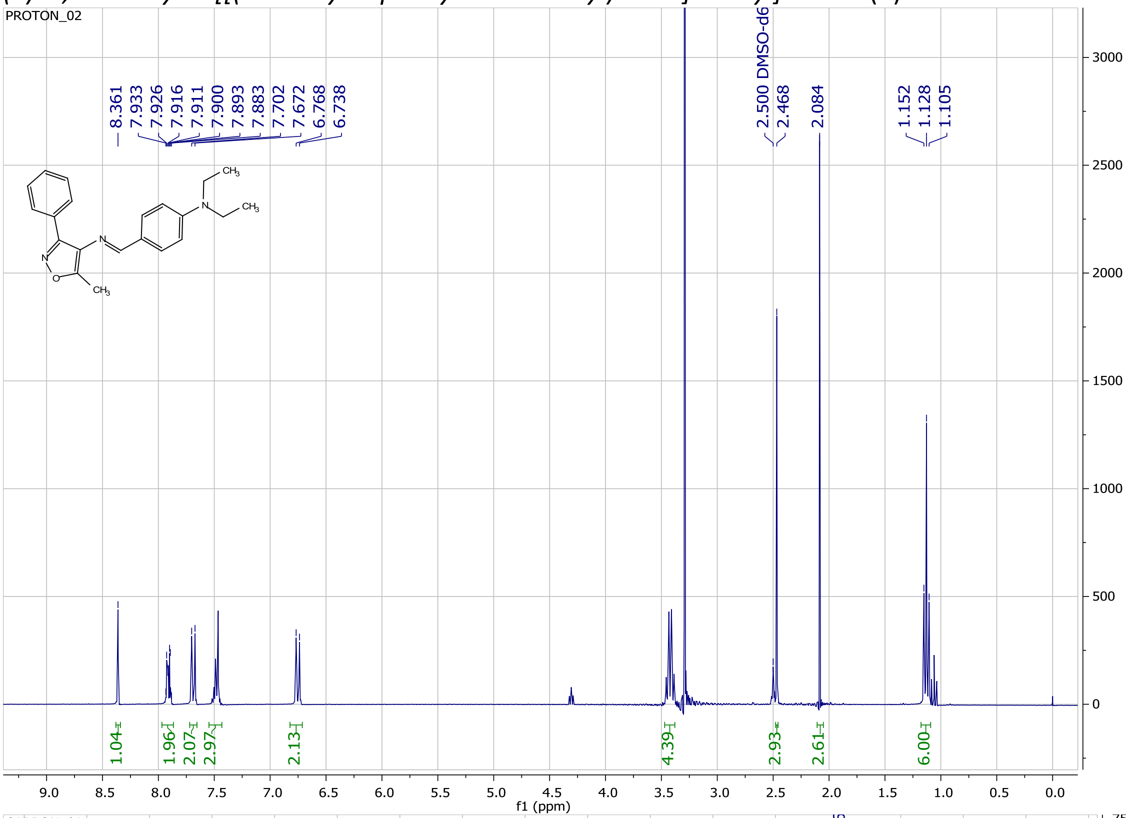

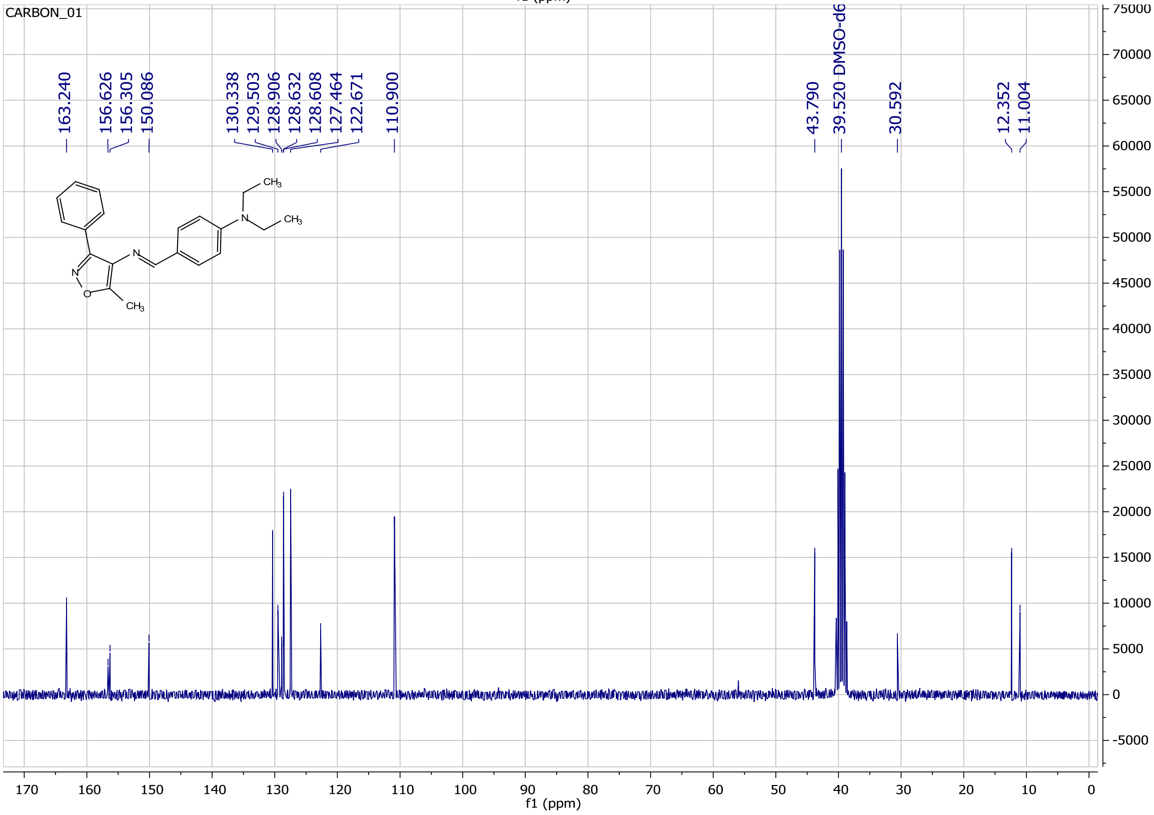

N-[4-(Diethylamino)benzyl]-5-methyl-3-phenylisoxazol-4-amine (**11**)

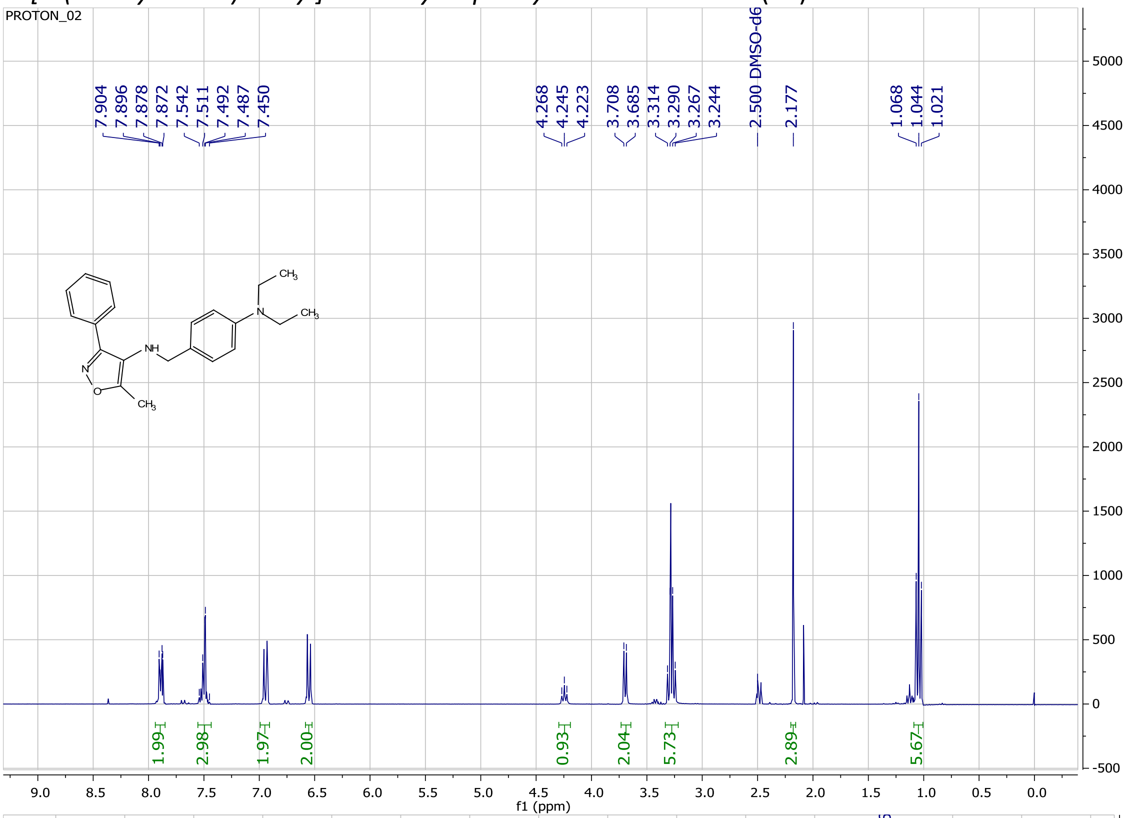

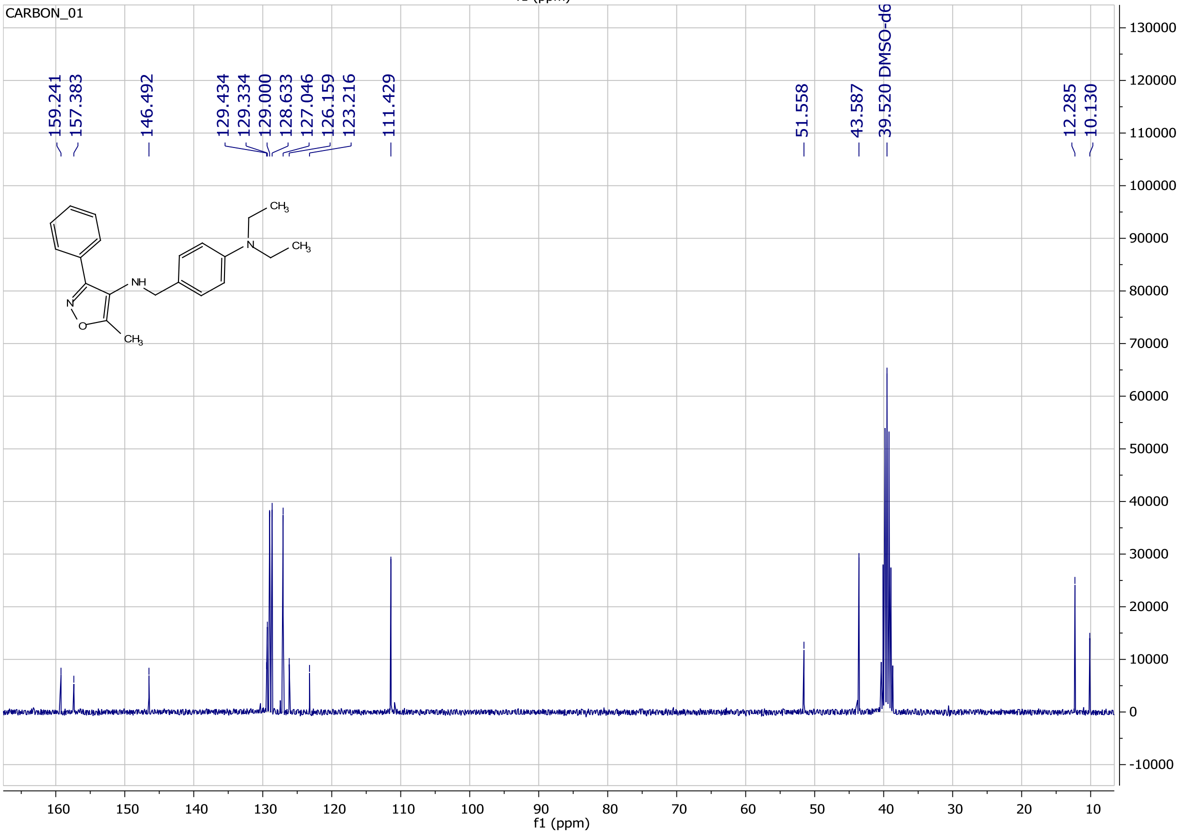

N-[4-(Diethylamino)benzyl]-N-(5-methyl-3-phenylisoxazol-4-yl)propionamide (**1g, 3i-1120**)

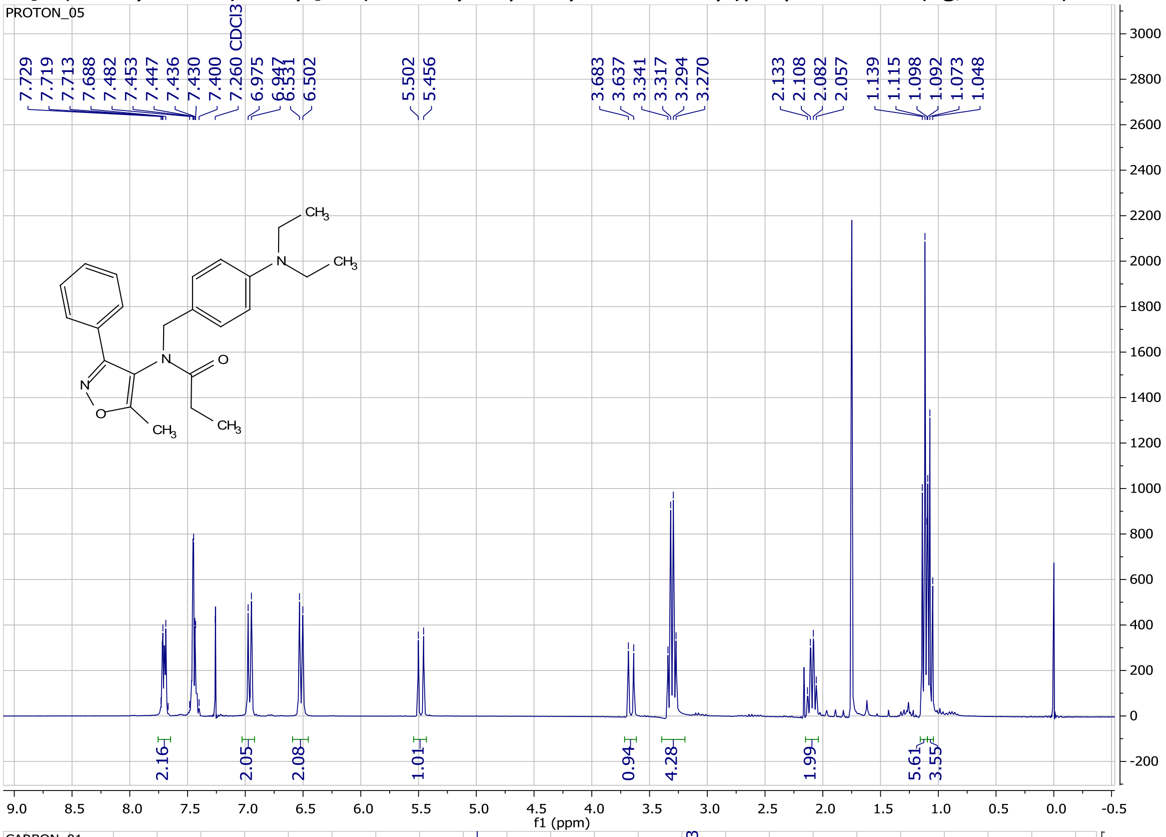

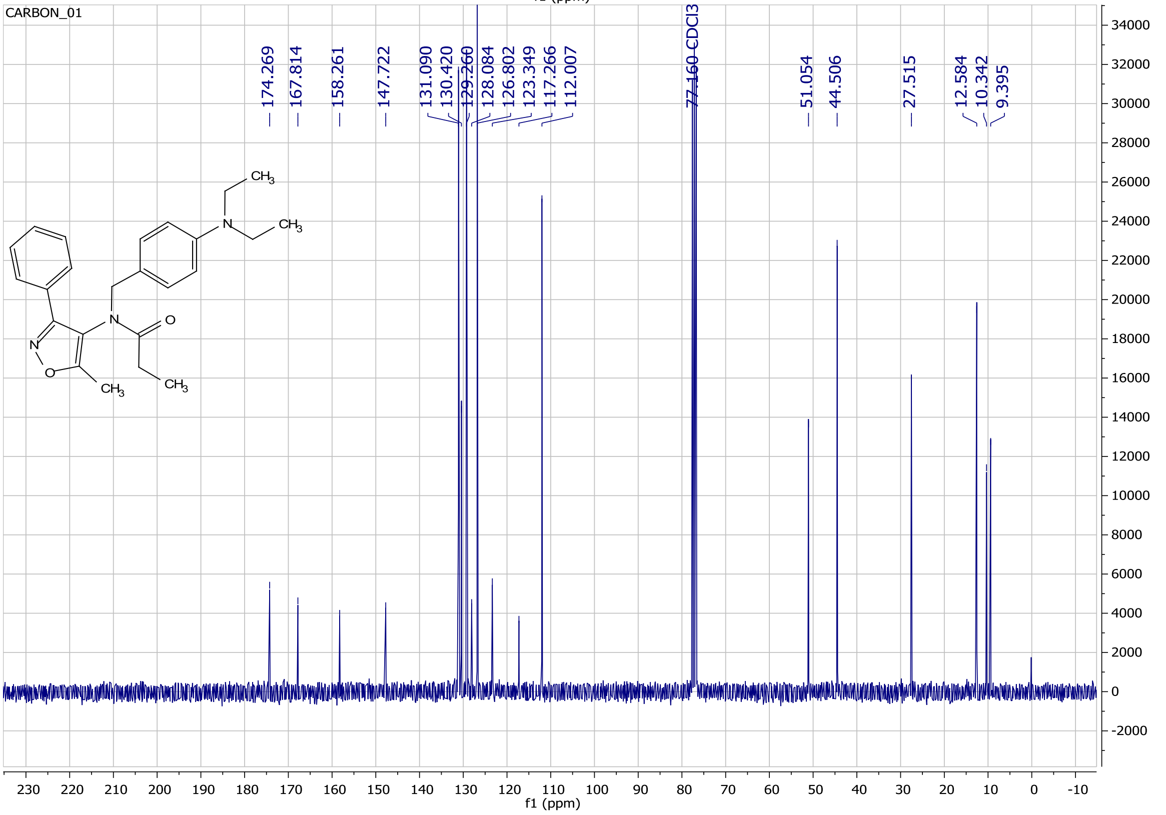

References

Burridge PW, Matsa E, Shukla P, Lin ZC, Churko JM, Ebert AD, Lan F, Diecke S, Huber B, Mordwinkin NM, Plews JR, Abilez OJ, Cui B, Gold JD, Wu JC (2014) Chemically defined generation of human cardiomyocytes. Nat Methods 11:855–860

Polinger IS (1970) Separation of cell types in embryonic heart cell cultures. Exp Cell Res 63:78–82

Tölli MA, Ferreira MP, Kinnunen SM, Rysä J, Mäkilä EM, Szabo Z, Serpi RE, Ohukainen PJ, Välimäki MJ, Correia AM, Salonen JJ, Hirvonen JT, Ruskoaho HJ, Santos HA (2014) In vivo biocompatibility of porous silicon biomaterials for drug delivery to the heart. Biomaterials 35:8394–8405

Yu J, Vodyanik MA, Smuga-Otto K, Antosiewicz-Bourget J, Frane JL, Tian S, Nie J, Jonsdottir GA, Ruotti V, Stewart R, Slukvin II, Thomson JA (2007) Induced pluripotent stem cell lines derived from human somatic cells. Science 318:1917–1920
